# CpG hypermethylation and WNT/AP-1 cooperativity define the epigenetic landscape and a clinical subgroup of high-risk pediatric adrenocortical carcinoma

**DOI:** 10.1038/s41467-026-77225-5

**Published:** 2026-09-14

**Authors:** Victoria E. Fincke, Maurice Loßner, Marina Kunstreich, Nic G. Reitsam, Irmengard Sax, Marlena Mucha, Felix Dorn, Lorenz C. Helmschrott, Maria D. Hernandez Ramirez, Sebastian Dintner, Konstantin Okonechnikov, Martin Sill, Ina Oehme, Heike Peterziel, Enrique Blanco-Carmona, Eva Sipos, Stefan Wudy, Christoph Slavetinsky, Jörg Fuchs, Bruno Märkl, Eva Jüttner, Christian Vokuhl, Michael C. Frühwald, Antje Redlich, Stefan Pfister, Matthias Schlesner, Rainer Claus, Michaela Kuhlen, Pascal D. Johann

**Affiliations:** 1 Pediatrics and Adolescent Medicine, Swabian Children’s Cancer Center, Faculty of Medicine, University of Augsburg, Augsburg, Germany; 2 Bavarian Cancer Research Center (BZKF) and KIONET Bayern, Augsburg, Germany; 3 Pediatric Hematology and Oncology, University Hospital Magdeburg, Magdeburg, Germany; 4 Pathology, Faculty of Medicine, University of Augsburg, Augsburg, Germany; 5 Else Kroener Fresenius Center for Digital Health, Faculty of Medicine and University Hospital Carl Gustav Carus, TUD Dresden University of Technology, Dresden, Germany; 6 Faculty of Applied Computer Sciences, Biomedical Informatics, Data Mining and Data Analytics, University of Augsburg, Augsburg, Germany; 7 Hopp Children’s Cancer Center Heidelberg (KiTZ), Heidelberg University Hospital, German Cancer Research Center (DKFZ), National Center for Tumor Diseases (NCT) and German Cancer Consortium (DKTK), Baden-Wuerttemberg, Germany, Germany; 8 Pediatric Endocrinology & Diabetology, Children’s Hospital, Justus Liebig University, Giessen, Germany; 9 Department of Pediatric Surgery and Urology, University Children´s Hospital, Tuebingen, Germany; 10 Pathology, University Hospital Schleswig-Holstein, Kiel, Germany; 11 Section of Pediatric Pathology, Department of Pathology, University Hospital Bonn, Bonn, Germany; 12 Division of Pediatric Neurooncology, German Cancer Research Center (DKFZ) and German Cancer Consortium (DKTK), Heidelberg, Germany; 13 Department of Pediatric Hematology and Oncology, Heidelberg University Hospital, Heidelberg, Germany; 14 National Center for Tumor Diseases (NCT) Heidelberg, Heidelberg, Germany; 15 Hematology and Oncology, Medical Faculty, University of Augsburg, Augsburg, Germany

**Keywords:** Paediatric cancer, Biomarkers, Tumour heterogeneity

## Abstract

Pediatric adrenocortical tumors are rare, clinically heterogeneous neoplasms with unpredictable outcomes and limited treatment options. Through integrated multi-omic analysis of 214 pediatric adrenocortical tumors combining DNA methylation profiling, transcriptomics, chromatin accessibility, and spatial deconvolution, we identify four distinct risk groups. A high-risk subgroup is characterized by CpG island hypermethylation, chromosomal instability, and dismal survival. These tumors exhibit transcriptional co-activation of WNT signalling and activator protein-1 transcriptional programs and display balanced admixture of zona glomerulosa and zona fasciculata/reticularis-like cells. Spatial analysis reveals zona glomerulosa cells as WNT signaling hubs driving intercellular crosstalk. Mechanistically, the histone deacetylase inhibitor entinostat reverses promoter methylation, silences activator protein-1 activity, and induces apoptotic reprogramming in tumor models. These findings establish a molecular framework for risk stratification and identify actionable therapeutic vulnerabilities, providing an essential resource for studying this molecularly uncharted pediatric malignancy.

## Abstract (web summary)

Pediatric adrenocortical tumours (pACTs) are rare and difficult to treat. Here, the authors perform integrated multi-omics analysis of 214 pACTs and identify four distinct risk groups and potential therapeutic targets.

## Introduction

Pediatric adrenocortical tumors (pACTs) are rare endocrine neoplasms characterized by biological complexity and clinical heterogeneity, making therapeutic management particularly challenging. In non-metastatic cases, predicting disease course remains difficult due to a lack of molecular risk markers. While most pACTs are diagnosed in early childhood and present with endocrine symptoms, their biological behavior ranges from indolent to highly aggressive, with survival rates dropping to 15.6% in metastatic cases^1^. Both overtreatment and undertreatment remain concerns.

Histopathologically, pACTs are classified into adrenocortical adenomas (pACAs), carcinomas (pACCs), and tumors of uncertain malignant potential (pUMPs)^2^. Existing classification - including the Wieneke score, five-item score, COG staging, and the ps-GRAS score - are used to guide treatment but often fail to predict outcomes, especially in locally advanced or borderline tumors^1,3–6^.

At the genetic level, *TP53* germline variants—particularly the R337H founder mutation in Southern Brazil—are a hallmark of pACT and associated with Li-Fraumeni syndrome^7,8^. Outside of the geographical hotspot in Brazil, *TP53* germline variants occur in 50% of cases, with decreasing frequency in adolescents^9^. Somatic *TP53* mutations occur in ~25% of remaining tumors^10,11^. These findings highlight an age-dependent role of *TP53* in pACT but also indicate that additional oncogenic mechanisms contribute to disease progression. *IGF2* overexpression via 11p LOH is also common, yet insufficient to explain the broad clinical heterogeneity^11^.

pACT arise from the adrenal cortex, a highly specialized endocrine organ producing steroid hormones essential for homeostasis, development, and the stress response. During early development, the adrenal cortex arises from the adrenal-gonadal primordium and is compartmentalized into a fetal and definitive zone by 8–10 weeks of gestation, the latter giving rise to the adult cortical layers—zona glomerulosa (zG), fasciculata (zF), and reticularis (zR)^12,13^ maintained by centripetally migrating progenitor cells^14^. These processes are regulated by a complex network of molecular signals. Among these, the WNT/β-catenin pathway plays a central role in driving adrenocortical cell differentiation and maintaining tissue homeostasis throughout life. Dysregulation of WNT signaling has been implicated in adrenal hypofunction, hyperplasia, and tumorigenesis^15^.

How these developmental pathways are maintained or hijacked by oncogenic drivers in pACTs remains unclear. While TCGA studies of adult adrenocortical carcinomas have revealed key genomic and epigenetic drivers - including the CIMP phenotype and whole-genome doubling - comparable frameworks for pACTs are lacking^16^.

Previous subgrouping efforts based on *TP53* and *ATRX* mutation status^11^ or DNA methylation classes^17^ highlighted prognostic divergence but were limited by small and geographically restricted cohorts.

Here, we present an integrated molecular atlas of 214 pACTs, combining DNA methylation profiling, bulk and single-nucleus transcriptomics, chromatin accessibility, and spatial transcriptomics. This dataset addresses the clinical need for improved risk stratification and reveals a high-risk (HR) subgroup defined by CpG island hypermethylation, co-activation of WNT and AP-1 signaling, and marked microenvironmental remodeling. This HR phenotype emerges independently of *TP53* status and is marked by transcriptional plasticity and disorganized spatial architecture, suggesting a developmentally rooted, epigenetically driven basis of aggressiveness. By linking these molecular features to clinical phenotypes, we present a refined option for risk stratification and identify therapeutic strategies that may guide future trials, such as HDAC inhibition. Importantly, his work also establishes a reference resource for studying pediatric adrenal biology in health and disease.

## Results

### Cohort collection and multi-omics profiling of pediatric adrenocortical tumors

To investigate the diverse molecular landscape of pACTs, we enrolled 109 children and adolescents from the German Malignant Endocrine Tumors (MET) Registry diagnosed with a pACT between 1997 and 2024. This cohort included cases of pACCs (*n* = 60), pACAs (*n* = 35) and pUMPs (*n* = 14) according to the Wieneke criteria. Given the geographic variability with respect to tumor predisposition, we integrated previously published cohorts from Clay et al. (*n* = 60) and Bueno et al. (*n* = 57), thus including cohorts enriched for the Brazilian founder variant. Overall, this resulted in a cohort of 214 pACT and 12 healthy adrenal cortex control samples. The mean age at diagnosis across the entire cohort was 5.3 years with a female to male ratio of 2.3:1. 75.2% of patients presented with virilization (*n* = 89), Cushing’s syndrome (*n* = 20), or both (*n* = 52).

**Fig. 1:**
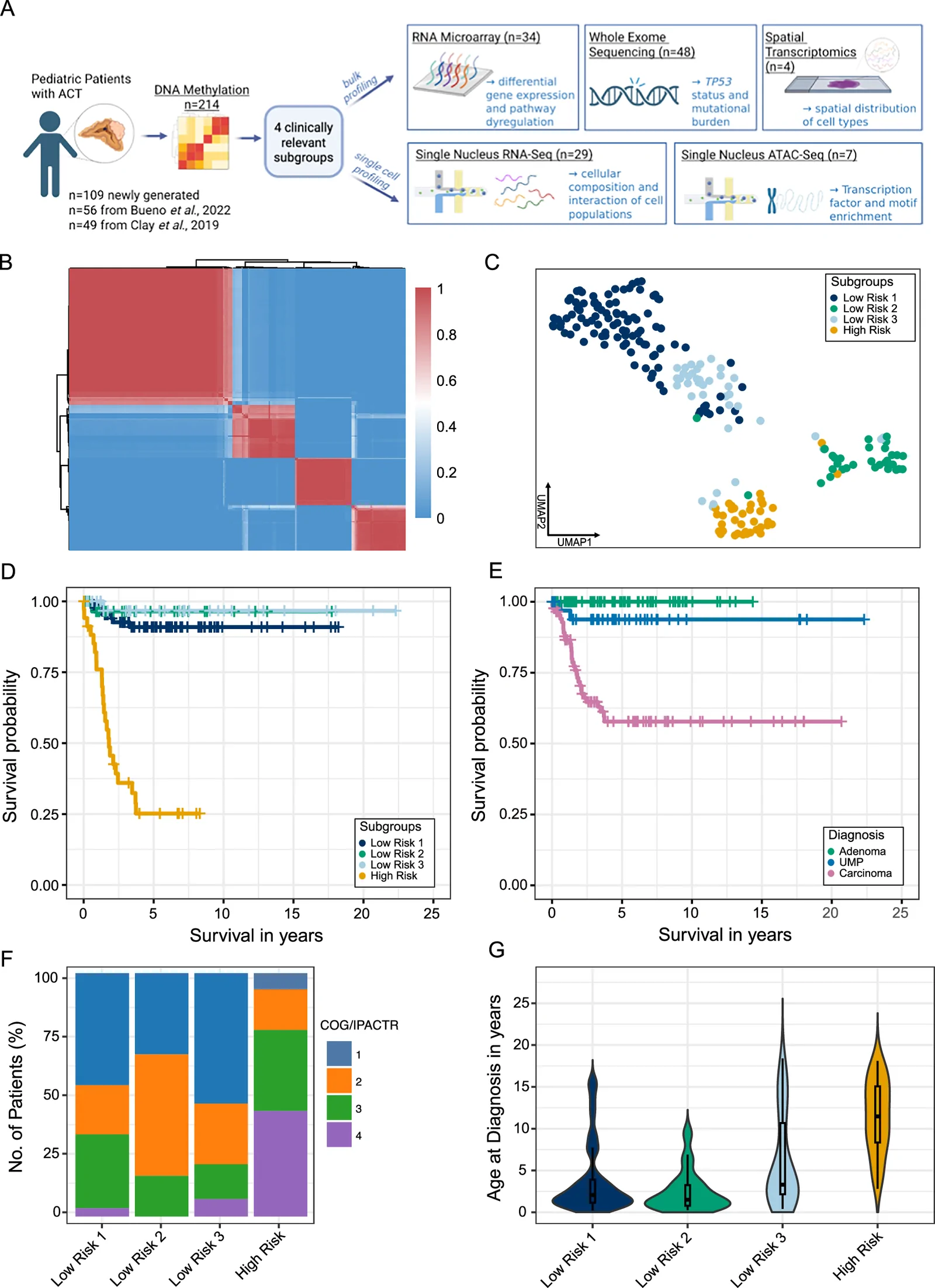
DNA methylation–based stratification of pediatric adrenocortical tumors identifies four clinically distinct subgroups. **A** Overview of the project presented in this paper. Created in BioRender. Fincke, V. (2026) https://BioRender.com/5ujxlr5. **B** Unsupervised clustering of DNA methylation data using Consnsus Clustering identifies four molecular subgroups (Low Risk 1, Low Risk 2, Low Risk 3, and High Risk) in 214 pediatric ACTs. Red indicated samples always cluster together; blue color indicates samples never cluster together (total *n* = 214; HR, *n* = 36; LR1, *n* = 104; LR2, *n* = 34; LR3, *n* = 40). **C** UMAP projection of DNA methylation profiles colored by molecular subgroup (total *n* = 214; HR, *n* = 36; LR1, *n* = 104; LR2, *n* = 34; LR3, *n* = 40). **D** Kaplan–Meier analysis of overall survival across histopathological diagnoses (pACA, *n* = 82; pACC, *n* = 92; pUMP, *n* = 40; log-rank test). **E** Kaplan–Meier analysis of overall survival across methylation-based subgroups (HR, *n* = 36; LR1, *n* = 104; LR2, *n* = 34; LR3, *n* = 40; log-rank test). **F** COG/IPACTR stage at diagnosis across methylation-based subgroups (stage 1, *n* = 75; stage 2, *n* = 67; stage 3, *n* = 27; stage 4, *n* = 36; stage not available, *n* = 9; chi-square test). **G** Age at diagnosis (years) by molecular subgroup (HR, *n* = 35; LR1, *n* = 102; LR2, *n* = 34; LR3, *n* = 40; age at diagnosis was available for 211 patients). Violin plots show the kernel density distribution of the data; embedded box plots indicate the median (center line), the 25th and 75th percentiles (box bounds), and whiskers extending to the most extreme value within 1.5 × the interquartile range of the box, which define the plotted minima and maxima. Groups were compared by two-sided Kruskal–Wallis test (P = X), followed by two-sided pairwise Dunn’s post hoc tests with Benjamini–Hochberg correction for multiple comparisons. Adjusted *P* values: LR1 vs LR2, Padj = 0.129; LR1 vs LR3, Padj = 0.018; LR1 vs HR, Padj = X; LR2 vs LR3, Padj = 0.0019; LR2 vs HR, Padj = X; LR3 vs HR, Padj = 1.64 × 10⁻⁴. Source data are provided with this paper.

Comprehensive DNA methylation profiling across the entire cohort helped define molecular subgroups and improve risk stratification in pACTs, establishing the foundation for a biologically meaningful classification. Aiming not only for an improved stratification system but also to unravel the epitranscriptomic characteristics, we enriched our dataset by single nucleus RNA-sequencing (*n *= 29) and snATACseq (*n* = 7) (Fig. 1A).

### DNA methylation profiling reveals four distinct subgroups with prognostic significance

We performed consensus clustering based on DNA methylation profiles, which identified four distinct subgroups (Fig. 1B). The robustness of this clustering was supported by clustering metrics, including the relative change in the area under the cumulative distribution function (CDF) curve at k = 4 (Supplementary Fig. S1A), confirming the stability of the four-group solution. Reduction of the high-dimensional methylation data using Uniform Manifold Approximation and Projection (UMAP) demonstrated separation of the identified subgroups (Fig. 1C). Subgroup identity was initially derived from unsupervised consensus clustering of DNA methylation profiles; risk-based designations were subsequently assigned based on the correlation of each subgroup with clinical outcome data available for our cohort. These subgroups were designated High Risk (HR), Low Risk 1 (LR1), Low Risk 2 (LR2), and Low Risk 3 (LR3), reflecting their association with patient overall survival (OS). The five-year OS for patients in the HR subgroup was 25%, which is significantly lower than for those in the Low Risk subgroups (p < 0.05; log-rank test; (HR: 25% [95% CI: 13.7%–46.5%]; LR1: 91% [95% CI: 84.7%–97.7%]; LR2: 96% [95% CI: 89.8%–100%]; LR3: 97% [95% CI: 90.5–100%])) (Fig. 1D).

Benchmarking these subgroups against the commonly used histopathological classification into pACC, pACA, and pUMP has prognostic relevance (*p* < 0.05; log-rank test (Fig. 1E)), our DNA methylation-based subgroups more accurately identified patients at high risk, indicating superior prognostic resolution.

Among the four subgroups, the largest proportion of samples was assigned to LR1 (104/214, 50.9%), followed by LR3 (40/214, 18.7%), HR (36/214, 16.8%), and LR2 (34/214, 13.6%) (Supplementary Fig. S1B). This refined classification based on DNA methylation profiles allows for improved risk stratification, particularly for pACC and pUMP patients the latter of which are diagnostically challenging. Methylation-based subgrouping showed prognostic significance in pACC, whereas no significant association with outcome was observed in the UMP and ACA diagnostic groups (Supplementary Fig. S2).

Comparison with previously established DNA methylation-based subgroup classifications by Clay et al. (A1 and A2^17^,) and Bueno et al. (pACT-1 and pACT-2^18^,) revealed partial overlap with our clustering (Supplementary Fig. S1B): To benchmark our DNA methylation–based classification against these prior subgrouping efforts, we mapped their previously defined cohorts (Clay A1/A2 and Bueno pACT-1/2) onto our risk groups. Notably, a substantial proportion of tumors previously classified as HR (Clay A1 and Bueno pACT-1) corresponded to our HR subgroup: 8 of the 10 tumors classified as pACT-1 by Bueno et al. were assigned to our HR group, with 2 classified as LR2. Similarly, of the 15 Clay A1 tumors available, 7 mapped to our HR group, while the remainder were split between LR2 (*n* = 6) and LR3 (*n* = 2) (Supplementary Data S1).

These findings demonstrate a limited intersection between previously proposed HR DNA methylation classes and our HR subgroup - particularly for the pACT-1 classification by Bueno et al. At the same time, the distribution of Clay A1 tumors across multiple subgroups in our dataset underscores the added biological granularity and refinement achieved by our four-group model: From the 8 samples that were identified as LR from the A1 group by DNA methylation, 87.5% (7 out of 8) survived the disease.

Patients in the HR subgroup presented with a more advanced tumor stage at diagnosis (Fig. 1F) and were significantly older compared to patients in the three LR subgroups (Fig. 1G). Notably, no pACA case was classified into the HR subgroup, whereas three pUMP tumors were assigned to this subgroup (Supplementary Fig. S1C). Sex distribution differed significantly across DNA methylation subgroups (χ²^3^ = 11.53, *p* < 0.05). While in the HR group sex was equally distributed, the different LR groups showed uneven female-to-male ratios (Supplementary Fig. S1D).

Pediatric adrenocortical tumors frequently present with signs of hormonal excess reflecting the steroidogenic activity of the tumor. Virilization, resulting from androgen overproduction, is the most common presentation and manifests as premature pubic hair, clitoral or penile enlargement, and accelerated growth. Cushing syndrome arises from excess cortisol secretion and is characterized by weight gain, facial rounding, hypertension, and growth retardation, while hypertension-related presentations are associated with mineralocorticoid or mixed steroid excess. The endocrine phenotype of the patients at presentation varied significantly across methylation subgroups (Fisher’s exact test with Monte Carlo simulation, p < 0.05; 100,000 replicates, Supplementary Fig. S1E). Virilization was more common in LR1, whereas Cushing syndrome was enriched in HR and rare in LR1. LR3 was associated with hypertension-related presentations. These findings support that HR tumors are more likely to present with Cushing syndrome or without endocrinological symptoms, and rarely with virilization.

The Ki-67 proliferation index is a widely used marker of tumor cell proliferation, assessed here by immunohistochemistry on tumor tissue sections, while MKI67 transcript levels were derived from bulk RNA sequencing data. Neither measure differed significantly across DNA methylation-defined subgroups (Supplementary Fig. S1F, G).

A high Ki-67 index (>20%) correlated with inferior overall survival (log-rank p < 0.05), but its prognostic performance remained modest, with time-dependent AUCs decreasing from 0.76 at 1 year to 0.65 at 10 years (Supplementary Fig. S3). In contrast, DNA methylation-based classification demonstrated superior and steadily improving prognostic accuracy, with AUCs increasing from 0.79 at 1 year after diagnosis to 0.92 at 10 years after diagnosis. While pS-GRAS retained robust prognostic capacity (Supplementary Fig. S4), it demonstrated slightly reduced long-term discriminatory power compared to DNA methylation classification, with lower AUCs at 10 years after diagnosis. While the COG-IPACTR classification, although highly predictive in the short term (AUC 0.87 at 1 year), showed a moderate decline at later time points (AUC 0.89 at 10 years). The Wieneke classification consistently underperformed relative to these models, with AUCs peaking at ~0.80 and declining to 0.72 by 10 years after diagnosis.

Importantly, the advantage of DNA methylation based subgrouping was particularly evident in clinically challenging cases classified as COG/IPACTR stage 2 or 3, i.e., larger tumors with or without local invasion whose clinical course is hard to predict, as they are non-metastatic upfront but have a tendency to recur (Supplementary Fig. 5). In this subgroup, DNA methylation achieved markedly higher discriminative power compared to both ps-GRAS and COG-IPACTR classifications, 0.93 compared to 0.86 and 0.66 at 10 years after diagnosis (Supplementary Fig. S3).

To provide molecular subgroup assignment in individual cases without requiring cohort-wide methylation clustering, we developed a random forest–based classifier trained on DNA methylation data. This tool is designed to facilitate the application of our subgroup framework in both research and clinical contexts, including settings where comprehensive reference cohorts may not be available. The classifier demonstrated high accuracy (0.934) in predicting subgroup membership, as confirmed by three nested cross-validation, yielding a high area under the receiver operating characteristic (ROC) curve (AUC) of 0.989 and a low misclassification rate of 0.066 (Supplementary Fig. S6). In the future, this online classifier can support clinical decision-making by enabling objective, molecularly informed risk stratification in diagnostic workflows and multicenter clinical trials.

### High-risk pediatric adrenocortical tumors exhibit elevated CpG island methylation and increased copy number variability

**Fig. 2:**
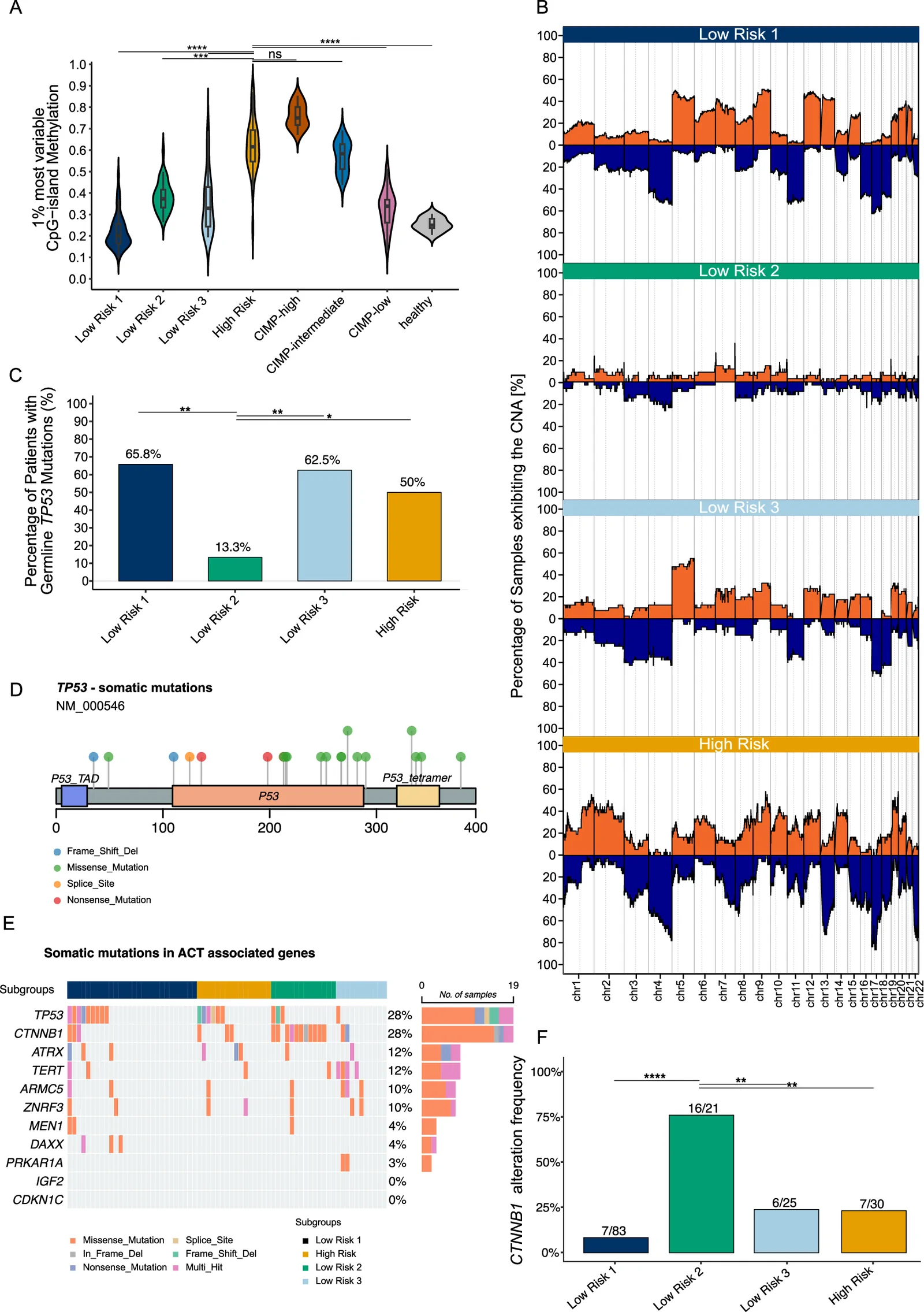
High-risk pACTs exhibit enhanced CpG island methylation, genomic instability, and enriched TP53 mutations. **A** CpG island methylation levels (top 1% most variable probes) across pACT methylation subgroups (LR1, *n* = 104; LR2, *n* = 34; LR3, *n* = 40; HR, *n* = 36), adult adrenocortical carcinoma (ACC) CpG island methylator phenotype (CIMP) categories from the TCGA cohort (CIMP-high, *n* = 20; CIMP-intermediate, *n* = 27; CIMP-low, *n* = 32) and healthy adrenal cortex controls (*n* = 12); total *n* = 305. Violin plots show the kernel density distribution of the data; embedded box plots indicate the median (center line), the 25th and 75th percentiles (box bounds) and whiskers extending to the most extreme value within 1.5 × the interquartile range of the box, which define the plotted minima and maxima. Groups were compared by two-sided Kruskal–Wallis test, followed by two-sided Dunn’s post hoc tests with Benjamini–Hochberg correction across all 28 pairwise comparisons. Source data are provided with this paper. Adjusted *p* values: LR1 vs. LR2, p.adj = 1.5 × 10⁻⁸; LR1 vs. LR3, p.adj = 4.9 × 10⁻⁶; LR1 vs. HR, p.adj = 6.7 × 10⁻²⁵; LR1 vs. CIMP-high, p.adj  = 5.0 × 10⁻²³; LR1 vs. CIMP-intermediate, p.adj = 2.8 × 10⁻¹⁸; LR1 vs. CIMP-low, p.adj = 1.2 × 10⁻⁴; LR1 vs. Healthy, p.adj = 0.263; LR2 vs. LR3, p.adj = 0.227; LR2 vs. HR, p.adj = 4.5 × 10⁻⁴; LR2 vs. CIMP-high, p.adj = 1.0 × 10⁻⁵; LR2 vs. CIMP-intermediate, p.adj = 0.0053; LR2 vs. CIMP-low, p.adj = 0.158; LR2 vs. Healthy, p.adj = 0.0201; LR3 vs. HR, p.adj = 1.0 × 10⁻⁶; LR3 vs. CIMP-high, p.adj = 2.5 × 10⁻⁸; LR3 vs. CIMP-intermediate, p.adj = 4.5 × 10⁻⁵; LR3 vs. CIMP-low, p.adj = 0.734; LR3 vs. Healthy, p.adj = 0.142; HR vs. CIMP-high, p.adj = 0.158; HR vs. CIMP-intermediate, p.adj = 0.672; HR vs. CIMP-low, p.adj = 9.8 × 10⁻⁷; HR vs. Healthy, p.adj = 1.0 × 10⁻⁶; CIMP-high vs. CIMP-intermediate, p.adj = 0.0949; CIMP-high vs. CIMP-low, p.adj = 2.5 × 10⁻⁸; CIMP-high vs. Healthy, p.adj = 2.5 × 10⁻⁸; CIMP-intermediate vs. CIMP-low, p.adj = 3.0 × 10⁻⁵; CIMP-intermediate vs. Healthy, p.adj = 1.1 × 10⁻⁵; CIMP-low vs. Healthy, p.adj = 0.214. *p.adj <0.05; **p.adj <0.01; ***p.adj <0.001; ****p.adj <0.0001; ns, not significant. Source data are provided as a Source Data file. **B** Copy number alteration frequency across chromosomes stratified by methylation subgroup. Gains and losses are shown as the percentage of samples affected per chromosome (total *n* = 214, HR, *n* = 36; LR1, *n* = 104; LR2, *n* = 34; LR3, *n* = 40). **C** Frequency of germline TP53 mutations across methylation-based subgroups (HR, *n* = 26; LR1, *n* = 76; LR2, *n* = 15; LR3, *n* = 24)^17,18^. Pairwise two-sided Fisher’s exact tests with Benjamini-Hochberg correction for multiple comparisons: LR1 vs. LR2, p.adj = 0.00176; LR2 vs. LR3, p.adj = 0.00996; LR2 vs. HR, p.adj = 0.0462; LR1 vs. LR3, p.adj = 0.809 (ns); LR1 vs. HR, p.adj = 0.252 (ns); LR3 vs. HR, p.adj = 0.488 (ns). * p.adj <0.05, ** p.adj <0.005. Percentages indicate subgroup-specific germline TP53 mutation rates. Source data are provided as a Source Data file. **D** Distribution and types of somatic TP53 mutations across functional domains of the TP53 gene. **E** Frequency of somatic mutations in known ACT-associated genes (e.g., TP53, CTNNB1, IGF2, ATRX) among patients with available whole-exome sequencing (*n* = 45: HR, *n* = 11; LR1, *n* = 22; LR2, *n* = 8; LR3, *n* = 4). **F** Bar plot displaying the frequency of somatic CTNNB1 mutations per subgroup (*n* = 159: HR, *n* = 30; LR1, *n* = 83; LR2, *n* = 21; LR3, *n* = 25); two-sided pairwise Fisher’s exact tests, Benjamini-Hochberg corrected. Adjusted *p*-values: HR vs. LR1, p.adj = 0.0754 (ns); HR vs. LR2, p.adj = 0.00135 (**); HR vs. LR3, p.adj = 1.000 (ns); LR1 vs. LR2, p.adj <0.0001 (****); LR1 vs. LR3, p.adj = 0.0863 (ns); LR2 vs. LR3, p.adj = 0.00179 (**). ns, not significant (**p.adj <0.01, ****p.adj <0.0001). Source data are provided as a Source Data file.

Analysis of methylation levels of the subgroups in comparison to 12 healthy adrenal cortex samples^17^ and 79 adult ACTs^16^ revealed CpG island hypermethylation in HR pACTs with a median methylation level of 0.62 compared to 0.2 in LR1 (Fig. 2A). Methylation levels differ significantly among the HR subgroup and all LR subgroups (Dunn’s test, *p* < 0.05), but not when comparing the pACT HR to the adult CIMP-high subgroup (median methylation level = 0.75). In UMAP projection the HR subgroup exhibits notable similarity to the adult CIMP-High subgroup (Supplementary Fig. S7A, B).

Methylation-derived copy number analysis revealed distinct subgroup-specific patterns of chromosomal instability (Fig. 2B). HR tumors exhibited a high burden of recurrent chromosomal alterations, as determined by GISTIC analysis. Specifically, we identified six significant copy number gains and twenty losses. Amplified regions included oncogenic loci such as *MYC*, *CDK4*, *MDM2*, and *AURKC*, while recurrent deletions encompassed key tumor suppressor genes including *BRCA2*, *CHEK2* and *TP73*. However, expression levels of these genes showed highly heterogeneous patterns across HR patients and did not consistently correlate with the underlying copy number status, pointing to high inter-patient variability and suggesting that additional regulatory mechanisms beyond copy number may govern their expression in this subgroup (Supplementary Fig. S8A). In contrast, LR2 tumors showed remarkable genomic stability, with only a single recurrent deletion on chromosome 4q35.2 (Supplementary Fig. S8B-E).

Subgroup-specific differences in genetic alterations were evident: LR2 tumors exhibited a significantly lower frequency of germline *TP53* variants (13.3%) than each of the other three subgroups, whereas frequencies in HR, LR1, and LR3 ranged from 50.0% to 65.8% and did not differ significantly from one another (pairwise two-sided Fisher’s exact tests with Benjamini-Hochberg correction for multiple comparisons; LR2 vs. LR1, p.adj = 0.00175; LR2 vs. LR3, p.adj = 0.00995; LR2 vs. HR, p.adj = 0.0462; all other pairwise comparisons ns; Fig. 2C). At the somatic level, *TP53* mutations were more evenly distributed across subgroups and were also present in LR2 (13.3%), though at lower frequency than in HR (23.1%). These somatic alterations spanned key functional domains of TP53 - including the DNA-binding and tetramerization regions - and included missense, frameshift, and splice-site variants (Fig. 2D).

Further somatic mutational analysis revealed *TP53* and *CTNNB1* (28%, respectively) as the most frequently altered genes, with infrequent mutations in *ATRX, DAXX, TERT*, and *IGF2* (Fig. 2E, Supplementary Fig. S9, Supplementary Data S2). Striking differences in the distribution of somatic *CTNNB1* mutations were observed across subgroups, with LR2 exhibiting a significantly elevated mutation frequency (76.2%) compared to all other subgroups (5.1–24%; p < 0.5) (Fig. 2F). Expression of genes known to be mutated in adult ACCs, such as *ATRX, DAXX* or *TERT*, do not show any differences in RNA expression between subgroups (Supplementary Fig. S8).

### The composition of pediatric adrenocortical tumors mimic the physiological, zonal differentiation of the physiological adrenal cortex

**Fig. 3:**
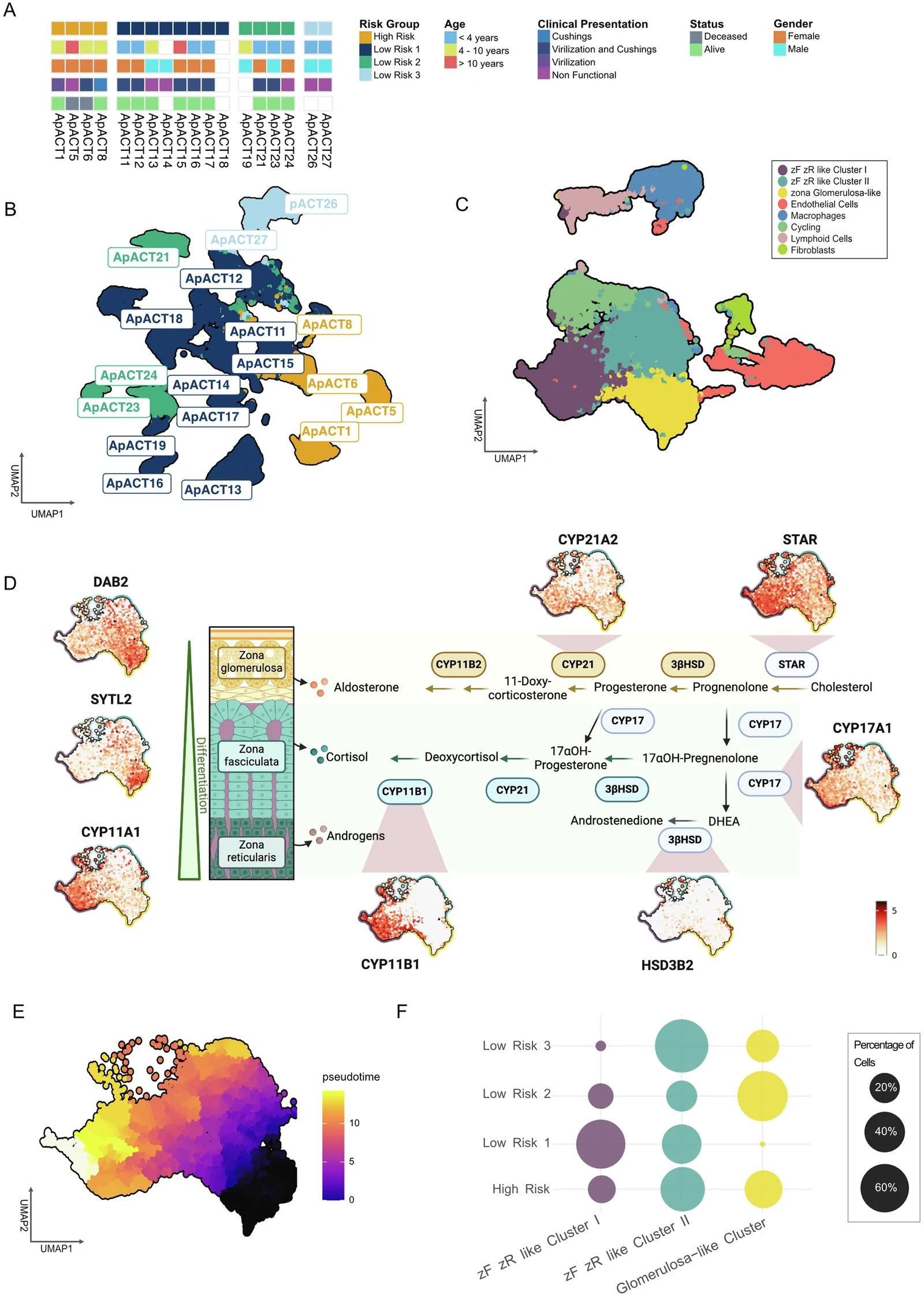
Single-Nucleus RNA sequencing reveals adrenal zonation and cell type diversity across pACT zubgroups. **A** Clinical annotation of each sample, including risk group, gender, age category, clinical presentation, and survival status. **B** UMAP projection of snRNA-seq data from 18 pACT samples ((HR: 4 pACC; LR1: 4 pACC, 4 pACA; LR2: 1 pACC, 2 pACA, 1 pACx; LR3: 2 pACC)), colored by patient ID for *n* = 75,515 nuclei. **C** UMAP embedding of all cells (*n* = 75,515 cells, *n* = 18 samples) colored by inferred cell type identity, including glomerulosa-like, zF/zR-like Cluster I and II, macrophages, lymphoid cells, endothelial cells, fibroblasts, and cycling cells. **D** Expression of key adrenal steroidogenic genes across the UMAP space. Genes shown include *DAB2*, *SYTL2*, *CYP11A1*, *CYP21A2*, *STAR*, *CYP17A1*, *CYP11B1*, and *HSD3B2*. Diagram (center) summarizes the biosynthetic zonation of the adrenal cortex and corresponding gene functions. Created in BioRender. Fincke, V. (2026) https://BioRender.com/34flu07. **E** Pseudotime projection of tumor cells (*n* = 53,943 tumor cells, *n* = 18 samples), indicating a potential differentiation trajectory from glomerulosa-like cells toward zF/zR-like clusters. **F** Proportional representation of tumor cell clusters across molecular subgroups (*n* = 18 samples divided across *n* = 4 subgroups: HR *n* = 4, LR1 *n* = 8, LR2 *n* = 4, LR3 *n* = 2). Bubble size reflects the relative abundance of each cluster per subgroup.

As the genomic differences do not account for all the biological differences among the subgroups, we hypothesized that cellular composition among the subgroups differs substantially. We therefore performed single-nucleus RNA sequencing (snRNA-Seq) on 18 pACT samples representing all DNA methylation-based risk groups including 11 pACCs, 6 pACAs, and 1 pUMP (Fig. 3A, B). Notably, samples of the same subgroup clustered together at this data level. Following quality control measures, transcriptional profiles from 75,515 single cells, with a median of 1,297 genes per cell, were retained for subsequent analysis. Using established markers for cells of the tumor microenvironment (TME)—including *CD68* and *CD163* for macrophages, *ACTA2* and *PDGFRB* for fibroblasts, *CD3D* and *CD79A* for lymphoid cells, and *PECAM1* for endothelial cells—distinct clusters of these populations were identified, alongside multiple tumor cell clusters (Fig. 3C, Supplementary Data S3).

To characterize the TME across risk groups, we systematically quantified the proportional abundance of immune and stromal cell populations in each sample (Supplementary Fig. S10). This analysis revealed significant differences in macrophage and lymphoid cell proportions across methylation-defined risk groups (Kruskal-Wallis, *p* = 0.047 and *p* = 0.049, respectively). HR tumors showed a marked depletion of macrophages compared to LR1 and LR2 tumors (median 1.5% vs. 8.1% and 10.3%, respectively), and were similarly depleted of lymphoid cells (median 0.2% in HR vs. 4.2% in LR1). Endothelial cell and fibroblast proportions did not differ significantly across subgroups (*p* = 0.329 and *p* = 0.265, respectively), although endothelial cells were numerically lower in HR tumors (median 3.1%) compared to LR1 (median 16.4%). Collectively, these findings suggest that HR pACTs are characterized by a markedly immunosilenced microenvironment, with reduced macrophage infiltration and lymphoid cell abundance, which may contribute to immune evasion and the more aggressive clinical behavior of this subgroup.

Analysis of the tumor subclusters revealed that their transcriptional profiles closely resembled those of the physiological adrenal cortex, as evidenced by the expression of zone-specific marker genes. Two clusters clearly displayed features resembling the zF and zR, distinguished by the expression of *CYP17A1* and *CYP11A1*, respectively - key steroidogenic enzymes that serve as established markers for these adrenal cortical zones (Fig. 3D). Another tumor cluster exhibited high expression of genes typically upregulated in the outermost adrenal cortex zone, zG, including *CYP11B2* and *DAB2*. This cluster was therefore classified as the zG-like cluster. Additionally, a distinct cluster of cycling tumor cells was identified, characterized by elevated S.Score and G2M.Score in cell cycle analysis (Supplementary Data S4). Thus, the transcriptome of pACTs parallels the physiological cell types present in the adrenocortex.

This observation is further corroborated by pseudotime analysis suggesting a differentiation trajectory, originating from zG-like cells and progressing toward the zF/zR-like clusters, thereby mimicking the differentiation pattern of the healthy adrenal cortex (Fig. 3E). To investigate how this transcriptional hierarchy varies across molecular subgroups, we quantified the representation of each transcriptional cell state within the DNA methylation-based subgroups (Fig. 3F). We found that the HR subgroup was characterized by a nearly balanced distribution of cells in the zG-, zF/zR-like states in contrast to the more skewed cell state distributions observed in the LR subgroups. These relative enrichment and co-existence of all three major adrenal cortical-like lineages in HR tumors suggest a broader differentiation spectrum and increased plasticity within this group and suggests that each of these cell populations may fulfill a distinct role in tumorigenesis.

Strikingly, HR tumors exhibited the highest cellular heterogeneity, with an average Shannon Index of 1.01, compared to values ranging from 0.61 to 0.87 in the other subgroups. This elevated diversity supports the notion that HR tumors are not only transcriptionally distinct but also compositionally more complex than the LR groups.

Because bulk DNA methylation profiling was performed on heterogeneous tumor samples, we next asked whether subgroup-specific methylation differences might reflect variation in cellular composition rather than tumor-intrinsic epigenetic states. To address this, we integrated bulk methylation data with matched snRNA-seq profiles. Global methylation levels were not associated with tumor cell fraction estimated from snRNA-seq (Spearman ρ = 0.06, p = 0.82), nor with tumor pseudobulk transcriptional activity (ρ = 0.02, p = 0.95). In addition, no significant correlations were observed between global methylation and the proportional abundance of individual tumor or microenvironmental clusters (Supplementary Fig. S11). These findings suggest that methylation differences between molecular subgroups are unlikely to be driven by compositional effects and instead reflect tumor-intrinsic epigenetic programs.

This prompted us to investigate the regulatory models which underlie the different subgroups by using NMF based decomposition.

### Canonical WNT-signaling, emanated by the ZG-like population, represents a major constituent of the HR subgroup

**Fig. 4:**
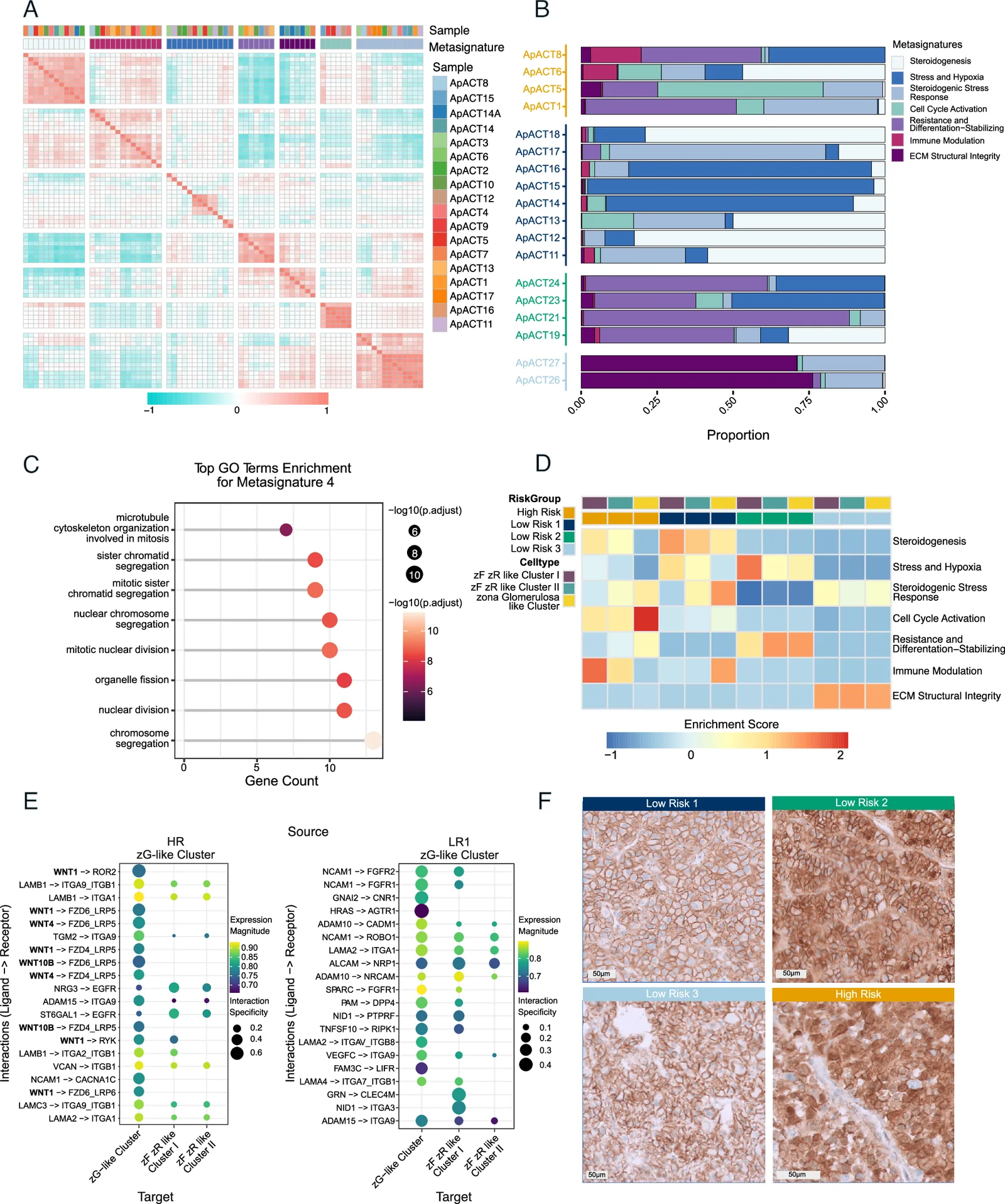
Transcriptional metasignatures reveal functional heterogeneity and differential WNT pathway engagement across pACT Subgroups. **A** Heatmap of pairwise correlations among seven transcriptional metasignatures derived from non-cycling tumor cells using non-negative matrix factorization (NMF) across 18 pACT samples. **B** Bar plots showing sample-level proportions of metasignature expression. Each bar represents one tumor sample, colored by metasignature contribution. **C** GO term enrichment analysis for Metasignature 4 (*n* = 30 genes), annotated as “Proliferative and Cell Cycle Activation,” showing top enriched pathways and gene counts. Enrichment was tested against the default genome-wide background of clusterProfiler using a one-sided hypergeometric test (clusterProfiler enrichGO), with *p* values adjusted for multiple comparisons by the Benjamini-Hochberg method. **D** Enrichment heatmap showing the prevalence of metasignatures in the tumor cell clusters in the molecular risk groups(HR: 4 pACC; LR1: 4 pACC, 4 pACA; LR2: 1 pACC, 2 pACA, 1 pACx; LR3: 2 pACC). **E** Dot plot showing ligand–receptor interactions inferred by LIANA across tumor clusters (zF/zR-like Cluster I *n* = 17,970 cells, zF/zR-like Cluster II *n* = 22,619, zG-like Cluster *n* = 12,354 cells. Zona fasciculata (zF), zona reticularis (zR), zona glomerulosa (zG). **F** Immunohistochemical staining for CTNNB1 in representative tumor samples from each risk group (HR, *n* = 7; LR1, *n* = 4; LR2, *n* = 3; LR3, *n* = 3 tumors stained). High Risk and Low Risk 2 tumors show strong nuclear localization, while Low Risk 1 and Low Risk 3 exhibit primarily membranous staining, indicating subgroup-specific activation of canonical WNT signaling. The images shown are representative of the staining pattern observed in all stained samples of the respective subgroup. Scale bars indicate 50 µm.

NMF based decomposition enables the deconvolution of complex gene expression matrices into interpretable components, each representing a distinct transcriptional program active across a subset of cells, thereby revealing patterns that are not necessarily aligned with predefined cell clusters or known lineages. NMF was applied independently to each sample, which revealed seven shared transcriptional metasignatures after hierarchical clustering (Fig. 4A, Supplementary Data S5). These metasignatures (MS) were differentially distributed among samples across the DNA methylation-based risk groups (Fig. 4B). To characterize each metasignature, we performed Gene Ontology (GO) enrichment analysis and examined the top overexpressed genes. MS4 (Cell Cycle Activation) showed strong enrichment for mitotic processes (e.g., chromosome segregation, nuclear division) and was defined by genes such as *NUF2, FAM166B*, and *LSS*, indicating a highly proliferative cell state (Fig. 4C). In contrast, MS1 (Steroidogenesis) included canonical steroidogenic markers such as *SCARB1, CYP11B1*, and *STAR*. MS2 and MS3 captured distinct transcriptional stress programs: MS2 (Stress and Hypoxia) was enriched for corresponding genes (e.g., *HIF3A, PLAGL1, FAM166B*), consistent with adaptation to a nutrient-deprived or hypoxic microenvironment. In contrast, MS3 (Steroidogenic Stress Response) reflected a steroidogenic stress response, marked by upregulation of *STAR, FKBP5, SULT2A1*, and *HSP90AA1*, suggesting sustained hormonal output and compensatory mechanisms supporting tumor cell survival under chronic steroidogenic pressure. MS5 (Resistance and Differentiation-Stabilizing) included *COL11A1* and *RAB38*, which have been implicated in extracellular matrix remodeling and therapy resistance mechanisms. Further, MS6 (Immune Modulation) was defined by genes such as *ZNF117* and *CHST15*, while MS7 (ECM Structural Integrity) involved *ERV3-1, GRIN2C*, and *ZNF431*, collectively suggesting roles in tumor–immune interactions and stromal architecture.

Importantly, the distribution of these metasignatures also reflected the clinical presentation associated with each methylation subgroup (Supplementary Fig. S1E). For instance, LR3 tumors—enriched for MS1 (Steroidogenesis) and MS3 (Steroidogenic Stress Response)—showed a significant overrepresentation of virilization symptoms, consistent with enhanced or dysregulated androgen biosynthesis. Conversely, HR tumors, characterized by MS4 (Cell Cycle Activation) and MS6 (Immune Modulation), were associated with Cushing’s syndrome, aligning with the proliferative and cortisol-producing profile of these tumors. Finally, LR3 tumors—clinically marked by frequent virilization—were enriched for MS1 (Steroidogenesis) and MS5 Resistance and Differentiation Stabilizing, suggesting a more differentiated and hormonally specialized transcriptional state.

The metasignatures exhibited differential distribution not only within risk groups but also between tumor cell populations. Notably, the MS4 (Cell Cycle Activation) was strongly—and almost exclusively—present in the zG-like cluster within the HR subgroup (Fig. 4D). While LR2 and LR3 tumors were predominantly defined by a limited number of transcriptional programs, LR1 and HR tumors exhibited broader metasignature diversity. In the HR subgroup—which exhibited a balanced distribution of all major transcriptional clusters—the concurrent presence of diverse metasignatures suggests that inter-cluster communication may play a critical role in driving tumor progression.

**Fig. 5:**
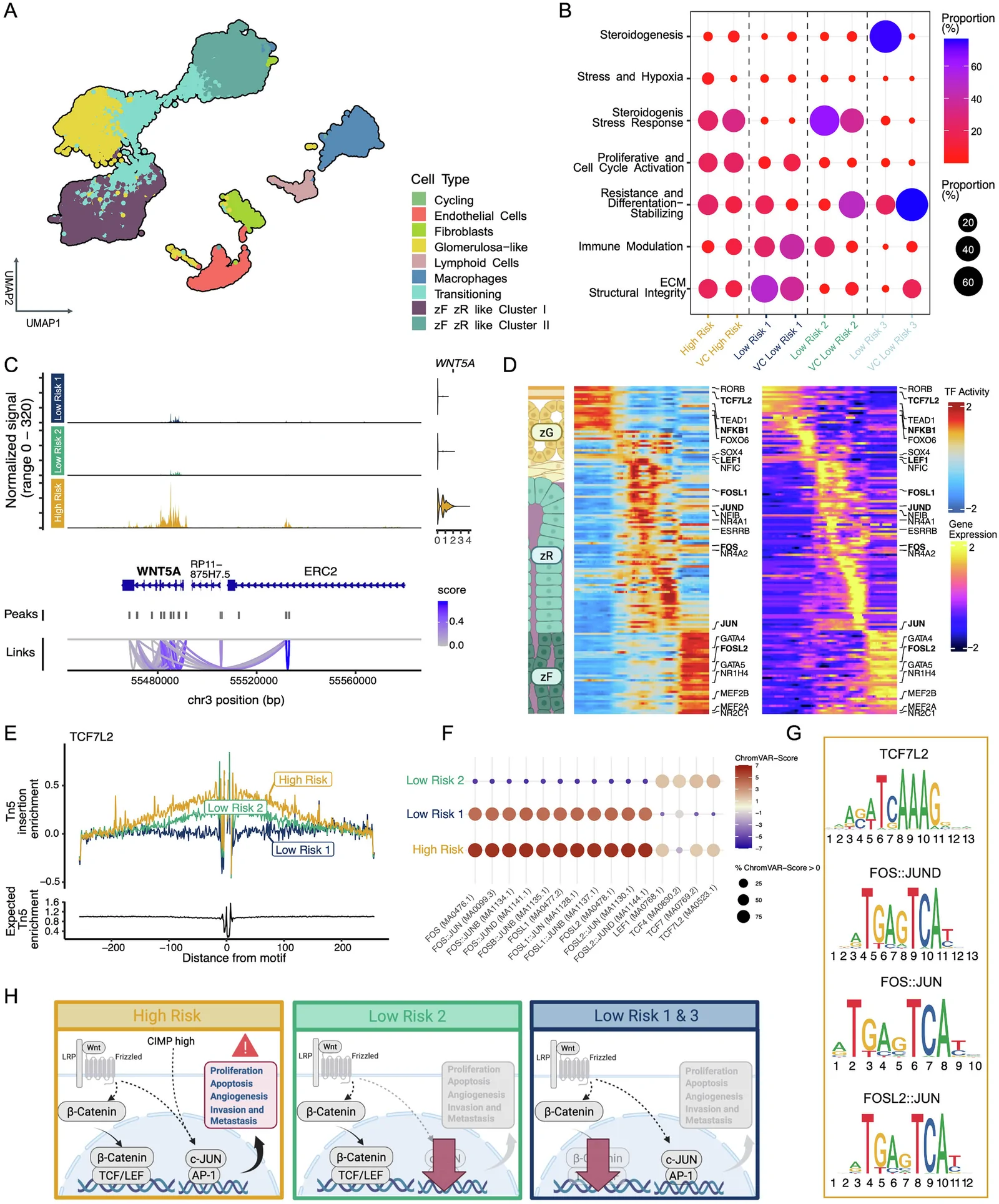
Chromatin accessibility and transcription factor motif enrichment define subgroup-specific regulatory programs in pediatric ACT. **A** UMAP embedding of snRNA-Seq data from the validation cohort (*n* = 10 pACT samples, *n* = 50,988 nuclei), confirming cell type annotations observed in the primary cohort. Transitional cluster exhibiting features of both zG- and zF/zR-like tumor cells is highlighted. **B** Proportional distribution of metasignatures across DNA methylation–based risk groups in the original and validation cohort (VC). **C** Chromatin accessibility tracks from snATAC-Seq data showing elevated accessibility at the WNT5A locus (*n* = 7). **D** Pseudotime trajectory of tumor cell differentiation inferred from snRNA-Seq and snATAC-Seq integration, showing dynamic expression (right) and activity (left) of selected transcription factors, including TCF7L2 and members of the AP-1 complex (FOS, JUN, JUND) (*n* = 7). Created in BioRender. Fincke, V. (2026) https://BioRender.com/5kt1mj1. **E** Motif enrichment analysis based on Tn5 insertion frequency around TCF7L2 binding motifs. High Risk tumors exhibit the strongest enrichment at this CTNNB1-binding motif (*n* = 7). Equivalent figure for AP-1 is displayed in Supplementary Fig. S15D. **F** ChromVar scores for CTNNB1-binding and AP-1 binding motifs across risk groups (*n* = 7). Source data are provided as a Source Data file.**G** Top enriched transcription factor motifs in High Risk tumors derived from snATAC-Seq, showing selective cso-enrichment of WNT/TCF and AP-1 binding sites (*n* = 7). **H** Overview of the mechnismns potentially involved in driving malignancy of the pACT High Risk subgroup. Created in BioRender. Fincke, V. (2026) https://BioRender.com/e38pwvx.

To dissect the intercellular communication dynamics of zG-like tumor cells in different risk subgroups, we performed ligand-receptor interaction analysis stratified by subgroups. In HR tumors, zG-like cells exhibited a prominent WNT signaling axis, characterized by extensive ligand-receptor interactions including *WNT1, WNT4*, and *WNT10B*, primarily targeting *FZD4_LRP5* and *FZD6_LRP5* complexes in both intra- and intercluster communication (Fig. 5E). These interactions displayed high expression magnitude (0.85–0.9) and interaction specificity. Additionally, ECM-related interactions (e.g., *LAMB1-ITGA9/ITGB1, VCAN-ITGB1*) and EGFR pathway ligands (*NRG3, ST6GAL1*) further enriched the signaling landscape, suggesting a pro-proliferative and migratory signaling environment. In contrast, zG-like cells in LR1 tumors showed a distinct signaling profile, largely devoid of canonical WNT ligands. Instead, interactions were enriched for cell adhesion and developmental pathways, including *NCAM1-FGFR2*, *HRAS-AGTR1*, and *LAMA2-ITGA1* (Supplementary Fig. S10, Supplementary Data S6). These interactions point toward a more differentiated and structurally organized state, with lower interaction specificity and more limited signaling breadth compared to HR tumors. Notably, the absence of canonical WNT ligand engagement in LR1, LR2, and LR3 further highlights the molecular divergence between the subgroups.

Given the prominent role of WNT signaling, we evaluated CTNNB1 localization by immunohistochemistry as a surrogate marker of pathway activation. HR and LR2 tumors exhibited nuclear accumulation of CTNNB1, whereas LR1 and LR3 tumors showed exclusively membranous staining, supporting subgroup-specific differences in WNT activation (Fig. 4F). All samples in the LR2 and HR subgroup with somatic *CTNNB1* mutations exhibited nuclear localization of CTNNB1 (5/5, 100%). Notably, however, 71.5% (5/7) HR tumors without *CTNNB1* mutations also showed robust nuclear accumulation, suggesting that WNT pathway activation in this subgroup occurs through alternative mechanisms. Despite this shared feature between HR and LR2, their divergent clinical outcomes indicate that WNT activation alone is insufficient to explain aggressiveness, and that additional cooperating factors likely contribute to the malignant phenotype in HR tumors.

### Cell type and metasignature distributions remain consistent in an independent validation cohort, demonstrating reproducibility

To validate the identified cell types in our tumor samples, we performed additional snRNA-seq (*n *= 3) and combined single-nucleus RNA and ATAC sequencing (snMultiome, *n* = 7) on additional cases. Filtering and cell-type annotation were performed following the same protocol as in the primary cohort, resulting in 50,799 cells with an average of 2252 detected genes per cell. The identified cell types in the validation cohort closely mirrored those from the original dataset, confirming the reproducibility of our cell type annotations. Notably, we identified a “Transitioning” cluster—exhibiting transcriptional characteristics intermediate between zG cells and zF/zR-like tumor populations - which was predominantly enriched in HR tumors (20.1% in the validation cohort; 0.6–9% in LR groups, Fig. 5A). Importantly, the proportional distributions of tumor and microenvironmental cell types were remarkably consistent between the cohorts. For example, zG-like tumor cells were most abundant in LR1 (36.6% in the original cohort; 28.9% in the validation cohort), while zF/zR-like Cluster II dominated LR3 (61.1% and 85.0%, respectively). These concordant patterns underscore the robustness of our integrated classification and the biological reproducibility of the subgroup-specific cellular architecture (Supplementary Fig. S13, Supplementary Data S7). Previously defined metasignatures were found again and analyzed across subgroups. As shown in Fig. 5B, HR tumors exhibited a highly heterogeneous composition, with all seven metasignatures present and none exceeding ~32% of the transcriptional profile, whereas LR tumors were dominated by a single metasignature.

To further investigate the biological divergence between LR2 and HR tumors—aspects not explained by WNT signaling alone, which is upregulated in both groups—we performed an integrated single-cell multiomic analysis, combining snRNA-seq and snATAC-seq data. Joint UMAP-based dimensionality reduction confirmed consistent separation of the subgroups across transcriptomic, chromatin accessibility, and weighted nearest neighbor (WNN) modalities (Supplementary Fig. S14).

### Regulatory motif analysis suggests CTNNB1- and AP1-binding motifs as drivers of tumor biology in high risk pACTs

To further explore the regulatory underpinnings of subgroup-specific biology, we leveraged the snATAC-seq data generated as part of the reproducibility analysis described above. Given that the genetic landscape of the subgroups—with only two samples in the HR group displaying *CTNNB1* mutations—does not provide a clear explanation for the overrepresentation of the WNT pathway in the HR group, we hypothesized that epigenetic changes may be responsible for this characteristic. SnATAC-seq revealed increased accessibility at regulatory regions of key WNT pathway genes, including e.g. *WNT5A* particularly in HR tumors (Fig. 5C). Pseudotime analysis reconstructed a trajectory starting from zG-like tumor cells toward more differentiated zF/zR-like populations (Supplementary Fig. S15A, B). Of note, key effectors of the WNT pathway such as TCF7L2 or LEF were overrepresented early in pseudotime which is consistent with the notion that WNT signaling is a property exerted mainly by un differentiated zG-like cells in the HR group. A further salient pattern in the pseudotime analysis was the overrepresentation of AP-1 transcription factor complex components such as FOS, JUN, and JUND which peaked later in the trajectory (Fig. 5D; Supplementary Fig. S15C).

A detailed motif enrichment analysis of TCF7L2 and FOS::JUND binding sites revealed a striking gradient in chromatin accessibility across risk groups: HR tumors showed the strongest enrichment at FOS::JUND motifs, followed by LR2, whereas LR1 exhibited minimal accessibility at these sites (Supplementary Fig. S15D). Notably, no significant difference in AP-1 binding motif accessibility was observed between HR and LR1, consistent with both groups displaying AP-1 regulatory activity; however, HR tumors uniquely co-activate WNT signaling alongside AP-1 programs, as reflected by concurrent TCF7L2 motif enrichment, whereas LR1 activity appears restricted to AP-1 alone. This accessibility gradient closely parallels the transcriptional activity of AP-1 components observed in snRNA-seq, supporting a model in which HR tumors—unlike the other LR groups—co-activate WNT and AP-1 regulatory programs.

HR tumors demonstrated markedly increased chromatin accessibility and transcription factor (TF) activity involving both TCF7L2 and AP-1 family members, whereas LR subgroups (LR1 and LR2) exhibited selective enrichment for only one TF class, respectively (Fig. 5F). Consistent with this, ChromVAR analysis revealed substantially elevated motif deviation scores in HR tumors compared to LR counterparts, particularly for AP-1 complexes, including FOS::JUND and FOS::JUN Increased TCF7L2 activity was likewise observed in HR tumors, highlighting a distinct transcriptional regulatory program associated with aggressive tumor biology (Fig. 5G). In contrast, LR2 tumors lacked AP-1 motif enrichment, and LR1 tumors showed no evidence of WNT pathway activation, further corroborated by the absence of nuclear CTNNB1 localization (Fig. 5H). Collectively, these data point toward transcriptional cooperativity between WNT and AP-1 effectors as a defining regulatory hallmark of HR pACT, potentially underpinning its more aggressive clinical behavior.

### High resolution spatial transcriptomics analysis revealed diverse cell type composition in high risk tumors

**Fig. 6:**
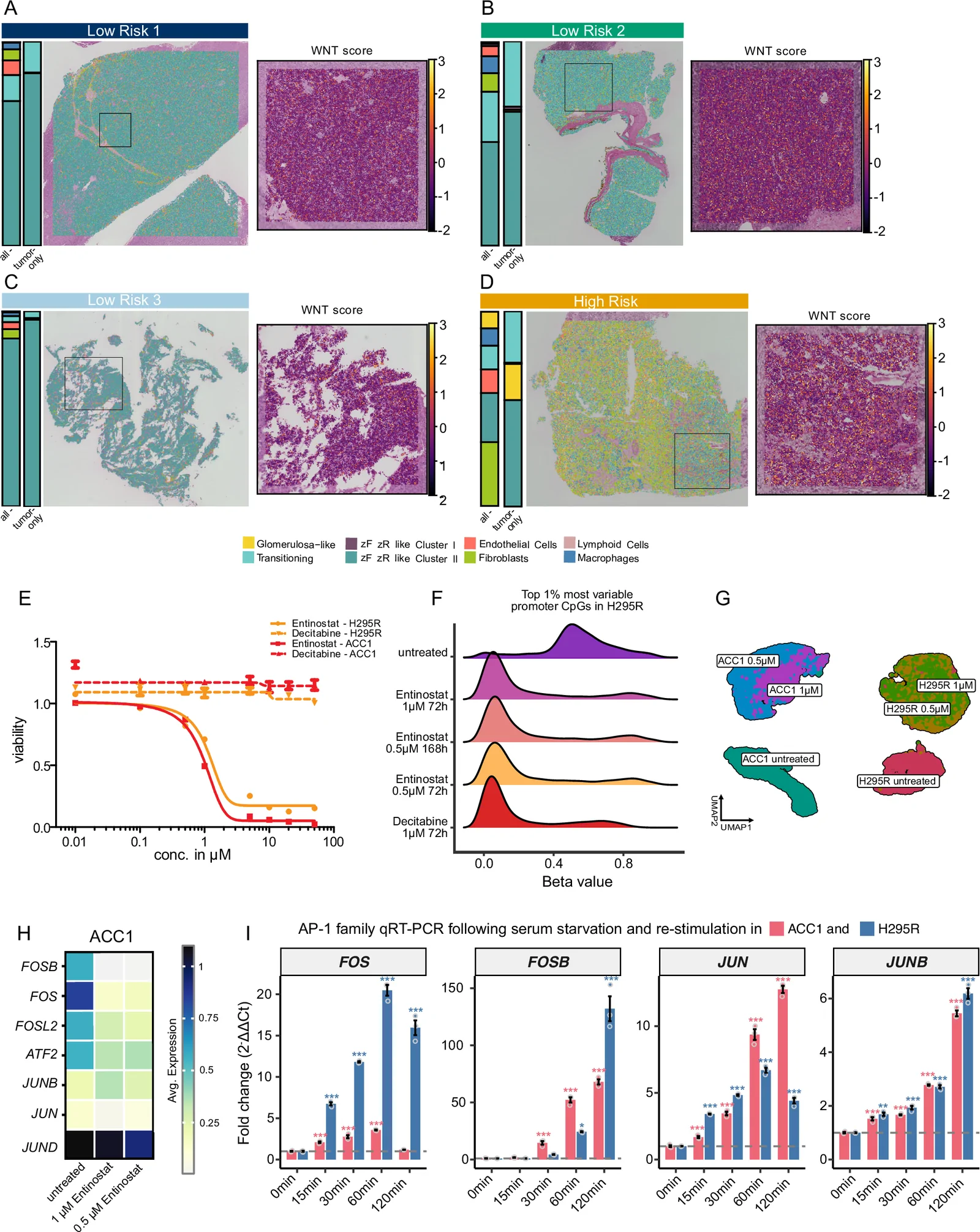
Spatial and epigenetic reprogramming define high-risk architecture and therapeutic vulnerabilities in pACT and ACC models. Spatial transcriptomic analysis of one representative tumor per DNA methylation-based subgroup using the Visium HD platform: **A** Low Risk 1, **B** Low Risk 2, **C** Low Risk 3, D High Risk. Cells were reconstructed from 2 µm bins with bin2cell and annotated by transferring the single-nucleus RNA-seq cluster labels with CellTypist. Cells analysed: Low Risk 1, *n* = 289,651 (tumor compartment 243,148, 83.9%); Low Risk 2, *n* = 140,105 (108,281, 77.3%); Low Risk 3, *n* = 99,273 (89,028, 89.7%); High Risk, *n* = 146,167 (67,396, 46.1%). The stacked bars show the cell type composition across all reconstructed cells (“all”) and across the tumor compartment only (“tumor only”). The Low Risk tumors (**A**–**C**) display a compartmentalised architecture, whereas the High Risk tumor (**D**) shows a disordered structure with an expanded stromal compartment. Insets show PROGENy-inferred WNT signaling activity for the boxed region on an identical color scale across all panels (−2 to 3), comprising 26,579 (**A**), 30,432 (**B**), 17,483 (**C**) and 19,975 (**D**) cells, respectively; the highest WNT activity was observed in the High Risk section. One section was analysed per subgroup, so no statistical comparison between subgroups was performed and the differences shown are descriptive. **E** Dose–response viability assays of H295R and CU-ACC1 cells treated with entinostat for 72 h at increasing concentrations. Data are presented as mean values ± SEM (*n* = 3 independent biological replicates per cell line). **F** Global methylation changes at the top 1% most variable promoter-associated CpG sites following Entinostat treatment of H295R, showing hypomethylation across conditions (*n* = 1000 CpG-sites per condition). **G** UMAP projection of scRNA-seq profiles from treated and untreated ACC cells (H295R and ACC1), demonstrating distinct clustering and transcriptional reprogramming upon HDAC inhibition. (H) Expression heatmap of AP-1 transcription factor components (*FOS, FOSB, FOSL2, JUN, JUND, JUNB, ATF2*) in treated versus untreated ACC cells, showing consistent downregulation after Entinostat exposure. (I) Bar plots show mean fold change (FC = 2⁻ΔΔCt) in mRNA expression of *FOS*, *FOSB*, *JUN*, and *JUNB* relative to the 0 min timepoint, in H295R (blue) and ACC1 (red) cells at the indicated timepoints after induction. Individual data points represent biologically independent replicates (*n* = 3 per group; open circles). Error bars denote ± standard error of the mean (SEM). The dashed horizontal line indicates FC = 1 (no change). Statistical comparisons were performed using one-way ANOVA followed by Tukey’s honestly significant difference (HSD) post-hoc test, comparing each timepoint to the 0 min baseline within each cell line; this test is two-sided and inherently adjusts for multiple comparisons. Asterisks denote Tukey-adjusted *p* values (*p < 0.05, **p < 0.01, ***p < 0.001); bars not marked with an asterisk were not statistically significant. Values, exact *p*-values, F-statistics, degrees of freedom, and 95% confidence intervals for all pairwise comparisons are provided in Source Data.

Hematoxylin and eosin (H&E) staining reveals typical histomorphological features across all tumors (Supplementary Fig. S16), but spatially resolved transcriptomic data enables the precise localization and annotation of tumor, immune, stromal, and vascular cell types based on previously characterized single-nucleus expression profiles (Fig. 6A–D).

Cell type deconvolution uncovered marked differences in tissue architecture between subgroups: LR tumors exhibited a compartmentalized structure, with well-demarcated tumor and stromal regions and minimal intermixing of immune or fibroblast populations (Fig. 6A–C). In contrast, the HR tumor displayed a disordered architecture, with intermingling of tumor cells with immune and stromal elements and a loss of defined compartment boundaries (Fig. 6D).

To quantitatively assess spatial organization across tumors, we computed Moran’s I spatial autocorrelation for each annotated cell type. LR tumors were largely diffuse, with most populations showing weak to negligible autocorrelation (per-sample mean Moran’s I 0.05–0.08). In contrast, the HR tumor exhibited markedly stronger spatial structuring, with the highest per-sample mean Moran’s I (0.14) and the majority of populations reaching at least moderate autocorrelation. This was most pronounced in fibroblasts, which showed strong spatial clustering in the HR tumor (Moran’s I ≈ 0.34, versus ≤0.20 in LR samples). Among tumor cell types, the Glomerulosa-like population was distinctly more co-localized in the HR tumor (≈0.14 versus ≤0.01 in LR), whereas the zF/zR-like (Cluster II) population was comparably structured across the HR and LR tumors (0.17 in HR vs. 0.08–0.21 in LR). Together, these findings indicate the emergence of localized stromal and partial tumor-cell niches within the HR tumor, consistent with a spatial consolidation of microenvironmental compartments. These spatial differences in cellular organization raised the possibility that regional microenvironmental structure may be accompanied by localized variation in signaling pathway activity. In line with this, AP-1 family members showed highest expression in the HR tumor with elevation in tumor rich regions (Supplementary Fig. S17).

To infer signaling pathway activity across spatial domains, we used the PROGENy compendium to assess pathway-responsive gene signatures. This analysis revealed elevated WNT pathway activity in the HR tumor relative to all LR subgroups, with a modest increase also detected in LR2 (Fig. 6A–D, WNT score insets). These findings align with single-cell and epigenomic evidence of WNT pathway activation in HR tumors.

Together, these findings indicate that HR pACTs are characterized not only by distinct transcriptional and epigenetic states but also by altered spatial tissue organization, including reduced global compartmentalization alongside the emergence of localized stromal and tumor niches with elevated signaling activity. This spatial reconfiguration of the tumor microenvironment may reflect microenvironmental stabilization processes associated with aggressive tumor behavior and potential immune escape.

### HDAC inhibition by entinostat reduces viability in vitro, reduces CpG island methylation and alters metabolic programs in ACC cells

Previous analyses identified the HR pACT subgroup as characterized by co-activation of WNT and AP-1 transcriptional programs, alongside an enhanced CpG Island Methylation. To explore targeted therapeutic strategies for this subgroup, we initiated drug screening using a short-term culture derived from a freshly resected metastasis from a patient with HR pACC. Among a panel of candidate compounds (Supplementary Fig. S18, Supplementary Data S8), the class I HDAC inhibitor entinostat showed the most potent anti-proliferative effect, with an IC_50_ of ~1 µM (Supplementary Fig. S18). In contrast, conventional chemotherapeutic agents such as doxorubicin required substantially higher concentrations to achieve similar levels of viability reduction.

To further dissect the molecular consequences of HDAC inhibition, we treated two adult ACC cell lines (H295R and CU-ACC1) – which share key molecular features with pediatric HR tumors - with entinostat (0.01–50 µM) for 72 h. IC_50_ values in both lines were comparable to those observed in the short-term culture, confirming consistent drug sensitivity. For comparison, we included decitabine, a DNA-demethylating agent, to assess the relative efficacy of these two epigenetic modifiers. Viability assays confirmed a dose-dependent decrease in cell viability with entinostat treatment in both cell lines, whereas decitabine had minimal effect on viability (Fig. 6E), suggesting that HDAC inhibition may be more effective than DNA demethylation alone in modulating ACC cell growth.

Genome-wide methylation profiling revealed a pronounced reduction in DNA methylation for the 1% most variable promoter-associated CpG sites upon entinostat and dectiabine treatment (Fig. 6F). This hypomethylation was observed across all treatment durations and cell lines (Supplementary Fig. S19A–E). Importantly, targeted methylation analysis for cell line H295R revealed reduced methylation at pro-apoptotic loci including *CASP8, CDKN2A*, and *BCL2L1*. CU-ACC1 showed decreased methylation of *CDKN2A, APC* and *CASP9* after entinostat treatment (Supplementary Fig. S19F-G), suggesting potential reactivation of silenced apoptotic pathways.

Dimensionality reduction using UMAP of scRNA-seq data from two cell lines revealed clear transcriptional divergence between entinostat-treated and untreated cells (Fig. 6G), indicating global shifts in gene expression following HDAC inhibition (Supplementary Fig. S20A-B). Analysis of transcription factor expression dynamics showed consistent downregulation of AP-1 components, including *FOS*, *JUND*, and *JUNB*, after treatment (Fig. 6H), aligning with our earlier identification of AP-1 as a central regulatory axis in HR-pACT and suggesting that HDAC inhibition may act through a downregulation of this high risk transcription factor program.

To confirm AP-1 pathway activity in both ACC cell lines, we performed qRT-PCR following serum starvation and re-stimulation in H295R and CU-ACC1 cells. *FOS, FOSB, JUN*, and *JUNB* were rapidly and significantly induced in both lines, with induction detectable as early as 15 min and peaking up to 132-fold for *FOSB* at 120 min in H295R cells (one-way ANOVA, all *p* < 0.001; Fig. 6I). These results confirm that AP-1 components are stimulus-responsive in both adult ACC cell lines, supporting their validity as models for the AP-1-driven program identified in HR-pACT.

Marker gene heatmaps confirmed a broad reprogramming of transcriptional states in response to entinostat, with loss of several high-risk–associated gene signatures (Supplementary Fig. S20B). Functionally, pathway-level analysis of the scRNA-seq data demonstrated significant upregulation of steroidogenic genes (e.g., *STAR, CYP11A1*), indicating a shift towards adrenal-specific metabolic programs (Supplementary Fig. S20D-E). To validate these findings in vitro, we performed qRT-PCR for key steroidogenesis genes following entinostat treatment in both cell lines. The response was gene- and cell-line-dependent: in both cell lines, *STAR* was significantly upregulated (up to 1.6-fold in H295R) while *CYP11A1* was dose-dependently downregulated (to 0.81-fold in H295R). Modest but significant upregulation of *CYP21A2* was observed at 0.5 µM in both cell lines (Tukey HSD, p < 0.05; Supplementary Fig. S20F). Together, these results indicate that entinostat does upregulate steroidogenic transcription in adult ACC lines.

Additionally, expression of pro-apoptotic genes (including *BAX, TNFRSF10B, CASP8*, and *CDKN2A*) was increased in a dose-dependent manner in both cell lines following treatment (Supplementary Fig. S21A-B), in line with the changes observed in the methylome, suggesting enhanced apoptotic susceptibility. Consistent with these changes, Annexin V / 7-AAD flow cytometry analyses revealed an increased fraction of apoptotic cells following entinostat treatment in both ACC models, supporting functional reactivation of apoptotic pathways (Supplementary Fig. S21C).

Together, these single-cell analyses from two ACC models provide evidence that entinostat induces a coordinated epigenetic and transcriptional reprogramming, reversing specific promoter associated methylation, silencing AP-1–driven oncogenic programs, upregulating steroidogenic metabolism, and upregulating pro-apoptotic pathways. Importantly, the functional activity of the AP-1 pathway in both H295R and CU-ACC1 cells was confirmed by qRT-PCR, demonstrating rapid transcriptional induction of *FOS*, *FOSB*, *JUN*, and *JUNB* upon serum re-stimulation. These findings, together with the results from the short-term culture of a pACC, underscore the therapeutic potential of HDAC inhibition to target a core molecular vulnerability of HR-pACT and may open up a path for the clinical use of these therapeutics in the clinic, probably in concert with other e.g. adrenolytic drugs already used in the treatment of pACT.

## Discussion

The biology of pACTs presents two major, clinical challenges: the absence of reliable molecular predictors for relapse or metastasis, and a lack of targeted therapeutic strategies. Although clinical scoring systems such as pS-GRAS and COG staging have been developed to estimate risk, they often fail to identify high-risk patients prior to disease progression - particularly in early-stage cases (e.g., COG stage II) that exhibit aggressive molecular features despite indolent clinical presentation^1^.

**Fig. 7:**
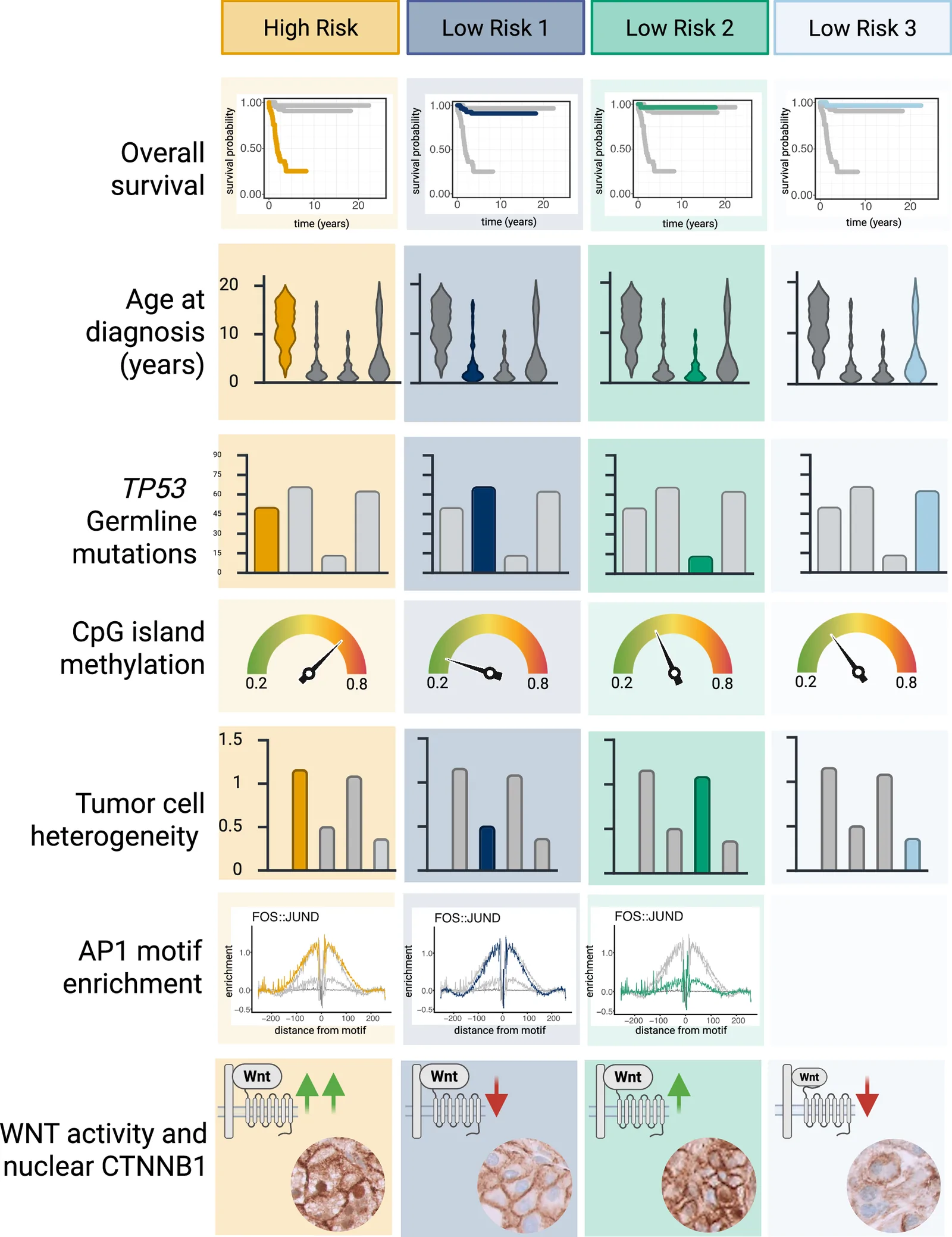
Integrative summary of molecular and clinical features across pediatric ACT subgroups. Schematic overview of Low Risk 1, Low Risk 2, Low Risk 3, and High Risk DNA methylation-based subgroups, illustrating key differences in clinical outcomes and molecular phenotypes. From top to bottom: Kaplan-Meier survival curves highlighting subgroup-specific prognosis; age at diagnosis in years; frequency of germline TP53 mutations; degree of CpG island methylation; relative tumor cell heterogeneity derived from single-cell transcriptomics; AP-1 motif enrichment based on snATAC-Seq; WNT pathway activity and CTNNB1 localization. High Risk tumors are defined by poor survival, high CpG island methylation, TP53 alterations, increased transcriptional heterogeneity, elevated WNT signaling with nuclear CTNNB1, and strong AP-1 motif enrichment—features absent or attenuated in low-risk subgroups. Created in BioRender. Fincke, V. (2026) https://BioRender.com/h5ljapn.

Our integrative epigenomic analysis identified four molecularly distinct subgroups based on DNA methylation profiles, each marked by unique transcriptional programs, cellular compositions, and tissue architectures (Fig. 7). These molecular subtypes outperformed traditional histopathological classifications in predicting clinical outcomes and addressed the longstanding lack of robust risk markers in pACT.

As consensus cluster based assignment is depending on the availability of a large reference cohort we developed a supervised machine learning classifier trained on methylation data from the full cohort. This model enables high-confidence assignment of tumor subtypes and may be particularly useful in stratifying patients with intermediate-stage disease (COG stage II–III), where relapse risk is substantial but unpredictable by clinical parameters^1,5^.

The increased CpG island methylation observed in our HR group resembles the CIMP-high phenotype previously reported in subsets of adult ACC^16^. However, while similar epigenetic patterns have been reported in other malignancies—notably IDH-mutant gliomas^19,20^—where single genomic alterations directly drive CIMP by the inhibition of TET enzymes, the genetic and metabolic determinants of this phenotype in ACTs do not appear to follow a monogenic pattern. In fact, the mechanism by which hypermethylation is established remains unknown. Recent literature considers CpG hypermethylation as a broader phenomenon, related to an increase of stem cell divisions, but not necessarily as an epigenetic driver of tumorigenesis^21^. This increased proliferative potential is mirrored in the zG-like cluster in our study – a population of cells that is not restricted to the HR group of tumors but that maintains excessive WNT signaling specifically in this subgroup. We hypothesize that HR tumors hijack this developmental pathway to maintain a proliferative, undifferentiated state.

Regulatory motif enrichment analysis revealed strong activity of TCF7L2- and AP-1—associated motifs, implicating both axes as central oncogenic drivers in HR tumors. It has been shown that joined recruitment of TCF7L2 and FOSL2, an AP-1 member, results in activated signaling pathways to promote tumor progression in EphA2-Super-enhancer tumors^22^. Unlike the LR subgroups, which activated either WNT or AP-1 components, HR tumors uniquely co-activated both pathways. This dual activation likely amplifies oncogenic signaling, consistent with previous reports showing convergence of WNT and AP-1 on shared target genes such as *MYC* and *Cyclin D1*^23^. Supporting this notion, nuclear accumulation of CTNNB1—a hallmark of WNT activation—was also observed in LR2 tumors, but without accompanying AP-1 activity, suggesting that WNT signaling alone is insufficient to shape the HR phenotype. Notably, CTNNB1 nuclear localization in the absence of somatic *CTNNB1* mutations was exclusively seen in HR tumors, which points to other factors that activated WNT signaling in these tumors.

AP-1 appears to function as a critical co-factor in oncogenic enhancer activation and has been implicated in multiple stages of tumor progression^24^. Its role in regulating cellular plasticity in other solid tumors supports its relevance in HR pACTs. For instance, in melanoma, dynamic shifts in the AP-1 transcriptional network have been shown to govern transitions between differentiation states and drive adaptive responses to MAPK inhibition^25^.

The tumor microenvironment further differentiates the molecular subgroups and likely contributes to the aggressive phenotype of HR tumors. High-resolution spatial transcriptomics revealed that HR tumors lack the organized compartmentalization observed in LR subgroups and instead exhibit an interdigitated architecture, characterized by extensive intermixing of tumor, immune, and stromal cells.

Consistent with this, snRNA-seq-based quantification of TME cell populations revealed that HR tumors are markedly depleted of macrophages and lymphoid cells compared to LR subgroups (Kruskal-Wallis, *p* = 0.047 and *p* = 0.049, respectively), suggesting an immunosuppressed microenvironment that may underpin immune evasion in this subgroup. These findings are consistent with a growing body of evidence from adult adrenocortical single-nucleus atlases. Tourigny et al. confirmed that adult ACC represents an immune-cold tumor with relative depletion of infiltrating immune cells compared to normal adrenal tissue^26^, while Altieri et al. demonstrated that macrophage abundance is reduced in cortisol-producing adenomas^27^, suggesting that glucocorticoid-mediated immunosuppression may operate across the spectrum of adrenocortical tumors from benign to malignant. Most strikingly, Jouinot et al. showed that inflammatory macrophage depletion and exhausted T cell signatures characterize aggressive adult ACC, and that these immune features combine with steroid cell programs into distinct ecotypes associated with outcome^28^. The macrophage and lymphoid depletion we observe in HR pACTs is consistent with this pattern, suggesting that a conserved immunosuppressive microenvironment characterizes aggressive adrenocortical malignancies across both pediatric and adult contexts. Regarding the tumor cell compartment, our identification of a zG-like dedifferentiated population enriched in HR tumors with active WNT signaling finds resonance in the adult literature: Altieri et al. identified a ZG-like cluster in adrenocortical adenomas enriched for CTNNB1 target genes including AFF3 and LEF1^27^, while Tourigny et al. identified a putative LGR4+ progenitor-like population in adult ACC with active WNT and NOTCH signaling^26^, suggesting that aberrant activation of ZG-like stem cell programs may be a shared oncogenic mechanism across adrenocortical tumors regardless of age. The WNT pathway activation we identify as a hallmark of HR pACTs is furthermore consistent with the known role of Wnt-βcatenin signaling in mediating T cell exclusion from the tumor microenvironment, providing a potential mechanistic link between the epigenetic and transcriptional features of HR tumors and their immune-cold phenotype. Notably, however, important differences exist between the pediatric and adult settings: pACTs arise in a developmental context with distinct epigenetic architecture, the methylation-based risk stratification we describe has no direct equivalent in adult ACC classification, and the role of AP-1 co-activation alongside WNT as a defining feature of HR pACTs represents a regulatory hallmark not previously described in adult single-nucleus studies, underscoring the biological distinctiveness of aggressive pediatric adrenocortical tumors.

The heterogeneous cellular composition of HR tumors also aligns with the role of AP-1 in enforcing transcriptional plasticity, which has been described in cancer entites including melanoma^25^. In colorectal cancer it has recently been ascribed a role in oncofetal reprogramming - a process that can be targeted by experimental inhibitors^29^. It was also described as a gatekeeper in reprogramming somatic cells to pluripotent stem cells by Markov et al.^30^. Thus, AP-1 may enable tumor cells to respond flexibly to microenvironmental cues, fostering aggressive behavior and phenotypic adaptation.

Beyond characterizing functional composition and signaling in the subgroups of pACT, we sought to use pharmacological perturbation as a tool to further interrogate the epigenetic dependencies of HR tumors. Generally, pACCs are notoriously hard to treat by chemotherapy, with the adrenolytic drug mitotane being the only systemic therapy that has shown modest efficacy^31^. We hypothesized that the cooperative epigenetic framework of AP-1 motif accessibility and increased CpG island methylation, in which oncogenes and pro-proliferative genes are activated while tumor suppressors and pro-apoptotic genes are silenced, might be best addressed with HDAC inhibition. This hypothesis is fueled by earlier reports, suggesting that HDAC inhibitors suppress proliferation governed by FOS::JUN signaling^32^.

Our findings suggest that HDAC inhibition with entinostat as a mechanistic tool to explore the epigenetic dependencies of the HR group. We note that prior large-scale drug screening in the same ACC cell lines identified IC50 values for entinostat in the range of 3–5 μM, indicating limited cytotoxic potency at pharmacologically relevant concentrations^33^. Consistent with this, our data should be interpreted primarily in a biological rather than a therapeutic context.

As shown in our short-term culture from an HR pACC, the function of HDACi might be relevant in HR pACTs, where CpG island hypermethylation coexists with strong transcriptional activation of AP-1-regulated networks. This might create a therapeutic vulnerability that cannot be addressed by DNA-demethylation alone. The observed demethylation in cell line models at key pro-apoptotic loci upon entinostat treatment implies that entinostat can potentially overcome one of the central resistance features of aggressive tumors. In parallel, snRNA-seq analyses revealed a reprogramming toward endocrine differentiation—hallmarks typically lost in HR tumors—indicating a partial restoration of normal adrenal cortical function. While HDAC inhibitors have shown limited success as monotherapies in other solid tumors, their activity in ACC remains unexplored, positioning entinostat as a rational candidate for future in vivo testing - potentially in combination with other epigenetic or targeted therapies.

Despite the depth and integrative nature of our study, several limitations warrant consideration. First, while our spatial transcriptomics and snATAC-seq data provide critical insight into tumor architecture and chromatin accessibility, these analyses were performed on a limited number of representative tumors. Second, our functional studies relied mainly on adult ACC cell lines due to the current lack of pediatric-derived tumor models—a limitation shared broadly across rare pediatric solid tumors, where the scarcity of patient-derived cell lines and models has been repeatedly identified as a major bottleneck to target discovery and therapeutic development^34–37^. While we demonstrate that these adult lines recapitulate key epigenetic features of HR pACT the developmental context of pediatric tumors may not be fully captured in these systems. To partially address this, we also included a short-term culture derived from freshly resected HR pACC tissue, which showed comparable drug sensitivity to the established cell lines. However, its limited lifespan precludes longer-term mechanistic studies. Moving forward, the development of more faithful pediatric models - such as patient-derived organoids, xenografts, or engineered systems - will be essential to functionally validate subgroup-specific vulnerabilities and to guide therapeutic translation.

Although our DNA methylation-based classifier achieved high predictive performance in cross-validation and was developed using a geographically and ethnically diverse dataset - including samples from the German MET cohort, as well as from North and South American cohorts^17,18^ - its application was still limited to retrospective data. This mirrors the trajectory of methylation-based classification in other tumor entities, where similar tools have progressed from retrospective proof-of-concept to routine diagnostic use, as demonstrated for central nervous system tumors^38^, sarcomas^39^, and, in the pediatric rare-tumor context most directly comparable to pACT, extracranial malignant rhabdoid tumors (eMRT)^36^. Future prospective validation on diagnostic biopsies, particularly in treatment-naive patients, will be essential to confirm its clinical utility and ensure robust performance across healthcare settings.

Based on the molecular subgroups and clinical associations described in this study, we propose a preliminary decision tree for risk stratification of pACT patients at diagnosis (Supplementary Fig. S22). In this framework, histological subtype serves as the first decision node, with pACA classified as benign requiring no further molecular workup. For pACC and pUMP, DNA methylation-based classification using the EPIC array is proposed as the primary stratification tool, directly assigning patients to LR1–3 or HR subgroups with associated treatment implications. In settings where methylation profiling is not immediately available, interim risk assessment using CTNNB1 IHC and mutation status in combination with age serves as a surrogate, with methylation profiling recommended as soon as feasible to confirm the classification. This framework requires prospective validation in independent cohorts before clinical implementation.

In summary, our study identified clinically and biologically relevant subgroups of pACTs and uncovered a cooperative WNT-AP-1 transcriptional network and increased CpG island methylation as defining hallmarks of HR disease. Cooperativity between WNT1 and AP-1 has to our knowledge not been linked to TP53 deficient tumors so far, thus the herewith presented data constitute a reference for further studies in entities with the same genetic background such as e.g. TP53 deficient sarcomas or carcinomas.

Our dataset and analyses provide a comprehensive resource for future studies of adrenocortical tumors, support risk-adapted clinical decision-making, and identify potential opportunities for epigenetic therapeutic strategies in aggressive pACC.

## Methods

### Patient material

Patient Material, clinical data and written consent from all included patients (*n* = 109) were obtained from the German MET-Registry. The MET studies are conducted in accordance with the Declaration of Helsinki and were approved by the ethics committees of the University of Luebeck (IRB 97125) and Otto-von-Guericke-University Magdeburg (IRBs 174/12 and 52/22), Germany. Written informed consent including data and bio-specimen usage in scientific projects was obtained from all patients and/or legal guardians at the time of registry enrollment, in accordance with national regulations. The MoPACT project (IRB 23-0351) and the EpiGenPAT project (IRB 21-0997) were approved by the Ethics Committee of the Ludwig Maximilians University Munich, Germany. Tumor samples were collected as fresh frozen (FF) tissue and formalin-fixed paraffin-embedded (FFPE) Material. Germline control samples were obtained from blood or control tissue. All materials, except for FFPE samples, were stored at −80 °C.

DNA and RNA were extracted from blood, fresh frozen control tissue and tumor samples using the DNA/RNA AllPrep Mini Kit (Qiagen), Maxwell RSC Blood DNA Kit (Promega) according to the manufacturer’s instructions and protocol. The RNA quality was measured at the 2100 Bioanalyzer (Agilent) by using Agilent RNA 6000 nano to calculate RNA integrity Number (RIN). Only RNA from samples with a RIN > 6 was included in the study. DNA and RNA from FFPE Material was extracted by using the AllPrep DNA/RNA FFPE Kit (Qiagen) and the Maxwell RSC FFPE Plus DNA Kit (Promega).

### RNA-microarrays

Bulk Tumor-RNA was analyzed on the GeneChip™ Human Genome U133 Plus 2.0 Array (ThermoFisher) at the Microarray Core Facility of the DKFZ (Heidelberg, Germany). All preparing steps were performed according to the recommended protocol from the manufacturer. Raw data was normalized by the MAS5.0 algorithm and log2-transformed (log2(x + 1)).

On the normalized data we performed unsupervised clustering of the expression profiles by UMAP using R and all dependent packages. Differential expression profiles over the 4 different subgroups were performed using the Bioconductor package limma (Ritchie et al. 2015).

For unsupervised clustering based on the bulk transcriptomic data, we used the 150 most variable expressed genes by standard deviation and performed umap analysis with the settings: n_neighbors = 5, n_components = 2, min_dist =0.1, metric =“pearson”.

Differential gene expression analysis was done by using the limma package (v.3.64.0^40^,). We compared one methylation-based subgroup against all other subgroups to see significant differences. To adjust multiple testing, we used Benjamini-Hochberg correction.

### DNA-methylation

DNA from fresh frozen and FFPE Material from 109 Patients were analysed using the Illumina Infinium Human Methylation EPIC and EPICv2 Bead Chip according to the Illumina protocol and the manufacturer’s instructions. DNA methylation data were generated at the Core Facility of the DKFZ (Heidelberg, Germany) using 500 ng of extracted DNA from fresh frozen tissue and 250–500 ng extracted DNA from FFPE Material. Additional Raw IDAT-files were downloaded from Clay et al. 2019 (*n* = 60^17^,) and Bueno et al. 2022 (*n* = 57^18^,) at the NCBI Gene Expression Omnibus under the accession numbers GSE131350 and GSE179175. Raw IDAT files were processed, normalized, filtered and batch effect corrected as described in Capper et al. 2018 by using the Bioconductor package minfi^38,41^.The calculated batch effect factors were extracted and used for random forest calculation and calculation of cell line betas.

### Clustering

Methylation based subgrouping was performed by using the Bioconductor package ConsensusClusterPlus^42^ with pearson distance and partitioning around medoids (PAM) based on the 5000 most variable methylated CpG-sites. Detailed settings we used are maxK=6, reps=1000, pItem=0.8, pFeature=1, clusterAlg=pam and distance=pearson. Methylation data was uploaded to NCBI Gene Expression Omnibus (GEO) under accession number GSE308221.

Visualization of unsupervised Clustering was performed using the CRAN package pheatmap (v1.0.13^43^,) and ComplexHeatmap^44^. Following settings were used: For dimensional reduction of the data, we plotted a umap with the 5000 most variable cpg sites for each sample with the CRAN package umap^45^. For reproducibility the seed was set and the detailed settings used are: n_neighbors= 15, n_components = 2, min_dist =0.1, metric=euclidean.

### Random forest classifier development

Following Capper et al. (2018), a random forest classifier was trained on DNA methylation beta values^38^. Variable selection was performed by fitting random forests on subsets of probes (~10,000 per set) with mtry set to the square root of the total number of probes. Class imbalance was addressed by down-sampling to the smallest class size. Probe importance was assessed by the permutation-based mean decrease in classification accuracy, and the top-ranked probes were retained. The final classifier was trained with 10,000 trees using the selected probes, with down-sampling applied as above. Settings: y= list of subgroups, seed = 180314, ntrees = 10000, probes = 10000;randomForest(betasy,y,ntree=10000,mtry=sqrt(ncol(betas)),sampsize=rep(min(table(y)),length(table(y))),proximity=TRUE,oob.prox=TRUE,importance=TRUE,keep.inbag=TRUE,do.trace=FALSE, seed=seed).

### Score calibration

Random forest vote proportions were recalibrated to class probabilities using penalized multinomial logistic regression (glmnet, L2 penalty). Independent random forest predictions obtained by cross-validation were used as inputs to avoid overfitting.

### Cross-validation

Performance was assessed by threefold nested cross-validation. In each fold, two-thirds of the data were used for training and one-third for testing. Nested resampling ensured that calibration models were fitted on independent random forest scores. After the threefold cross-validation each third of the dataset was used for testing.

### Performance evaluation

Classifier performance was evaluated using misclassification error, multiclass area under the ROC curve (AUC), and the multiclass Brier score.

### Implementation and availability

For practical application and accessibility, the trained classifier was implemented in an interactive R Shiny application. The tool is deployed via shinyapps.io (https://pact-classifier.shinyapps.io/pact-classifier/), allowing users to upload DNA methylation data and obtain classifier predictions through a web-based interface without requiring local software installation.

### Copy Number profiles

DNA copy-number profiles were generated using the Bioconductor package conumee2 under default parameters^46^. The intensities of measured CpG sites were normalized against healthy human control tissue using the conumee2 approach. The resulting profiles were visualized with conumee2’s built‑in plotting functions, and binned outputs were created for further analysis in the IGV browser (2.16.2) as well as for generating segmentation files for GISTIC2.0 analysis^47^. GISTIC2.0 was used with default settings with GenePattern Cloud service (https://cloud.genepattern.org/gp).

### CpG-island methylation

The DNA methylation signature was defined according to Zheng et al.^16^, based on the top 1% most variably methylated CpG sites located in island regions. To compare our four DNA methylation–based subgroups with the TCGA cohort, raw IDAT files and annotations were obtained from Zheng et al. (2016)^16^. Twelve healthy adrenal tissue samples from Clay et al. (2019) served as the control group^17^. Statistical comparisons between subgroups were conducted using the Kruskal–Wallis test (p < 0.05), followed by a post-hoc Dunn test.

### Survival analysis

Overall survival, 5‑year overall survival, and median survival were calculated as the time from diagnosis to death from disease. The most recent follow-up data were verified, and patients who were still alive at that time were censored. Several clinical and molecular parameters were analyzed to evaluate their impact on survival outcomes, including DNA methylation patterns, Ki-67 proliferation index, COG stage, and pS-GRAS score. The Ki-67 index was categorized according to Riedmeier et al. (2024): Group 1 (0–9%), Group 2 (10–19%), and Group 3 ( ≥ 20%)^48^.

Univariate Kaplan–Meier analysis was used as the statistical method. Kaplan–Meier curves were visualized with survminer (v0.5.0), with risk tables and log-rank *p* values displayed. Time-dependent AUC curves for all prognostic models were plotted to allow direct visual comparison. ps-GRAS score was calculated according to Riedmeier et al.^48^.

### Statistical analysis

To compare germline TP53 and somatic CTNNB1 mutation frequencies between the DNA methylation-based subgroups, we performed an overall two-sided Fisher’s exact test across the full contingency table, followed by pairwise two-sided Fisher’s exact tests between all subgroup pairs (Rstatix package v0.7.2), with p values adjusted for multiple comparisons using the Benjamini-Hochberg method. Bar chart visualisation was done using graphics (v.4.5.0).

The post-hoc test following the Kruskal-Wallis test was selected according to group size: two-sided Dunn’s post-hoc tests for the DNA-methylation, age and cell line analyses. Exact pairwise Wilcoxon rank-sum tests for the expression analyses, where group sizes were smaller. All *p* values were adjusted for multiple comparisons using the Benjamini-Hochberg method.

To assess the prognostic performance of different risk stratification systems, we computed time-dependent receiver operating characteristic (ROC) curves and area under the curve (AUC) values at 1–12 years post-diagnosis using the timeROC R package^49^. The following prognostic models were evaluated: pS-GRAS score (*n* = 49), DNA methylation–based molecular subgroups (*n* = 214), COG/IPACTR stage (*n* = 165), Ki-67 proliferation index [0: 0–9%, 1: 10–19%, 2: 20 or above] (*n* = 49), and Wieneke histopathological score (*n* = 209). Subgroup analyses were performed for patients with intermediate-risk disease (COG/IPACTR stages II–III, *n* = 30).

### Whole exome sequencing

Genomic DNA was extracted from tumor tissue or blood using standard procedures, as explained above. Whole exome sequencing was performed by Eurofins Genomics (Germany) using the INVIEW Human Exome Standard 50x service. Exome enrichment was conducted with the Agilent SureSelect Human All Exon V8 kit, targeting all protein-coding regions of the human genome. Sequencing was carried out on an Illumina platform, generating paired-end reads of 2 ×  150 bp. Bioinformatics processing included quality control, alignment to the GRCh37/hg19 reference genome, and variant calling. The mean coverage across target regions was 50×. All laboratory procedures were conducted according to ISO 17025-certified protocols. Raw sequencing data were adapter-trimmed using TrimGalore, mapped to the reference genome with BWA-MEM, and duplicates were marked using Picard. Variants were called using six different algorithms (Freebayes, HaplotypeCaller, bcftools mpileup, Strelka, Platypus, and VarScan2) and filtered for quality and coverage parameters. Variants were annotated with VEP and filtered against gnomAD and in-house control datasets.

### Immunohistochemistry

Immunohistochemistry for β-catenin was performed on formalin-fixed, paraffin-embedded sections using a mouse monoclonal antibody (Cell Marque, Ref: 224M-15) at a 1:50 dilution. Staining was conducted on a Ventana Benchmark Ultra platform with OptiView DAB detection. Antigen retrieval was performed with CC1 buffer (24 min, 100 °C), followed by antibody incubation for 16 min. Membranous staining of β-catenin is considered physiologic, while nuclear staining often indicates activation of the Wnt/β-catenin pathway^50,51^, commonly due to mutations in the CTNNB1 gene.

### Single nucleus preparation

Single-nucleus suspensions were generated using the protocol described by Slyper et al. (2020^52^,) for fresh frozen tumor tissue (*n* = 29). Samples were prepared on ice using a cold CHAPS/ST buffer. The tissue was cut and chopped into small, homogeneous pieces and then digested in the buffer. After digestion, the suspension was filtered through two 40 µm cell strainers. To ensure complete recovery, the filters were washed three times with an ST buffer (salt-Tris). The samples were then centrifuged for 5 min at 500 × *g* at 4 °C and subsequently resuspended in PBS containing 0.05% BSA. Cell counts were performed using a Neubauer counting chamber, targeting a recovery of 6000 cells per sample. Nuclei were loaded into the Chromium system (10X Genomics). Library preparations for Chromium Next GEM Single Cell 5’, Chromium Next GEM Single Cell 3’, and Chromium Single Cell Multiome were performed according to the manufacturers’ instructions and protocols. The quality and concentration of cDNA and libraries were assessed using the Qubit dsDNA HS Assay Kit (Thermo Fisher Scientific) and the Agilent 2100 Bioanalyzer. Equimolar pooled libraries (multiplexes) were sequenced on a NovaSeq 6000 (Illumina) sequencer, according to the manufacturer’s instructions.

### Preparation for downstream analysis

Raw sequencing data from 10x Genomics Chromium single cell RNA-seq (using both 5’ and 3’ chemistry kits) and single cell Multiome (ATAC + gene expression) experiments were processed using the Cell Ranger software suite (v8.0.1, 10x Genomics). Demultiplexing of raw base call files (BCL) into FASTQ format was accomplished with the cellranger mkfastq command. Single cell RNA-seq datasets were further processed using cellranger count, mapping reads to the GRCh38 human reference genome and generating feature-barcode matrices for both 5’ and 3’ libraries. Single cell Multiome data were processed using Cell Ranger ARC, with separate pipelines for ATAC (cellranger-atac count) and multiome (cellranger-arc count), producing gene expression and chromatin accessibility matrices linked for each cell. Quality control and filtering were performed according to the default criteria specified by Cell Ranger. The resulting filtered count matrices and feature-barcode matrices were imported into R(v4.2.0), further processed using Seurat (v5.3.0^53^,) and thus served as input for all downstream analyses.

### Analysis of single nucleus RNA sequencing

#### Quality control (QC)

For each RNA sample, mitochondrial gene content was calculated using the PercentageFeatureSet() function with the pattern “^MT-” to identify mitochondrial transcripts. Samples were subset based on thresholds for the number of detected features and mitochondrial transcript percentages. Cells with fewer than 200 genes were excluded. Additionally, cells with high mitochondrial transcript content were filtered to remove dead or stressed cells. To minimize doublet contamination, we employed the scDblFinder package. Each dataset was processed using the scDblFinder() function from the scDblFinder package (v.1.22.0^54^,). Doublets were identified and removed, retaining only singlets for further analysis.

### Normalization, feature selection, and dimensionality reduction with seurat

Normalization of each dataset was performed using the NormalizeData() function to standardize expression values. Highly variable genes were identified using the FindVariableFeatures() function with the variance-stabilizing transformation (VST) method, selecting the top 2000 variable features. The data were then scaled using the ScaleData() function, where all genes in the dataset were included in the scaling process. Principal component analysis (PCA) was applied using the RunPCA() function, focusing on the previously identified variable features. Following PCA, the data were projected into a lower-dimensional space using the Uniform Manifold Approximation and Projection (UMAP) algorithm with the RunUMAP() function, utilizing the first 20 principal components. A nearest neighbor graph was then constructed using the FindNeighbors() function with the first 20 principal components. Clustering of cells was performed using the Louvain algorithm through the FindClusters() function, setting the resolution parameter to 0.5. Visualization of the clustered cells was achieved using the DimPlot() function, which generated a UMAP plot with clusters labeled based on the seurat_clusters metadata field. All plots shown in figures have been generated using Seurat (v5.3.0^53^,), SCpubr (v3.0.0^55^,) or ggplot2 (v3.5.2^56^,).

### Integration and dimensionality reduction

To integrate multiple datasets into a single object for analysis, the individual datasets were merged using the merge() function. This dataset included samples from multiple conditions, with each sample assigned a unique cell identifier using the add.cell.ids parameter. The combined dataset was then processed for normalization, feature selection, and scaling. Highly variable genes were identified using the FindVariableFeatures() function with the variance-stabilizing transformation (VST) method, selecting the top 2000 variable features. The data were scaled using the ScaleData() function, and principal component analysis (PCA) was applied using the RunPCA() function, retaining the top 20 principal components for further analysis.

For batch effect correction and dataset harmonization, the Harmony algorithm was applied using the RunHarmony() function (v1.2.3^57^,), with sample identity as the batch variable. The batch-corrected dataset was then projected into a lower-dimensional space using UMAP (RunUMAP()), based on the first 20 Harmony components. Following this, a nearest neighbor graph was constructed using the FindNeighbors() function, and clustering of cells was performed using the Louvain algorithm through the FindClusters() function, setting the resolution parameter to 0.5.

### Cell cycle scoring and phase assignment

Cell cycle phase scoring was performed using the CellCycleScoring() function from the Seurat package. Canonical S phase and G2/M phase gene lists were obtained from cc.genes as provided in Seurat’s built-in reference (s.genes, g2m.genes). These signatures were used to assign cell cycle scores and phases (G1, S, or G2/M) to individual cells in the object. Post-scoring, the S and G2M phase scores as well as the assigned cell cycle phase were extracted and summarized by cell type using dplyr (v1.1.2^58^,). For each annotated cell type, mean S and G2M scores were calculated, along with the proportion of cells in each phase (G1, S, G2/M) and total cell counts.

### Trajectory inference and pseudotime analysis

Trajectory analysis was performed using the Monocle 3 package (v1.4.26^59^,). The single-cell Seurat object was converted to a cell_data_set object using as.cell_data_set(). Cells were clustered using the Leiden algorithm (cluster_cells, resolution = 1e–3), and only cells belonging to the main partition (monocle3_partitions == 1) were retained for downstream analysis. A principal graph was learned on the filtered dataset (learn_graph) with default parameters, and root cells were manually defined based on a cluster presumed to represent the earliest state in the differentiation trajectory. Cells were ordered along the inferred trajectory using order_cells(), and pseudotime values were computed.

Pseudotime was visualized using plot_cells() and mapped back to the integrated Seurat object via AddMetaData(). Differential gene expression along the trajectory was assessed using graph_test() based on the principal graph. Final pseudotime values were visualized using FeaturePlot(), stratified by molecular risk group.

### Non-negative matrix factorization (NMF) analysis

To uncover dominant transcriptional programs in tumor cells, we applied non-negative matrix factorization (NMF) as previously described in Blanco-Carmona et al.^60^. NMF was performed independently for each patient’s tumor cell compartment, excluding non-malignant (TME) nuclei. Normalized and scaled expression matrices were factorized using ranks *ĸ*=2 to 10, and the top 30 contributing genes per program (excluding mitochondrial genes) were retained.

To score individual nuclei for program enrichment, each gene was compared against a control set of 100 expression-matched genes, and enrichment scores were averaged across each nucleus. Pearson correlation was then used to cluster similar NMF programs into NMF metaprograms, which were annotated using the enrichR package. Final gene scores were computed per metaprogram, and enrichment across the integrated dataset was visualized using Seurat::AddModuleScore().

### Cell–cell communication inference using LIANA

To investigate intercellular communication across DNA methylation-based subgroups, we applied the LIANA (LIgand–Receptor ANalysis frAmework, v0.1.14^61^,) pipeline to processed 5′ single-nucleus RNA-seq data for each risk group (HR, LR1, LR2, LR3). For each dataset, cell type annotations were used as identity classes, with a specific focus on interactions involving the glomerulosa-like tumor cell population as the source.

Per sample, we used the liana_wrap() function to compute ligand–receptor interactions based on gene expression, followed by liana_aggregate() to summarize results across methods. Dot plots of the top 20 interactions were generated using liana_dotplot(), comparing source signals from glomerulosa-like clusters to target populations including zF/zR-like Cluster I, zF/zR-like Cluster II, and self-interactions. To minimize matrix-associated confounding, collagen-based ligands (ligand.complex starting with “COL”) were excluded from visualization using string filtering (!str_starts(ligand.complex, “COL”)).

### Single nucleus multiome sequencing

We processed the snMultiome data following the Signac (R v.1.14.9001^62^,) vignette for joint RNA and ATAC analysis by creating a multimodal Seurat object. Filtering parameters were applied as specified in the vignette: TSS enrichment > 1, nucleosome signal <4, nCount ATAC > 1800, and nCount RNA > 1000, with upper thresholds adjusted based on Signac QC plots to retain only high‑quality cells. Data were normalized using the SCT function, and cell type annotations were transferred from the snRNA analysis. Unlabeled cells were annotated according to the dominant cell type in the specific Seurat based cluster of the transcriptomic data. After performing principal component analysis (PCA), we generated UMAPs for RNA, ATAC, and a joint RNA/ATAC integration. Batch effects were corrected using Harmony (v. 1.2.3^57^,). In downstream analyses, non‑neoplastic cells were excluded. Differential and overrepresented motif analyses across the DNA methylation‑based subgroups were performed with Signac, using the BSgenome.Hsapiens.UCSC.hg38 annotation from Bioconductor as reference genome. All steps were done according to the vignette. ChromVAR (R v1.28.0^63^,) was used to calculate differential activity between DNA-Methylation based subgroups. Co‑accessible sites were calculated with the cicero package^64^, and additional motif footprinting analysis was conducted. Finally, trajectory analysis was performed using the scMEGA package (v.1.1.0^65^,) to assess transcription factor activity along pseudotime. Transcription factor, gene selection and Gene regulatory network inference (GRN) was performed according to the vignette. After this GRN was plotted along the trajectory.

### Spatial transcriptomics

Four FFPE adrenocortical tumor slides were processed according to the manufacturer’s standard preparation protocol for VisiumHD (10x Genomics). The raw data were converted into count matrices using Spaceranger (v 3.1.3), as reference for the transcriptome data the “Visium Human Transcriptome Probe Set v 2.0 GRCH38 2020 A” was used. CytAssist (10x Genomics) pictures were manually aligned with the integrated “Visium Image Alignment” function of 10x Genomics Loupe Browser (v8.1.2).

### Quality control

The 2 µm spaceranger output was loaded in python (v3.10) with the bin2cell loading function and filtered for genes which are present in at least 3 bins and bins with at least one count. Image alignment and nucleus detection was performed according to Polański et al. 2024 (https://github.com/Teichlab/bin2cell^66^,) with a mpp=0.3. Celltype annotation was transferred from the annotated sn-RNA data by training the celltypist classifier^67^ with transcriptomic data of 5000 cells and a top gene number of 300. Predicted celltype labels were transferred to the AnnData object^68^. Only cells with 4 or more bins were obtained and then cells with less than 100 genes were filtered out. Scanpy’s^69^ QC metrics and highly variable genes were calculated for the top 5000 genes, and the data was normalized and log transformed. Cell type distribuition was calculated for each sample in percent according to the total cell number.

Downstream analysis was performed using Liana+^61^. The Liana+ implemented function was used to get progeny annotation^70,71^. For Pathway score calculation the top 50 genes were used. A representative tumor section was selected for each tumor and plotted with a zoomed box.

To assess the spatial distribution of the annotated cell populations across each H&E section, global Moran’s I was computed for every cell type in all subgroups. Cell-type labels were taken from the CellTypist annotation described above, and for each cell type a binary occupancy vector was defined over the bin2cell-segmented cells (1 = assigned to that cell type, 0 = otherwise). Because bin2cell reconstructs cells as irregularly spaced centroids, spatial neighborhood graphs were built on the cell centroids with Squidpy’s gr.spatial_neighbors^72^ in generic mode (coord_type = ‘generic’) using Delaunay triangulation, and edges exceeding the 99th percentile of edge lengths were removed to avoid spurious connections across tissue gaps. Spatial weights matrices were derived from the resulting connectivity matrices using WSP and WSP2W from libpysal and row-standardised (Rey and Anselin, 2007). Cell populations represented by fewer than 20 cells in a given section were excluded. Moran’s I was computed with 999 conditional permutations as implemented in the esda library (Rey and Anselin, 2007). Given the large number of cells characteristic of VisiumHD data, permutation-based *p*-values are constrained to a minimum of 1/(n_permutations+1) irrespective of effect size; results were therefore interpreted on the basis of effect size alone. Moran’s I effect size was categorised as strong (I ≥ 0.3), moderate (0.1 ≤ I < 0.3), weak (0.05 ≤ I < 0.1), or negligible (I < 0.05).

Per-panel summary statistics for each gene (DNMT3A, FOS, JUN, JUND) and all risk groups was calculated. Descriptive summary statistics were computed on the same log-normalised expression matrix that the panels display. A cell was considered positive for a gene if its expression value was greater than zero. For each gene × section panel we report the number and percentage of positive cells, together with the mean and median log-normalised expression across all cells and across positive cells only; the percentage of positive cells and the underlying cell counts are annotated on each panel. The fraction of positive cells per gene is additionally summarised as a boxplot of the three low-risk sections, with HR pACT overlaid as an individual reference point.

### Drug screening on primary pACC culture

Drug screening was performed on a freshly resected lung metastasis from a pediatric adrenocortical carcinoma patient. Tissue processing and culture conditions were carried out as previously described by Peterziel et al.^73^. Briefly, tumor material was mechanically dissociated and enzymatically digested, followed by filtration and red blood cell lysis. Cells were cultured in serum-free TSM complete medium, monitored for spheroid formation, and seeded for drug screening within 2–7 days post-isolation, depending on morphology and growth behavior.

Drug screening was conducted in 384-well round-bottom plates pre-loaded with 75 clinically relevant anticancer agents at five concentrations across five logs, including targeted therapies and standard chemotherapeutics. Each condition was tested in duplicate, alongside positive (benzethonium chloride), intermediate (staurosporine), and negative (DMSO) controls. Cell viability was assessed 72 h post-treatment using CellTiter-Glo® 2.0. Normalized % inhibition was calculated relative to control wells.

### Cell culture

The human adrenocortical carcinoma cell line H295R was obtained from Cytion (catalog no. 300483). The adrenocortical carcinoma cell lines CU-ACC1 were kindly provided by the University of Colorado Medical Campus (Aurora, CO, USA). Cell line identity was confirmed based on documentation provided by the original source (CU-ACC1) or the commercial supplier (H295R from Cytion) and on consistency of molecular and phenotypic characteristics with previously published descriptions, including characteristic copy number alterations as seen by DNA methylation profiling. Both H295R and CU-ACC1 were routinely tested and found to be negative for mycoplasma contamination.

For therapeutic approaches the cell lines ACC1 and NCI-H295R were cultivated. NCI-H295R were cultivated using DMEM F12, 5% FBS, 0.00625 mg/ml Insulin, 0.00625 mg/ml Transferrin, 6.25 ng/ml Selen (ITS –X solution), 1.25 mg/ml bovine serum albumine, 0.00535 mg/ml linoleic acid.

For ACC1 we used F12 medium: 3:1 (v/v) F12 and DMEM high glucose, 5% FBS, 0.4 µg/ml hydrocortisone, 5 µg/ml Insulin, 8.4 ng/ml cholera toxin, 10 ng/ml epidermal growth factor, 24 µg/ml adenine. Cell lines were routinely tested for mycoplasma contamination and confirmed negative.

### Serum starvation and AP-1 induction assay

H295R and CU-ACC1 cells were seeded and grown to approximately 70–80% confluency under standard culture conditions. To induce quiescence, cells were washed once with phosphate-buffered saline (PBS) and incubated in serum-free medium for 24 h. AP-1 pathway activation was subsequently triggered by re-addition of complete medium supplemented with 5% FBS. Cells were harvested at 0, 15, 30, 60, and 120 minutes after serum re-stimulation by direct lysis in the appropriate buffer.

### Quantitative RT-PCR

Total RNA was isolated using the Maxwell RSC simplyRNA Cells Kit on the Maxwell RSC instrument (Promega) according to the manufacturer’s instructions. RNA quantity was assessed by Qubit (Thermo Fisher Scientific). Complementary DNA (cDNA) was synthesised from 500 ng total RNA using Biozym cDNA Synthesis Kit according to the manufacturer’s protocol. Quantitative RT-PCR was performed for genes listed in Supplementary Data 9, with HPRT1 and TBP as reference genes, using SYBR Green chemistry on a QuantStudio 6 Pro (Thermo Fisher Scientific). Relative expression was calculated using the ΔΔCt method, normalised to the geometric mean of both reference genes, and expressed as fold change relative to the 0-minute time point.

### Apoptosis detection by flow cytometry

Apoptosis was quantified using the Annexin V Apoptosis Detection Kit (STEMCELL Technologies, #100-0338) according to the manufacturer’s instructions. Briefly, ACC1 and H295R cells were treated with entinostat at 0.5 μM or 1 μM for 72 h or left untreated as controls. Following treatment, cells were harvested, washed, and stained with Annexin V and 7-AAD. Data were acquired by flow cytometry and cells were classified into four populations based on staining pattern: viable cells (Annexin V⁻/7-AAD⁻), early apoptotic cells (Annexin V⁺/7-AAD⁻), late apoptotic cells (Annexin V⁺/7-AAD⁺), and necrotic cells (Annexin V⁻/7-AAD⁺). The total apoptotic fraction was defined as the sum of early and late apoptotic populations.

### Use of large language modells (LLM’s)

During the preparation of this work, the authors used ChatGPT (OpenAI) and Claude (Anthropic) to improve the wording, grammar, stylistic clarity of the text and as a supportive tool for debugging analysis code. All AI-assisted text and code were reviewed, tested and verified by the authors.

### Reporting summary

Further information on research design is available in the Nature Portfolio Reporting Summary linked to this article.

## Supplementary information

Supplementary Information

Description of Additional Supplementary Information

Supplementary Data S1

Supplementary Data S2

Supplementary Data S3

Supplementary Data S4

Supplementary Data S5

Supplementary Data S6

Supplementary Data S7

Supplementary Data S8

Supplementary Data S9

Reporting Summary

Transparent Peer Review file

## Source data

Source Data

## Data Availability

Data generated in this study have been deposited in the Gene Expression Omnibus (GEO) under accession codes GSE308221 (DNA methylation, patient samples https://www.ncbi.nlm.nih.gov/geo/query/acc.cgi?acc=GSE308221), GSE343489 (Affymatrix Array, patient samples https://www.ncbi.nlm.nih.gov/geo/query/acc.cgi?acc=GSE343489), GSE316779 (Visium HD https://www.ncbi.nlm.nih.gov/geo/query/acc.cgi?acc=GSE316779), GSE336709 (DNA methylation, cell lines https://www.ncbi.nlm.nih.gov/geo/query/acc.cgi?acc=GSE336709), and GSE336546 (scRNA-seq, cell lines https://www.ncbi.nlm.nih.gov/geo/query/acc.cgi?acc=GSE336546). Whole-exome sequencing data generated in this study are available under restricted access via the German Human Genome-Phenome Archive (GHGA) under accession https://data.ghga.de/study/GHGAS86868931177408. Single-nucleus sequencing data generated in this study are available under restricted access via GHGA under accession https://data.ghga.de/study/GHGAS91001004381672. Access to restricted-access data is limited to researchers with a bona fide scientific research purpose, in accordance with data protection regulations governing patient-derived genomic and clinical data under German and EU law (GDPR), which prohibit unrestricted public release of individual-level sequencing data. Access requires submission of a data access request to the study’s Data Access Committee (DAC) via the GHGA data portal, including a description of the intended research use and confirmation of compliance with the approved Data Access Policy for the study. Requests are typically reviewed and answered within 2–4 weeks. Once granted, access remains valid for 12 months, renewable upon request. No dataset from this study has been separately deposited in the European Genome-phenome Archive (EGA); all restricted-access sequencing data generated in this study are held exclusively in GHGA. The publicly available DNA-Methylation array data from Clay et al. 2019 used in this study are available in the GEO database under accession code GSE131350 is available with the following link https://www.ncbi.nlm.nih.gov/geo/query/acc.cgi?acc=GSE131350^17^. The publicly available DNA-Methylation array data from Bueno et al. 2022 used in this study are available in the GEO database under accession code GSE179175 is available with the following link https://www.ncbi.nlm.nih.gov/geo/query/acc.cgi?acc=GSE179175^18^. Source data are provided with this paper.

## Code availability

Code is available via Zenodo (https://doi.org/10.5281/zenodo.21887084^74^). Standard software packages and their versions used for all other analyses are described in the Methods.
