# Supplementary material for: CpG hypermethylation and WNT/AP-1 cooperativity define the epigenetic landscape and a clinical subgroup of high-risk pediatric adrenocortical carcinoma
Source: Nat Commun. 2026 Sep 14;17:9786. doi: 10.1038/s41467-026-77225-5 (PMC13575229; doi:10.1038/s41467-026-77225-5)
Supplement: Supplementary file 1 [file 41467_2026_77225_MOESM12_ESM.pdf]

# nature portfolio

Corresponding author(s): Pascal D. Johann

Last updated by author(s): Aug 13, 2026

# Reporting Summary

Nature Portfolio wishes to improve the reproducibility of the work that we publish. This form provides structure for consistency and transparency in reporting. For further information on Nature Portfolio policies, see our Editorial Policies and the Editorial Policy Checklist.

## Statistics

For all statistical analyses, confirm that the following items are present in the figure legend, table legend, main text, or Methods section.

| n/a | Confirmed | |
|---|---|---|
| ☐ | ☒ | The exact sample size (*n*) for each experimental group/condition, given as a discrete number and unit of measurement |
| ☐ | ☒ | A statement on whether measurements were taken from distinct samples or whether the same sample was measured repeatedly |
| ☐ | ☒ | The statistical test(s) used AND whether they are one- or two-sided<br>*Only common tests should be described solely by name; describe more complex techniques in the Methods section.* |
| ☐ | ☒ | A description of all covariates tested |
| ☐ | ☒ | A description of any assumptions or corrections, such as tests of normality and adjustment for multiple comparisons |
| ☐ | ☒ | A full description of the statistical parameters including central tendency (e.g. means) or other basic estimates (e.g. regression coefficient) AND variation (e.g. standard deviation) or associated estimates of uncertainty (e.g. confidence intervals) |
| ☐ | ☒ | For null hypothesis testing, the test statistic (e.g. *F*, *t*, *r*) with confidence intervals, effect sizes, degrees of freedom and *P* value noted<br>*Give P values as exact values whenever suitable.* |
| ☒ | ☐ | For Bayesian analysis, information on the choice of priors and Markov chain Monte Carlo settings |
| ☒ | ☐ | For hierarchical and complex designs, identification of the appropriate level for tests and full reporting of outcomes |
| ☐ | ☒ | Estimates of effect sizes (e.g. Cohen's *d*, Pearson's *r*), indicating how they were calculated |

*Our web collection on statistics for biologists contains articles on many of the points above.*

## Software and code

Policy information about availability of computer code

| | |
|---|---|
| Data collection | Data collection was performed using a combination of manufacturer-provided software and standardized open-source pipelines. Bulk RNA microarray data were generated using Affymetrix GeneChip platforms and processed with manufacturer-recommended workflows. DNA methylation data were generated using Illumina Infinium HumanMethylation EPIC and EPIC v2 BeachChips, with raw IDAT files produced by Illumina scanner software. Whole-exome sequencing data were generated by Eurofins Genomics using Illumina Sequencing platforms; base calling and primary demultiplexing were performed using Illumina standard software. Single-nucleus RNA-Seq, single-nucleus ATAC-Seq, and single-nucleus multiome data were generated using the 10X Genomics Chromium platform, with raw sequencing data processed into FASTQ files using Cell Ranger mkfastq (v8.0.1). Spatial transcriptomics data were generated using the 10X Genomics Visium HD platform, with raw image and sequencing data processed using SpaceRanger (v3.1.3). No custom code was used during primary data acquisition. |

| | |
|---|---|
| Data analysis | R code is available via Zenodo (https://doi.org/10.5281/zenodo.21887084). Standard software packages and their versions used for all other analyses are described in the Methods. In brief: Data analysis was conduced using a combination of open-source and commercial software, primarily implemented in R (v4.2.0-v4.5.0) and PYthon (v3.10). Bulk transcriptomic analyses were performed using Bioconductor packages including limma, minfi, ConsensusClusterPlus, conumee2, and gimnet, as well as CRAN packages such as umap, pheatmap, ComplexHeatmap, EnhancedVolcano, survminer, timeROC, and rstatix. DNA methylation-based classification was implemented using custom R scripts following published frameworks and integrated into an interactive R Shiny application. Copy number analysis used conumee2, with downstream visualisation in IGV and GISTIC 2.0 via the GenePattern Cloud service.<br>Single-nucleus RNA-seq and multiome data were analysed using Seurat (v5.3.0), Signac, Harmony, Monocle 3, ChromVAR, cicero, scDb/Finder,LIANA, scMEGA, and enrichR, following published workflows. Spatial transcriptomics data were analysed in Python using bin2cell, scanpy,celltypist, and AnnData. Variant calling for whole-exome sequencing used multiple established tools including BWA-MEM, Picard, FreeBayes,GATK HaplotypeCaller, bcftools, Strelka, Platypus, and VarScan2, with annotation performed using VEP. Custom scripts were used to integratedatasets, perform subgroup-specific analyses, and generate figures, all based on standard, reproducible open-source workflows. |

For manuscripts utilizing custom algorithms or software that are central to the research but not yet described in published literature, software must be made available to editors and reviewers. We strongly encourage code deposition in a community repository (e.g. GitHub). See the Nature Portfolio guidelines for submitting code & software for further information.

# Data

Policy information about availability of data

All manuscripts must include a data availability statement. This statement should provide the following information, where applicable:

- Accession codes, unique identifiers, or web links for publicly available datasets
- A description of any restrictions on data availability
- For clinical datasets or third party data, please ensure that the statement adheres to our policy

Data generated in this study have been deposited in the Gene Expression Omnibus (GEO) under accession codes GSE308221 (DNA methylation, patient samples https://www.ncbi.nlm.nih.gov/geo/query/acc.cgi?acc=GSE308221), GSE343489 (Affymatrix Array, patient samples https://www.ncbi.nlm.nih.gov/geo/query/acc.cgi?acc=GSE343489), GSE316779 (Visium HD https://www.ncbi.nlm.nih.gov/geo/query/acc.cgi?acc=GSE316779), GSE336709 (DNA methylation, cell lines https://www.ncbi.nlm.nih.gov/geo/query/acc.cgi?acc=GSE336709), and GSE336546 (scRNA-seq, cell lines https://www.ncbi.nlm.nih.gov/geo/query/acc.cgi?acc=GSE336546).
Whole-exome sequencing data generated in this study are available under restricted access via the German Human Genome-Phenome Archive (GHGA) under accession https://data.ghga.de/study/GHGAS86868931177408. Single-nucleus sequencing data generated in this study are available under restricted access via GHGA under accession https://data.ghga.de/study/GHGAS91001004381672. Access to restricted-access data is limited to researchers with a bona fide scientific research purpose, in accordance with data protection regulations governing patient-derived genomic and clinical data under German and EU law (GDPR), which prohibit unrestricted public release of individual-level sequencing data. Access requires submission of a data access request to the study's Data Access Committee (DAC) via the GHGA data portal, including a description of the intended research use and confirmation of compliance with the approved Data Access Policy for the study. Requests are typically reviewed and answered within 2-4 weeks. Once granted, access remains valid for 12 months, renewable upon request. No dataset from this study has been separately deposited in the European Genome-phenome Archive (EGA); all restricted-access sequencing data generated in this study are held exclusively in GHGA.
The publicly available DNA-Methylation array data from Clay et al. 2019 used in this study are available in the GEO database under accession code GSE131350 is available with the following link https://www.ncbi.nlm.nih.gov/geo/query/acc.cgi?acc=GSE131350 [17]. The publicly available DNA-Methylation array data from Bueno et al. 2022 used in this study are available in the GEO database under accession code GSE179175 is available with the following link https://www.ncbi.nlm.nih.gov/geo/query/acc.cgi?acc=GSE179175 [18].

# Research involving human participants, their data, or biological material

Policy information about studies with human participants or human data. See also policy information about sex, gender (identity/presentation), and sexual orientation and race, ethnicity and racism.

| | |
|---|---|
| Reporting on sex and gender | Sex was recorded for all patients based on clinical records at the time of diagnosis and was reported as biological sex (female or male). Gender identity was not assessed in this study. Sex distribution differed across DNA methylation-defined subgroups and was therefore included in subgroup analyses. No participants were excluded based on sex, and analyses were not stratified by sex unless explicitly stated in the manuscript. |
| Reporting on race, ethnicity, or other socially relevant groupings | Information on race or ethnicity was not systematically collected for this study and was therefore not included in the other socially relevant analyses. The cohort comprises patients recruited through the German Malignant Endocrine Tumors (MET) Registry and groupings previously published international cohorts, including cohorts enriched for the Brazilian TP53 R337H founder mutation. Geographic origin and germline TP53 mutation status were considered where relevant to the scientific questions, but no analyses were performed based on self-reported race or ethnicity. |
| Population characteristics | The study population consisted of pediatric and adolescent patients diagnosed with adrenocortical tumors, including adrenocortical carcinomas, adenomas, and tumors of uncertain malignant potential. Patients were diagnosed between 1997and 2024. The combined cohort included both sexes, with a female predominance, and spanned early childhood through adolescence. Clinical characteristics including age at diagnosis, tumor stage, histopathological classification, endocrine presentation, germline TP53 status, and clinical outcome were recorded and analyzed as described in the manuscript. Healthy adrenal cortex samples from pediatric and adult donors served as controls where indicated. |
| Recruitment | Patients were recruited retrospectively and prospectively through the German Malignant Endocrine Tumors (MET) Registry and through inclusion of previously published cohorts. Eligible patients were identified based on a confirmed diagnosis of a pediatric adrenocortical tumor and availability of tumor material. Written informed consent for participation and use of clinical data and biospecimens was obtained from patients and/or legal guardians at the time of registry enrollment, in accordance with local and national regulations. Recruitment was not influenced by sex, disease severity, or outcome. No |

| | |
|---|---|
| | financial or other incentives were provided for participation. |
| Ethics oversight | Patient Material, clinical data and written consent from all included patients (n=109) were obtained from the German MET-Registry. The MET studies are conducted in accordance with the Declaration of Helsinki and were approved by the ethics committees of the University of Luebeck (IRB 97125) and Otto-von-Guericke-University Magdeburg (IRBs 174/12 and 52/22),Germany. Written informed consent including data and bio-specimen usage in scientific projects was obtained from all patients and/or legal guardians at the time of registry enrollment, in accordance with national regulations. The MoPACT project (IRB 23-0351) and the EpiGenPAT project (IRB 21-0997) were approved by the Ethics Committee of the Ludwig Maximilians University Munich, Germany. |

Note that full information on the approval of the study protocol must also be provided in the manuscript.

# Field-specific reporting

Please select the one below that is the best fit for your research. If you are not sure, read the appropriate sections before making your selection.

☒ Life sciences ☐ Behavioural & social sciences ☐ Ecological, evolutionary & environmental sciences

For a reference copy of the document with all sections, see nature.com/documents/nr-reporting-summary-flat.pdf

# Life sciences study design

All studies must disclose on these points even when the disclosure is negative.

| | |
|---|---|
| Sample size | No statistical methods were used to predetermine sample size. Sample sizes were determined by the availability of patient material meeting inclusion criteria within the German Malignant Endocrine Tumors (MET) Registry and by the availability of suitable samples from previously published cohorts. For functional and single-cell experiments, sample sizes were based on established standards in the field and material availability. Exact sample sizes for each experiment and analysis are reported in the manuscript, figures, and figure legends. |
| Data exclusions | No data were excluded from the analyses unless explicitly stated. For sequencing-based analyses, standard quality control criteria were applied as described in the Methods, including filtering of low-quality nuclei or cells, doublets, and features with insufficient counts. These exclusions were based on predefined, objective quality thresholds and were applied uniformly across samples, No data points were excluded post hoc to influence outcomes. |
| Replication | Key findings were supported by multiple levels of replication. Single-nucleus RNA-seq and multiome analyses were performed on multiple independent tumor samples representing all molecular subgroups, with consistent results. Functional experiments were conducted in multiple independent cell lines and biological replicates, yielding reproducible effects. Replication details and numbers of independent experiments are provided in the Methods and figure legends. |
| Randomization | Randomization was not applicable to this study. This was an observational study of patient-derived samples, and sample allocation to experimental groups was determined by biological characteristics such as molecular subgroup, treatment condition, or sample availability rather than random assignment. |
| Blinding | Blinding was applied for histopathological assessment. Evaluation of CTNNB1 (B-catenin) immunohistochemistry was performed by a pathologist who was blinded to DNA methylation-based subgroup assignment and clinical outcome. Molecular and computational analyses were conducted using predefined, automated pipelines and were not blinded. No additional blinding was applied during data collection or analysis. |

# Reporting for specific materials, systems and methods

We require information from authors about some types of materials, experimental systems and methods used in many studies. Here, indicate whether each material, system or method listed is relevant to your study. If you are not sure if a list item applies to your research, read the appropriate section before selecting a response.

## Materials & experimental systems

| n/a | Involved in the study |
|---|---|
| ☐ | ☒ Antibodies |
| ☐ | ☒ Eukaryotic cell lines |
| ☒ | ☐ Palaeontology and archaeology |
| ☒ | ☐ Animals and other organisms |
| ☐ | ☒ Clinical data |
| ☒ | ☐ Dual use research of concern |
| ☒ | ☐ Plants |

## Methods

| n/a | Involved in the study |
|---|---|
| ☒ | ☐ ChIP-seq |
| ☒ | ☐ Flow cytometry |
| ☒ | ☐ MRI-based neuroimaging |

## Antibodies

| | |
|---|---|
| Antibodies used | Immunohistochemistry (Figure 4F): |

| | |
|---|---|
| | Staining for β-catenin (CTNNB1) was performed on formalin-fixed, paraffin-embedded sections using a mouse monoclonal antibody (Cell Marque, Ref: 224M-15) at a 1:50 dilution. |
| Validation | Validation of hte antibody was performed by the manufacturer |

## Eukaryotic cell lines

Policy information about cell lines and Sex and Gender in Research

| | |
|---|---|
| Cell line source(s) | Human adrenocortical carcinoma cell lines were used in this study. CU-ACC1 was kindly provided by Katja Kiseljak-Vassiliades (University of Colorado, USA). The H295R cell line was purchased from Cytion. Both cell lines originate from female patients with adrenocortical carcinoma. |
| Authentication | Cell line identity was confirmed based on documentation provided by the original source (CU-ACC1) or the commercial supplier (H295R from Cytion) and on consistency of molecular and phenotypic characteristics with previously published descriptions, including characteristic copy number alterations as seen by DNA methylation profiling. |
| Mycoplasma contamination | Cell lines were routinely tested for mycoplasma contamination and were confirmed to be mycoplasma-negative prior to and during experimental use. |
| Commonly misidentified lines (See ICLAC register) | The cell lines used in this study are not listed in the International Cell Line Authentication Committee (ICLAC) database of commonly misidentified or cross-contaminated cell lines. |

## Clinical data

Policy information about clinical studies

All manuscripts should comply with the ICMJE guidelines for publication of clinical research and a completed CONSORT checklist must be included with all submissions.

| | |
|---|---|
| Clinical trial registration | This study was not a clinical trial and was therefore not registered. It is a retrospective and prospective observational study based on patient samples and clinical data obtained from the German Malignant Endocrine Tumors (MET) Registry and previously published cohorts. |
| Study protocol | *Note where the full trial protocol can be accessed OR if not available, explain why.* |
| Data collection | Clinical data were collected through the German Malignant Endocrine Tumors (MET) Registry and collaborating centers, Collected variables included age at diagnosis, sex, tumor stage, histopathological classification, endocrine presentation, germline TP53 status, treatment information where available, and clinical outcome. Data were obtained from medical records and registry documentation at the time of diagnosis and during follow-up. Additional molecular and clinical data from previously published cohorts were integrated for comparative analyses as described in the manuscript. |
| Outcomes | The primary clinical outcome was overall survival, defined as the time from diagnosis to death from disease or last follow-up. Secondary outcomes included five-year overall survival and median survival. Outcomes were analyzed in relation to DNA methylation —based molecular subgroups and established clinical and histopathological risk stratification systems, as detailed in the Methods. |

## Plants

| | |
|---|---|
| Seed stocks | *Report on the source of all seed stocks or other plant material used. If applicable, state the seed stock centre and catalogue number. If plant specimens were collected from the field, describe the collection location, date and sampling procedures.* |
| Novel plant genotypes | *Describe the methods by which all novel plant genotypes were produced. This includes those generated by transgenic approaches, gene editing, chemical/radiation-based mutagenesis and hybridization. For transgenic lines, describe the transformation method, the number of independent lines analyzed and the generation upon which experiments were performed. For gene-edited lines, describe the editor used, the endogenous sequence targeted for editing, the targeting guide RNA sequence (if applicable) and how the editor was applied.* |
| Authentication | *Describe any authentication procedures for each seed stock used or novel genotype generated. Describe any experiments used to assess the effect of a mutation and, where applicable, how potential secondary effects (e.g. second site T-DNA insertions, mosiacism, off-target gene editing) were examined.* |
