# Supplementary material for: CpG hypermethylation and WNT/AP-1 cooperativity define the epigenetic landscape and a clinical subgroup of high-risk pediatric adrenocortical carcinoma
Source: Nat Commun. 2026 Sep 14;17:9786. doi: 10.1038/s41467-026-77225-5 (PMC13575229; doi:10.1038/s41467-026-77225-5)
Supplement: Supplementary file 2 [file 41467_2026_77225_MOESM13_ESM.pdf]

nature portfolio

Peer Review File

CpG Hypermethylation and WNT/AP-1 cooperativity Define the Epigenetic Landscape and a Clinical Subgroup of High-Risk Pediatric Adrenocortical Carcinoma

Corresponding Author: Professor Pascal Johann

Parts of this Peer Review File have been redacted as indicated to remove third-party material.

This file contains all reviewer reports in order by version, followed by all author rebuttals in order by version.

Version 0:

Reviewer comments:

Reviewer #1

(Remarks to the Author)
Fincke, Lobner et al. perform a large multi-omic analysis of pediatric adrenocortical tumors uncovering a high-risk group that is defined by WNT activation and AP-1. Overall, the omic data presented in this study are very robust and provides new and more definitive results on the high-risk pediatric ACC subgroup that has been previously identified. Moreover, the single cell RNA-seq and ATAC data (and spatial data) provide more in-depth analysis of the molecular features of this high-risk group. While WNT is well-known to be an important molecular feature of aggressive ACC, the AP-1 pathway identified is novel. However, the work could be significantly improved by further analysis and experiments to more clearly define the AP-1 aspect of the high-risk group. Additionally, the experiments with HDAC inhibition are not well founded.

Major comments:
1. Regarding the AP-1 pathway, the authors discover AP-1 pathway in the HR group from pseudotime analysis integrating snATAC and RNA-seq in Figure 6D. The authors then show motif enrichment analysis comparing the subgroups in Figure 6E for TCG7L2. Could they also show a similar plot with some of the AP-1 complex genes?
2. Spatial transcriptomic data shows elevated WNT pathway activity in HR tumors but what about AP-1 activity? Data regarding AP-1 pathway should be shown and discussed (even not positive). Also, the genes included in the WNT score should be reported to the reader.
3. Since the authors purport that the adult ACC cell lines H295R and CU-ACC1 are representative of HR pediatric ACC, can they show AP-1 pathway activity in these cell lines either by RNA-seq, western blotting or both? For example, expression of c-Jun and/or c-Fos, ERK and/or p38. These data would be very interesting to the reader as this pathway is new in ACC (whereas WNT signaling is well known).
4. Previous large scale drug screening results in the same ACC cell lines used by the authors did not find entinostat (or other HDAC inhibitors) to be cytotoxic (IC50 between 3-5 uM) (see supplementary table 1, PMID: 38451783). These data should be discussed. More importantly, as the authors do not have any in vivo data supporting HDAC inhibition as an anti-tumor therapy, the data suggesting HDAC inhibition as cytotoxic therapy in the results and discussion section is not appropriate. Instead, they should discuss HDAC inhibition more in the context of their single cell data and as more of tool to understand the biology of the HR pediatric group. The methylation data is also problematic as noted in the next comment.
5. While I understand that the authors focus on HDAC inhibition because it may be more effective modulating ACC cell growth than DNA demethylation agents, it is unclear why they use HDAC inhibition as a way to reduce promoter associated DNA methylation as noted in lines 460-462? HDAC inhibitors do not inhibit DNMT enzymes or target CpG islands specifically. Therefore, whatever effect entinostat is having on DNA methylation is indirect. Typically, HDAC inhibitors are known to alter H3K27Ac which the authors did not assess. Since the focus of the paper is on DNA methylation in the high risk group, the use of HDAC inhibition based on viability as noted in the comment above and the use of HDAC inhibition as a DNA methylation modulator are not supported. In fact, it would be more interesting to see if decitabine (which has no cytotoxic effect as shown in Figure 6G) would alter the promoter-associated CpG sites as is shown for entinostat in Figure 6H and AP-1 sites as shown in Figure 6J.
6. In addition, authors should validate their single cell and methylation findings with HDAC inhibition by orthogonal methods in the H295R and CU-ACC1 cell lines. For example, lines 475-480 they focus on downregulation of oxphos gens and

steroidogenic genes after entinostat. Some of these markers should be by checked by PCR and/or Western blotting. Similarly, in lines 481-484 the authors mention pro-apoptotic gene upregulation. Apoptosis should be checked by flow cytometry, i.e. annexin staining and/or Western blotting.

Minor comments:
1. Reviewer token for GEO raw data is not available and must be provided to ensure all sequencing data has been appropriately uploaded.
2. The supplemental methods are not available in the Reviewer documents. Full methods should be included in the main manuscript.
3. Full drug sensitivity results from the pediatric tumor should be included as a supplementary table
4. It was very difficult to follow all the supplementary figure as they are uploaded individually and many are not properly labelled. All supplementary figures should be in one file and clearly labelled.

Reviewer #2

(Remarks to the Author)
The manuscript entitled “CpG Hypermethylation 1 and WNT/AP-1 cooperativity Define the Epigenetic Landscape and a Clinical Subgroup of High-Risk Pediatric Adrenocortical Carcinoma” presents a comprehensive molecular analysis on pediatric adrenocortical carcinoma (pACTs) using data generated from DNA methylation profiling, transcriptomics, chromatin accessibility and spatial transcriptomics. By integrating methylation data generated from the study cohort with two publicly available cohorts, the authors defined four subgroups of pACT based on methylation profile, one of which is a high-risk group exhibiting hypermethylation, chromosomal instability. Single-cell analyses showed that the high-risk group has balanced representation of zona glomerulosa (zG), reticularis (zR) and fasciculata (zF) cells, exhibiting transcriptional co-activation of WNT and AP-1 signaling programs. Treatment of HDAC inhibitor entinostat in patient cell culture and cell line models demonstrated efficacy via reversal of promoter methylation, silencing of AP-1 activity, and inducing apoptotic reprogramming.
This multi-omics, tour de force study has revealed major insights into the molecular features associated with high-risk pACT, which can impact risk stratification and future translational research on targeting high-risk pACT.
Several comments are listed below for authors’ consideration.
Major comments:
1) The four methylation groups are labeled as low risk 1-3 and high risk from the very beginning, which can cause confusion by assuming that these four groups were stratified based on clinical outcome. It would be better to use a generic label (e.g. A, B, C and D) to show that the initial grouping was based on methylation profile but not clinical risk. Additionally, Umap of methylation data (Figure 1C) shows that the separation of these four groups is not distinct. The authors need to explain why there are some admixture of these four groups in Umap.
2) It will be good to use the expression data to confirm that CNV events enriched in HR tumors are accompanied by expected expression change, e.g. amplification of MYC, CDK4, MDM2, and AURKC and deletion of BRCA2, CHEK2 and TP73.
3) Integrative analysis of methylation status from bulk samples with heterogenous cellular compositions defined by snRNA-seq should be considered. It was striking to see that the HR samples have much higher CpG methylation state compared with the low-risk ones (a median CpG methylation level of 0.62 compared to 0.2), which suggests suppression of gene expression in high-risk samples. On the other hand, the transcriptional diversity of HR samples suggests more active transcriptional program. The authors need to perform an integrative analysis using samples that have both methylation data and snRNA-seq to show whether the methylation status is correlated with tumor snRNA-seq clusters or with the tumor microenvironment.
4) Figure 4A is difficult to interpret. It is stated as a heatmap of pairwise correlations among seven transcriptional metasignatures. However, the number of samples exceeds the 18 used for snRNA profiling and it is unclear why the metasignature counts differ across the samples in second row. More explanation is required. The color scale of Figure 4B is too close to distinguish the different signatures especially those described in the main text (e.g. Cell Cycle Activation).
5) The description of the methylation classifier is very brief and more detailed information (i.e. software packages used, version, parameter), training and validation data set used, should be included. This issue applies to all data analysis methods which should be expanded to ensure reproducibility of the results.

Minor comments
1) Supplementary Figure 1E: it will be good to provide the description of clinical features so that readers who are not familiar with clinical features of adrenocortical tumor can understand this graph. The narrative accompanying this figure panel at lines 168-174 also lacks the description of these clinical features. Similarly, the importance of Ki-67 proliferation index and MKI67 transcript levels and the data source needs to be introduced.
2) Line 141-144: Figure S2 shows that risk stratification based on methylation grouping was significant in pACC but not in the other two diagnostic groups (i.e. UMP and ACA). The current writing, “The added value of the subgroups, considering the prognostic positive pACA group only, is limited (Supplementary Figure S2).”, reads quite awkward and needs to be clarified.
3) The reclassification of 15 Clay A1 tumors only showed 7 were assigned to the high-risk group. The authors mentioned that 7 out of 8 reclassified A1 tumors survived the disease (line 158-160). Can the authors demonstrate that the risk stratification is improved over the original Clay grouping by presenting a survival curve for the Clay cohort using these two classification schemes?
4) Figure 2F shows pie-charts for CTNNB1 mutation frequency in each subgroup. It will be better to use bar plots (one bar for each subgroup) for showing the frequency of all key driver genes in these four subgroups with the significance for

enrichment in a particular group labeled.
5) Figure 3B which introduces the samples used for snRNA-seq should be placed as the first panel (Fig. 3A) instead. Figure 3C legend: clusters are labeled as zR zF instead of zF zR as in the other parts of the manuscript. Cluster labels in Figure 3C and 3F are inconsistent which should be corrected.
6) The finding of “Regulatory Motif Analysis Suggests CTNNB1- and AP1-binding motifs as Drivers of Tumor Biology in High Risk pACTs” was based on the scATAC-seq data generated from the reproducibility analysis of the preceding section and it would be good to provide a background when introducing this analysis.
7) Line 101: “his work” should be “this work”?
8) Line 391: “dedifferentiated zG-like cells” should be “undifferentiated zG-like cells”?

Reviewer #3

(Remarks to the Author)
The manuscript by Fincke et al. presents a multi-omic analysis—including methylation, bulk transcriptome, exome, single-cell RNA-seq, ATAC-seq, and spatial transcriptomics—of pediatric adrenocortical tumors. This original and unique dataset for these rare pathologies identifies, through methylome profiling, a subgroup of tumors associated with poor prognosis (the high-risk, HR, group), characterized by CpG island hypermethylation and concomitant activation of the Wnt and AP-1 pathways. In addition, the authors show in a preclinical study that this tumor subgroup may be sensitive to HDAC inhibitors, opening new therapeutic perspectives for these rare pediatric tumors.
Overall, the datasets generated in this study represent an important resource for the scientific community and provide new insights into the biology of pediatric adrenocortical tumors that could ultimately improve patient management. However, several points require clarification or further detail, mainly regarding the analytical methods and, to a lesser extent, some of the results.

Major points

1- The Methods section is insufficiently detailed and does not allow reproduction of the analyses or proper assessment of their scientific validity. Although the authors refer to Supplementary Materials containing detailed methods, this file was not provided.
For instance, the authors developed a random forest model to classify new patients based on methylation data, but it is unclear how batch effects from independent methylation series would be handled, how the nested cross-validation was performed (number of folds, stratification by risk group), or how classifier performance was assessed (e.g., average performance across outer folds).
Similarly, the exome analysis is described only briefly: “Tumor-normal paired WES was performed at 50× coverage using Agilent SureSelect V8 capture. Variants were called using six algorithms, annotated with VEP, and filtered against population databases.” The alignment algorithm and reference genome are not specified, the six variant-calling tools are not listed, and the annotation process is not described. VEP should be defined, and details should be provided regarding population frequency thresholds, databases used for filtering, and any applied quality, allelic fraction, or functional consequence filters.
This lack of methodological transparency is particularly problematic given that the analysis code is not made available to the community.
Similar concerns apply to the entire Methods section.
In addition, the sample size (N) for each analysis, including bulk transcriptome and exome analyses, should be clearly stated.

2- Regarding the exome analysis, a table summarizing the identified variants is missing. At minimum, somatic variants should be reported, especially if germline variants cannot be described for regulatory reasons.
In particular, the reported 10% frequency of somatic ARMC5 variants is surprising, as this gene is classically described as constitutively altered in bilateral macronodular adrenal disease.
Moreover, providing tumor mutational burden (TMB) values across the different molecular or risk groups would add useful contextual information.

3- For the single-cell RNA-seq and spatial transcriptomics analyses, the number of ACC, UMP, and ACA tumors included should be specified to facilitate interpretation. It would also improve clarity to label tumors according to their histological type (ACC, ACA, or UMP) rather than using the generic term ACT in Figures 3-4.
The single-cell analysis provides a clear and interesting description of steroidogenic cell heterogeneity, and these findings partly overlap with those reported in adult single-cell atlases (PMID: 38759836, 39167619, 41053133). Discussing similarities and differences with adult tumors would strengthen the manuscript.
In addition, differences in the tumor microenvironment are likely, as recently reported in adult adrenocortical tumors (PMID: 41053133). A dedicated analysis of immune and stromal compartments could help characterize cell types and cellular states and evaluate how they associate with the defined risk groups.
For the spatial analyses, the dispersion of cell populations could be quantitatively assessed using spatial autocorrelation metrics, such as Moran’s I, rather than being described solely by visual inspection.

4- The results presented in this study could potentially be leveraged to propose a clinically useful decision tree for patient stratification, for example by sequentially considering histological subtype (ACC versus UMP), age (> 5 years?), CTNNB1

immunohistochemistry status, and methylation-based risk group.

Minor points / Typos
- Lines 132–135: reporting survival values consistently as percentages rather than proportions would improve clarity, as "HR: 0.25" may be misinterpreted as a hazard ratio rather than a 5-year survival rate.
- Line 191: "Fig. S5" should be cited instead of "Fig. 5".
- Lines 258–259: it would be helpful to specify whether more ZR-specific steroidogenic genes, such as CYB5A, CYB5B, or SULT2A1, were examined.
- Line 331: "Figure 4E" should be cited instead of "Figure 5E".
- Figure 4: risk groups should be indicated as clearly as possible in all panels.
- Supplementary Figure S11C: labels are misaligned and overlap the panel.
- Supplementary Figure S14: histological characterization is missing (Supplementary Figure S16 appears to be provided twice).
- Supplementary Figure S15: the meaning of the red vertical dashed line should be clarified.
- Supplementary Table 1: the title of column D is missing, and the title of column W is unclear.

Version 1:

Reviewer comments:

Reviewer #1

(Remarks to the Author)
Overall, the authors responded well to reviewer comments. Still there are a few remaining issues:

1. Supplementary Figure S15 shows no major differences in AP-1 binding motifs in HR compared to LR1. This should be explicitly mentioned in the text even though I understand that authors emphasize that HR has both WNT and AP-1 activity compared to LR1 which has only AP-1 activity.
2. It's good that the authors added Supplementary Figure S17 but it's uninterpretable to the reader. There needs to be some summary statistics of each spatial panel.
3. The AP-1 qRT-PCR data is a good addition but the legend descriptions for Figure 6 are completely off and do not match the data. Authors need to fix this.
4. Access to genomic datasets: the GHCA link does not work, this needs to be corrected
5. DNA methylation and single cell RNA-seq data from H295R and CU-ACC1 cells with and without drug treatments needs to be uploaded to GEO

Reviewer #2

(Remarks to the Author)
The authors have addressed all the issues raised in the previous review. There is only one minor problem: Figure 1 legend appears to have swapped the content of panel D with panel E.

Reviewer #3

(Remarks to the Author)
The authors have addressed all my comments and questions satisfactorily. The revisions have improved the manuscript, and I have no further concerns.

Open Access This Peer Review File is licensed under a Creative Commons Attribution 4.0 International License, which permits use, sharing, adaptation, distribution and reproduction in any medium or format, as long as you give appropriate credit to the original author(s) and the source, provide a link to the Creative Commons license, and indicate if changes were made.
In cases where reviewers are anonymous, credit should be given to 'Anonymous Referee' and the source.
The images or other third party material in this Peer Review File are included in the article's Creative Commons license, unless indicated otherwise in a credit line to the material. If material is not included in the article's Creative Commons license and your intended use is not permitted by statutory regulation or exceeds the permitted use, you will need to obtain permission directly from the copyright holder.
To view a copy of this license, visit https://creativecommons.org/licenses/by/4.0/

REVIEWER COMMENTS

Reviewer #1, expertise in adrenocortical carcinoma (Remarks to the Author):

Fincke, Lobner et al. perform a large multi-omic analysis of pediatric adrenocortical tumors uncovering a high-risk group that is defined by WNT activation and AP-1. Overall, the omic data presented in this study are very robust and provides new and more definitive results on the high-risk pediatric ACC subgroup that has been previously identified. Moreover, the single cell RNA-seq and ATAC data (and spatial data) provide more in-depth analysis of the molecular features of this high-risk group. While WNT is well-known to be an important molecular feature of aggressive ACC, the AP-1 pathway identified is novel. However, the work could be significantly improved by further analysis and experiments to more clearly define the AP-1 aspect of the high-risk group. Additionally, the experiments with HDAC inhibition are not well founded.

> Authors: We thank Reviewer 1 for the thorough and positive evaluation of our manuscript and for the assessment of the multi-omic data as well as the novelty of the AP-1 pathway activation. We have carefully addressed all specific concerns raised, as detailed point by point below.

Major comments:

1. Regarding the AP-1 pathway, the authors discover AP-1 pathway in the HR group from pseudotime analysis integrating snATAC and RNA-seq in Figure 6D. The authors then show motif enrichment analysis comparing the subgroups in Figure 6E for TCG7L2. Could they also show a similar plot with some of the AP-1 complex genes?

> Authors: We thank the reviewer for this suggestion. This has been present in the previous version (former Supplementary Figure 15D). The results section explicitly describes a motif enrichment analysis of FOS::JUND binding sites across risk groups and references Supplementary Figure S15D, which displays the corresponding enrichment plot (in analogoy to the TCF7L2 analysis in Figure 6E). To place this aspect more prominently, it can now be found in S15D in the figure legend of Figure 5E („Equivalent figure for AP-1 is displayed in Supplementary Figure S15D“).

2. Spatial transcriptomic data shows elevated WNT pathway activity in HR tumors but what about AP-1 activity? Data regarding AP-1 pathway should be shown and discussed (even not positive). Also, the genes included in the WNT score should be reported to the reader.

> Authors: We thank the reviewer for this constructive point. Regarding AP-1 pathway activity in the spatial transcriptomics data, we have included a spatial visualization of AP-1 activity scores across tumor sections in Supplementary Figure S17, alongside

the WNT pathway data shown in the main figure. The spatial AP-1 signal was less pronounced and more heterogeneous than the WNT pathway enrichment, and we have added a brief statement in the manuscript acknowledging this observation (ll. 518ff.) and noting that the spatial resolution of AP-1 activity may be limited by the diffuse and context-dependent nature of AP-1 transcriptional programs, which are better captured at single-cell resolution as shown in our snRNA-seq and snATAC-seq analyses. Regarding the WNT score gene composition a reference to the PROGENy-derived WNT pathway signature has been added to the relevant figure legend and results text (ll. 521f. and Supplementary Methods ll. 363ff.).

3. Since the authors purport that the adult ACC cell lines H295R and CU-ACC1 are representative of HR pediatric ACC, can they show AP-1 pathway activity in these cell lines either by RNA-seq, western blotting or both? For example, expression of c-Jun and/or c-Fos, ERK and/or p38. These data would be very interesting to the reader as this pathway is new in ACC (whereas WNT signaling is well known).

Authors: We thank the reviewer for this important suggestion and agree that directly demonstrating AP-1 pathway activity in the cell lines used strengthens the translational relevance of our findings.

To address this point, we performed qRT-PCR following serum starvation and re-stimulation, a widely used paradigm to induce immediate-early/AP-1 target genes (PMID: 40280716, PMID: 20466732), in both H295R and CU-ACC1 cells to assess the transcriptional responsiveness of AP-1 components. *FOS*, *FOSB*, *JUN*, and *JUNB* were rapidly and robustly induced in both cell lines, with induction detectable as early as 15 minutes and peaking up to 132-fold for *FOSB* at 120 minutes in H295R cells (one-way ANOVA with Tukey HSD, all $p < 0.001$; new Figure 6J). These results confirm that the AP-1 transcriptional program is active and stimulus-responsive in both adult ACC cell lines, directly supporting their use as models for the AP-1-driven program we identified in HR-pACT. The manuscript has been updated accordingly (see Resultsl ll. 576 ff.) and new Figure 6J).

4. Previous large scale drug screening results in the same ACC cell lines used by the authors did not find entinostat (or other HDAC inhibitors) to be cytotoxic (IC50 between 3-5 uM) (see supplementary table 1, PMID: 38451783). These data should be discussed. More importantly, as the authors do not have any in vivo data supporting HDAC inhibition as an anti-tumor therapy, the data suggesting HDAC inhibition as cytotoxic therapy in the results and discussion section is not appropriate. Instead, they should discuss HDAC inhibition more in the context of their single cell data and as more of tool to understand the biology of the HR pediatric group. The methylation data is also problematic as noted in the next comment.

Authors: We thank the reviewer for this well-founded critique. We agree that the original framing overstated the therapeutic implications of our HDAC inhibition data, particularly in the absence of in vivo validation. We have revised this section of the Discussion to reframe entinostat treatment as a mechanistic tool used to probe the epigenetic dependencies of the HR group, rather than as a candidate for direct therapeutic translation. We explicitly acknowledge the prior large-scale drug screening data reported by the reviewer indicating limited cytotoxic potency (ll. 665). Our revised discussion now focuses on the transcriptional reprogramming induced by entinostat, specifically the partial restoration of endocrine differentiation programs, as evidence that the epigenetic state of HR pACTs is biologically reversible, and frames this as a mechanistic observation rather than a therapeutic conclusion („Consistent with this, our data should be interpreted primarily in a biological rather than a therapeutic context.“, l. 667f.). We agree that dedicated *in vivo* studies will be required before any therapeutic claims can be made, and have stated this explicitly in the revised text (ll. 677ff.).

5. While I understand that the authors focus on HDAC inhibition because it may be more effective modulating ACC cell growth than DNA demethylation agents, it is unclear why they use HDAC inhibition as a way to reduce promoter associated DNA methylation as noted in lines 460-462? HDAC inhibitors do not inhibit DNMT enzymes or target CpG islands specifically. Therefore, whatever effect entinostat is having on DNA methylation is indirect. Typically, HDAC inhibitors are known to alter H3K27Ac which the authors did not assess. Since the focus of the paper is on DNA methylation in the high risk group, the use of HDAC inhibition based on viability as noted in the comment above and the use of HDAC inhibition as a DNA methylation modulator are not supported. In fact, it would be more interesting to see if decitabine (which has no cytotoxic effect as shown in Figure 6G) would alter the promoter-associated CpG sites as is shown for entinostat in Figure 6H and AP-1 sites as shown in Figure 6J.

Authors: We thank the reviewer for this important mechanistic critique and agree that the original framing was imprecise. We have revised the relevant text to clarify that any effect of entinostat on DNA methylation is indirect, likely mediated through chromatin remodeling rather than direct DNMT inhibition, and have removed any implication of a direct mechanistic link between HDAC inhibition and CpG methylation changes. However – although not yet proven- in the the context of adrenocortical tumors – knock down of HDAC enzymes has been shown to reduce DNMT1 expression in other tumors, thus providing a possible albeit indirect explanation for methylation reduction (Jia et al., 2017; Schäfer et al., 2014). Following the reviewer's suggestion, we have included 72-hour 0.5 µM decitabine-treated methylation data in the revised Figure 6G. Decitabine treatment resulted in comparable downregulation of methylation.

6. In addition, authors should validate their single cell and methylation findings with HDAC inhibition by orthogonal methods in the H295R and CU-ACC1 cell lines. For example, lines 475-480 they focus on downregulation of oxphos gens and steroidogenic genes after entinostat. Some of these markers should be by checked by PCR and/or Western blotting. Similarly, in lines 481-484 the authors mention pro-apoptotic gene upregulation. Apoptosis should be checked by flow cytometry, i.e. annexin staining and/or Western blotting.

Authors: We thank the reviewer for this suggestion and have addressed both points experimentally.
We performed qRT-PCR for *STAR, CYP11A1, CYP21A2*, and *FDX1* in entinostat-treated H295R and CU-ACC1 cells. While the extent of the response was gene- and cell-line-dependent, we obesvered significant changes for *STAR* and *CYP11A1* in H295R and for *CYP21A2* and *STAR* in CU-ACC1 (Tukey HSD, $p < 0.05$; new Supplementary Figure S20), all of which are key genes involved in steroidogenesis. Overall this suggests that the scRNA-seq signal reflects broad tumour-level reprogramming.
Annexin V / 7AAD cytometry confirmed an increase in apoptotic cells in both H295R and CU-ACC1 following entinostat treatment (new Supplementary Figure S21C), directly validating the pro-apoptotic gene expression signature identified in the scRNA-seq data.

Minor comments:
1. Reviewer token for GEO raw data is not available and must be provided to ensure all sequencing data has been appropriately uploaded.
2. The supplemental methods are not available in the Reviewer documents. Full methods should be included in the main manuscript.
3. Full drug sensitivity results from the pediatric tumor should be included as a supplementary table
4. It was very difficult to follow all the supplementary figure as they are uploaded individually and many are not properly labelled. All supplementary figures should be in one file and clearly labelled.

Authors: We thank the reviewer for these important practical points and apologize for the inconvenience caused by these oversights in the initial submission. We have addressed all four points in the revised submission. The GEO reviewer token granting access to the raw sequencing data has been in the revised manuscript to allow full data review prior to publication where possible (DNA methylation data: GSE308221, reviewer token: svezckkqxnitxgf, Visium HD: GSE316779, reviewer token:

uvuzyeaqfhylrqn). The Supplementary Materials and Methods, which were inadvertently omitted from the initial submission, have now been included as a dedicated supplementary file. All drug sensitivity results from the pediatric tumor have been compiled and added as a new supplementary table S8 with key features such as IC25, IC50 and IC75 for each of the drugs tested. Finally, all supplementary figures should have been consolidated into a single file with consistent figure numbering throughout, to facilitate navigation during review.

Reviewer #2, expertise in pediatric adrenocortical carcinoma omics (Remarks to the Author):

The manuscript entitled “CpG Hypermethylation 1 and WNT/AP-1 cooperativity Define the Epigenetic Landscape and a Clinical Subgroup of High-Risk Pediatric Adrenocortical Carcinoma” presents a comprehensive molecular analysis on pediatric adrenocortical carcinoma (pACTs) using data generated from DNA methylation profiling, transcriptomics, chromatin accessibility and spatial transcriptomics. By integrating methylation data generated from the study cohort with two publicly available cohorts, the authors defined four subgroups of pACT based on methylation profile, one of which is a high-risk group exhibiting hypermethylation, chromosomal instability. Single-cell analyses showed that the high-risk group has balanced representation of zona glomerulosa (zG), reticularis (zR) and fasciculata (zF) cells, exhibiting transcriptional co-activation of WNT and AP-1 signaling programs. Treatment of HDAC inhibitor entinostat in patient cell culture and cell line models demonstrated efficacy via reversal of promoter methylation, silencing of AP-1 activity, and inducing apoptotic reprogramming.
This multi-omics, tour de force study has revealed major insights into the molecular features associated with high-risk pACT, which can impact risk stratification and future translational research on targeting high-risk pACT.
Several comments are listed below for authors’ consideration.

Authors: We thank Reviewer 3 for the thorough and positive evaluation of our manuscript and for the recognition of the multi-omic approach and its potential impact on risk stratification and translational research in pACT. We have carefully addressed all specific comments raised, as detailed point by point below.

Major comments:
1) The four methylation groups are labeled as low risk 1-3 and high risk from the very beginning, which can cause confusion by assuming that these four groups were stratified based on clinical outcome. It would be better to use a generic label (e.g. A, B, C and D) to show that the initial grouping was based on methylation profile but not clinical risk. Additionally, Umap of methylation data (Figure 1C) shows that the separation of these four

groups is not distinct. The authors need to explain why there are some admixture of these four groups in Umap.

Authors: We thank the reviewer for this thoughtful comment. Regarding the admixture observed in the UMAP of methylation data (Figure 1C), we would like to note that UMAP is a dimensionality reduction technique optimized for visualization purposes and does not always perfectly recapitulate the cluster boundaries defined by the underlying classifier. The partial overlap visible in the UMAP reflects the continuous nature of epigenetic variation across subgroups rather than misclassification, and the subgroup assignments are based on the full high-dimensional methylation profile rather than the two-dimensional UMAP projection. We have added a brief explanatory note to the figure legend to clarify this point. Regarding the subgroup nomenclature, we respectfully prefer to retain the designations "Low Risk 1–3" and "High Risk" rather than adopting generic labels such as A–D. While we appreciate the concern that risk-based naming might imply clinical outcome-driven stratification, we would like to clarify that these labels were assigned based on the methylation profiles in conjunction with the clinical outcome data available for our cohort, and we believe the risk-informed nomenclature more accurately reflects the clinical relevance of the subgroups as characterized in this study. Using generic labels would, in our view, obscure this clinically meaningful association and reduce the interpretability of the findings for the reader. To avoid any potential confusion, we have added a clarifying statement in the manuscript explicitly noting that the initial grouping was derived from unsupervised methylation profiling, with risk designations subsequently assigned based on the correlation with clinical outcome („Subgroup identity was initially derived from unsupervised consensus clustering of DNA methylation profiles; risk-based designations were subsequently assigned based on the correlation of each subgroup with clinical outcome data available for our cohort.“ ll. 135ff.)

2) It will be good to use the expression data to confirm that CNV events enriched in HR tumors are accompanied by expected expression change, e.g. amplification of MYC, CDK4, MDM2, and AURKC and deletion of BRCA2, CHEK2 and TP73.

Authors: We thank the reviewer for this suggestion. We have performed the requested analysis and examined whether copy number alterations enriched in HR tumors were accompanied by concordant expression changes for the genes highlighted. The results of this integrative analysis are shown in Supplementary Figure S8A. While a subset of patients showed the expected directional relationship between copy number status and gene expression — with gains at *MYC*, *CDK4*, *MDM2*, and *AURKC* associated with elevated transcript levels and deletions at *BRCA2*, *CHEK2*, and *TP73* associated with reduced expression — the overall

correlation was not consistent across the HR cohort. Expression levels of these genes showed highly heterogeneous patterns across HR patients, with considerable inter-patient variability that could not be explained by copy number status alone. We have added a statement to the manuscript acknowledging this observation and noting that additional regulatory mechanisms beyond copy number — such as the CpG island hypermethylation that characterizes HR tumors, post-transcriptional regulation, or tumor cell compositional effects — may govern the expression of these loci in this subgroup (ll. 252ff.). We believe this finding, while not confirmatory in the classical sense, is itself biologically informative and consistent with the complex epigenetic landscape of HR pACTs described throughout the manuscript.

3) Integrative analysis of methylation status from bulk samples with heterogenous cellular compositions defined by snRNA-seq should be considered. It was striking to see that the HR samples have much higher CpG methylation state compared with the low-risk ones (a median CpG methylation level of 0.62 compared to 0.2), which suggests suppression of gene expression in high-risk samples. On the other hand, the transcriptional diversity of HR samples suggests more active transcriptional program. The authors need to perform an integrative analysis using samples that have both methylation data and snRNA-seq to show whether the methylation status is correlated with tumor snRNA-seq clusters or with the tumor microenvironment.

Authors: To address the potential confounding effect of heterogeneous cellular composition on bulk DNA methylation profiles, we performed an integrative analysis using the subset of tumors for which both bulk methylation data and snRNA-seq profiles were available. Specifically, we tested whether global CpG methylation levels were associated with (i) overall tumor cell abundance inferred from snRNA-seq, (ii) the proportional representation of individual tumor transcriptional states or tumor microenvironmental cell populations, and (iii) tumor pseudobulk transcriptional activity.

Across matched samples, global methylation levels were not correlated with tumor cell fraction (Spearman $\rho = 0.06$, $p = 0.82$), nor with tumor pseudobulk transcriptional activity ($\rho = 0.02$, $p = 0.95$). Furthermore, systematic correlation analyses across all snRNA-seq-defined cell populations revealed no significant association between cluster abundance and bulk methylation levels after multiple testing correction. These findings indicate that the elevated methylation observed in high-risk tumors is unlikely to be explained by differences in tumor purity or enrichment of specific tumor or microenvironmental populations.

We have incorporated these new analyses into the revised Results section and added a corresponding supplementary figure (Supplementary Figure S11), which

illustrates the lack of association between global methylation and tumor cellular composition or transcriptional activity. Together, these data support the interpretation that subgroup-specific methylation differences reflect tumor-intrinsic epigenetic states rather than compositional artifacts arising from bulk sampling.

4) Figure 4A is difficult to interpret. It is stated as a heatmap of pairwise correlations among seven transcriptional metasignatures. However, the number of samples exceeds the 18 used for snRNA profiling and it is unclear why the metasignature counts differ across the samples in second row. More explanation is required. The color scale of Figure 4B is too close to distinguish the different signatures especially those described in the main text (e.g. Cell Cycle Activation).

Authors: We would like to clarify the nature of the analysis shown in Figure 4A, which we acknowledge was not sufficiently explained in the original figure legend. The heatmap does not display pairwise correlations among patients, but rather among all individual NMF programs generated across patients. Since NMF was performed independently for each patient's tumor cell compartment at rank k=4, four programs were generated per patient, resulting in a total of 72 individual programs represented in the heatmap, which therefore considerably exceeds the number of patients profiled by snRNA-seq. Pearson correlation was subsequently used to cluster these individual programs into consensus metaprograms, which were annotated using e.g. enrichR. The varying program counts - visible per sample- reflect how many of each patient's four individual programs were assigned to each metaprogram cluster. We have expanded the figure legend and the Supplementary Methods accordingly to make this workflow explicit and avoid further confusion (Supplementary Methods ll. 290ff.)

Regarding Figure 4B, we agree that the original color scale made it difficult to distinguish between signatures, particularly for the Cell Cycle Activation metaprogram highlighted in the main text. The color palette has been updated in the revised figure to ensure all metaprograms are perceptually distinct and clearly interpretable.

5) The description of the methylation classifier is very brief and more detailed information (i.e. software packages used, version, parameter), training and validation data set used, should be included. This issue applies to all data analysis methods which should be expanded to ensure reproducibility of the results.

Authors: We apologize for the insufficient level of detail in the originally submitted Methods section. A comprehensive Supplementary Methods file has now been

included in the revised manuscript.. For the methylation-based classifier specifically, the Supplementary Methods now provide full details on all software packages used including version numbers, all relevant parameters, and a clear description of the training and validation datasets employed. In line with the reviewer's broader concern, all other data analysis methods have likewise been expanded throughout the Supplementary Methods to ensure transparency and reproducibility of the results.

Minor comments

1) Supplementary Figure 1E: it will be good to provide the description of clinical features so that readers who are not familiar with clinical features of adrenocortical tumor can understand this graph. The narrative accompanying this figure panel at lines 168-174 also lacks the description of these clinical features. Similarly, the importance of Ki-67 proliferation index and MKI67 transcript levels and the data source needs to be introduced.

Authors: We thank the reviewer for these constructive suggestions. We have added a brief introductory passage at the beginning of this paragraph providing accessible descriptions of the key clinical presentations in pACT (virilization, Cushing syndrome, and hypertension-related symptoms) to ensure the figure and accompanying narrative are interpretable for readers outside the clinical field. („Pediatric adrenocortical tumors frequently present with signs of hormonal excess reflecting the steroidogenic activity of the tumor. Virilization, resulting from androgen overproduction, is the most common presentation and manifests as premature pubic hair, clitoral or penile enlargement, and accelerated growth. Cushing syndrome arises from excess cortisol secretion and is characterized by weight gain, facial rounding, hypertension, and growth retardation, while hypertension-related presentations are associated with mineralocorticoid or mixed steroid excess.“ ll. 185ff.) Furthermore, we have added a sentence introducing the Ki-67 proliferation index and clarifying that Ki-67 protein levels were assessed by immunohistochemistry on tumor tissue sections, while MKI67 transcript levels were derived from bulk RNA panel data, prior to reporting the results of these analyses. („The Ki-67 proliferation index is a widely used marker of tumor cell proliferation, assessed here by immunohistochemistry on tumor tissue sections, while MKI67 transcript levels were derived from bulk RNA sequencing data.“, ll. 203 ff.)

2) Line 141-144: Figure S2 shows that risk stratification based on methylation grouping was significant in pACC but not in the other two diagnostic groups (i.e. UMP and ACA). The current writing, "The added value of the subgroups, considering the prognostic positive

pACA group only, is limited (Supplementary Figure S2).”, reads quite awkward and needs to be clarified.

Authors: We thank the reviewer for this observation. We agree that the original phrasing was unclear. The sentence has been revised to explicitly state that methylation-based subgrouping carries prognostic significance in pACC but not in the UMP and ACA diagnostic groups, which more accurately reflects the data shown in Supplementary Figure S2. („Methylation-based subgrouping showed prognostic significance in pACC, whereas no significant association with outcome was observed in the UMP and ACA diagnostic groups (Supplementary Figure S2).“ ll. 152 ff.)

3) The reclassification of 15 Clay A1 tumors only showed 7 were assigned to the high-risk group. The authors mentioned that 7 out of 8 reclassified A1 tumors survived the disease (line 158-160). Can the authors demonstrate that the risk stratification is improved over the original Clay grouping by presenting a survival curve for the Clay cohort using these two classification schemes?

Authors: We thank the reviewer for this constructive suggestion. To directly address whether our methylation-based risk stratification improves upon the original Clay et al. classification, we performed survival analyses in the Clay cohort using both classification schemes and present the resulting survival curves below.
Using the original Clay classification, A1 tumors showed significantly inferior overall survival compared to A2 tumors (log-rank $p = 7\times10^{-5}$), confirming the prognostic relevance of the original grouping. However, when the same cohort was reclassified using our methylation-based subgroups, the separation between High Risk and Low Risk patients was substantially more pronounced (log-rank $p = 5\times10^{-7}$), demonstrating improved prognostic resolution. Notably, the 7 Clay A1 tumors reclassified as High Risk by our classifier accounted for 5 of the 6 observed deaths in the A1 group, whereas the single death among Low Risk-reclassified samples occurred in a Low Risk 3 patient. These findings demonstrate that our methylation-based classification refines the prognostic stratification provided by the original Clay grouping, particularly by identifying within the heterogeneous A1 group those patients at genuinely high risk of disease-related death.

[REDACTED]

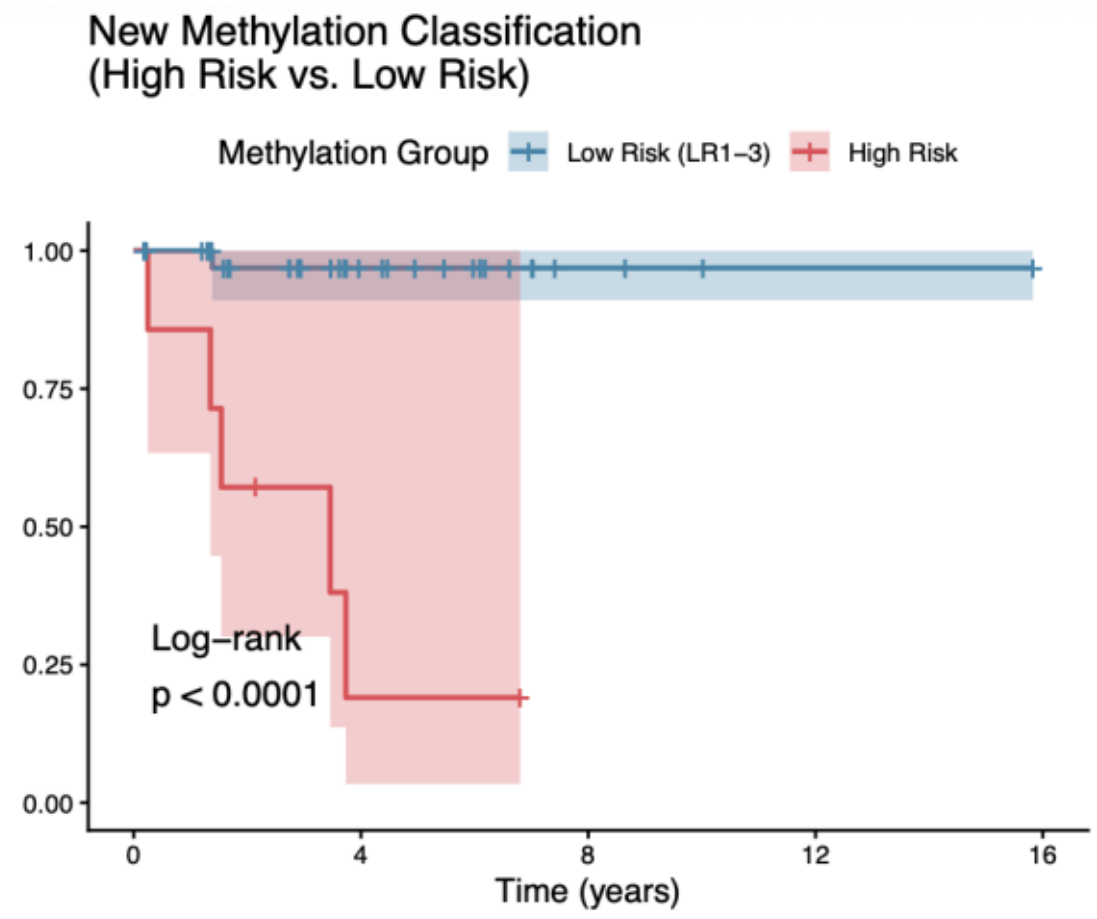

4) Figure 2F shows pie-charts for CTNNB1 mutation frequency in each subgroup. It will be better to use bar plots (one bar for each subgroup) for showing the frequency of all key driver genes in these four subgroups with the significance for enrichment in a particular group labeled.

> Authors: We thank the reviewer for this suggestion. Figure 2F has been revised to display mutation frequencies as bar plots rather than pie charts, with one bar per subgroup. Statistical significance for enrichment of CTNNB1 mutation in a particular subgroup is now labeled directly on the figure. Bar plots for all remaining key driver genes with associated statistics have been included in Supplementary Figure S9 to provide a comprehensive overview of the mutational landscape across subgroups without overloading the main figure.

5) Figure 3B which introduces the samples used for snRNA-seq should be placed as the first panel (Fig. 3A) instead. Figure 3C legend: clusters are labeled as zR zF instead of zF zR as in the other parts of the manuscript. Cluster labels in Figure 3C and 3F are inconsistent which should be corrected.

> Authors: We have addressed all points in the revised manuscript. The panel introducing the samples used for snRNA-seq (previously Figure 3B) has been repositioned as the first panel, now Figure 3A, to improve the logical flow of the figure. Furthermore, the cluster label order in the Figure 3C legend has been corrected from "zR zF" to "zF zR" to ensure consistency with the nomenclature used throughout the manuscript, and the cluster labels in Figures 3C and 3F have been harmonized accordingly.

6) The finding of “Regulatory Motif Analysis Suggests CTNNB1- and AP1-binding motifs as Drivers of Tumor Biology in High Risk pACTs” was based on the scATAC-seq data generated from the reproducibility analysis of the preceding section and it would be good to provide a background when introducing this analysis.

Authors: We thank the reviewer for this suggestion. We have added a brief introductory sentence at the beginning of this section to clarify that the regulatory motif analysis is based on the snATAC-seq data generated during the reproducibility analysis presented in the preceding section, thereby providing the reader with the appropriate context before the analysis is introduced (ll. 444 ff.).

7) Line 101: "his work" should be "this work"?
8) Line 391: "dedifferentiated zG-like cells" should be "undifferentiated zG-like cells"?

Authors: We thank the reviewer for these careful corrections. The typo "his work" at line 101 has been corrected to "this work", and the term "dedifferentiated" has been replaced with "undifferentiated" throughout the manuscript.

Reviewer #3, expertise in single cell and spatial omics for adrenocortical carcinoma (Remarks to the Author):

The manuscript by Fincke et al. presents a multi-omic analysis—including methylation, bulk transcriptome, exome, single-cell RNA-seq, ATAC-seq, and spatial transcriptomics—of pediatric adrenocortical tumors. This original and unique dataset for these rare pathologies identifies, through methylome profiling, a subgroup of tumors associated with poor prognosis (the high-risk, HR, group), characterized by CpG island hypermethylation and concomitant activation of the Wnt and AP-1 pathways. In addition, the authors show in a preclinical study that this tumor subgroup may be sensitive to HDAC inhibitors, opening new therapeutic perspectives for these rare pediatric tumors.
Overall, the datasets generated in this study represent an important resource for the scientific community and provide new insights into the biology of pediatric adrenocortical tumors that could ultimately improve patient management. However, several points require clarification or further detail, mainly regarding the analytical methods and, to a lesser extent, some of the results.

Authors: We thank Reviewer 3 for the thorough and generous evaluation of our manuscript and for recognizing the novelty and importance of this multi-omic dataset for the pediatric adrenocortical tumor field. We are pleased that the identification of the high-risk subgroup, its epigenetic and transcriptional hallmarks, and the preclinical evidence for HDAC inhibitor sensitivity are considered meaningful contributions with potential clinical impact. We have carefully addressed all points requiring clarification, as detailed point by point below.

Major points

1- The Methods section is insufficiently detailed and does not allow reproduction of the analyses or proper assessment of their scientific validity. Although the authors refer to Supplementary Materials containing detailed methods, this file was not provided.
For instance, the authors developed a random forest model to classify new patients based on methylation data, but it is unclear how batch effects from independent methylation series would be handled, how the nested cross-validation was performed (number of folds, stratification by risk group), or how classifier performance was assessed (e.g., average performance across outer folds).
Similarly, the exome analysis is described only briefly: "Tumor-normal paired WES was performed at 50× coverage using Agilent SureSelect V8 capture. Variants were called using six algorithms, annotated with VEP, and filtered against population databases." The alignment algorithm and reference genome are not specified, the six variant-calling tools are not listed, and the annotation process is not described. VEP should be defined, and details should be provided regarding population frequency thresholds, databases used for filtering, and any applied quality, allelic fraction, or functional consequence filters.
This lack of methodological transparency is particularly problematic given that the analysis code is not made available to the community.
Similar concerns apply to the entire Methods section.

> Authors: We thank the reviewer for this important point. This file has now been included in the revised manuscript and addresses the specific concerns raised in detail.
> Regarding the methylation-based random forest classifier, the Supplementary Methods now specify: how batch effects between independent methylation series were handled prior to classification; the nested cross-validation scheme, including the number of inner and outer folds and stratification by risk group; and the metrics used to assess classifier performance, reported as averages across outer folds.
> Regarding the whole-exome sequencing analysis, the Supplementary Methods now provide: the alignment algorithm and reference genome used; the names and versions of all six variant-calling tools employed; and full details of the filtering pipeline, including population frequency thresholds, databases used, and applied quality, allelic fraction, and functional consequence filters.
> Regarding code availability, the analyses were performed using established bioinformatics tools and pipelines as now described in full methodological detail in the Supplementary Methods.

In addition, the sample size (N) for each analysis, including bulk transcriptome and exome analyses, should be clearly stated.

> Authors: We thank the reviewer for this suggestion. The sample size for each analysis has now been explicitly stated throughout the manuscript, including in the relevant methods and results sections and figure legends. This includes the number

of samples included in the DNA methylation profiling, bulk transcriptome analysis, whole-exome sequencing, snRNA-seq, snATAC-seq, and spatial transcriptomics analyses respectively.

2- Regarding the exome analysis, a table summarizing the identified variants is missing. At minimum, somatic variants should be reported, especially if germline variants cannot be described for regulatory reasons.
In particular, the reported 10% frequency of somatic ARMC5 variants is surprising, as this gene is classically described as constitutively altered in bilateral macronodular adrenal disease.
Moreover, providing tumor mutational burden (TMB) values across the different molecular or risk groups would add useful contextual information.

Authors: We thank the reviewer for these important points and have addressed them as follows.
Regarding the variant table, a comprehensive summary of all identified somatic variants including variant classification, functional consequence, population allele frequencies, and sequencing metrics is now provided as Supplementary Table S9. We note that for a subset of samples, tumor-normal paired sequencing with germline subtraction was available, while the remaining samples were analyzed from tumor DNA only with population frequency filtering applied to enrich for likely somatic variants. This distinction is clearly annotated in the table.
Regarding ARMC5, we thank the reviewer for this observation. The apparent 10% frequency should indeed be interpreted with caution. ARMC5 is canonically associated with primary bilateral macronodular adrenocortical hyperplasia, and its occurrence in our predominantly unilateral pediatric cohort is unexpected. Given that a substantial proportion of our samples lack matched germline controls, we cannot exclude that some of these variants represent germline alterations rather than somatic events, which would be entirely consistent with the known germline nature of ARMC5 mutations in bilateral disease. We have added a cautionary note to the manuscript acknowledging this limitation.

Regarding TMB, we have decided not to report TMB values in the manuscript due to the fundamental methodological heterogeneity across our cohort. As illustrated in the supplementary figure provided below, samples without germline subtraction show dramatically inflated apparent mutation burden compared to matched tumor-normal pairs, reflecting the inclusion of germline variants rather than true somatic mutational load. Reporting TMB across these two analytically distinct groups would be misleading, and we therefore consider it more appropriate to omit this metric pending future analyses with uniformly matched sequencing data.

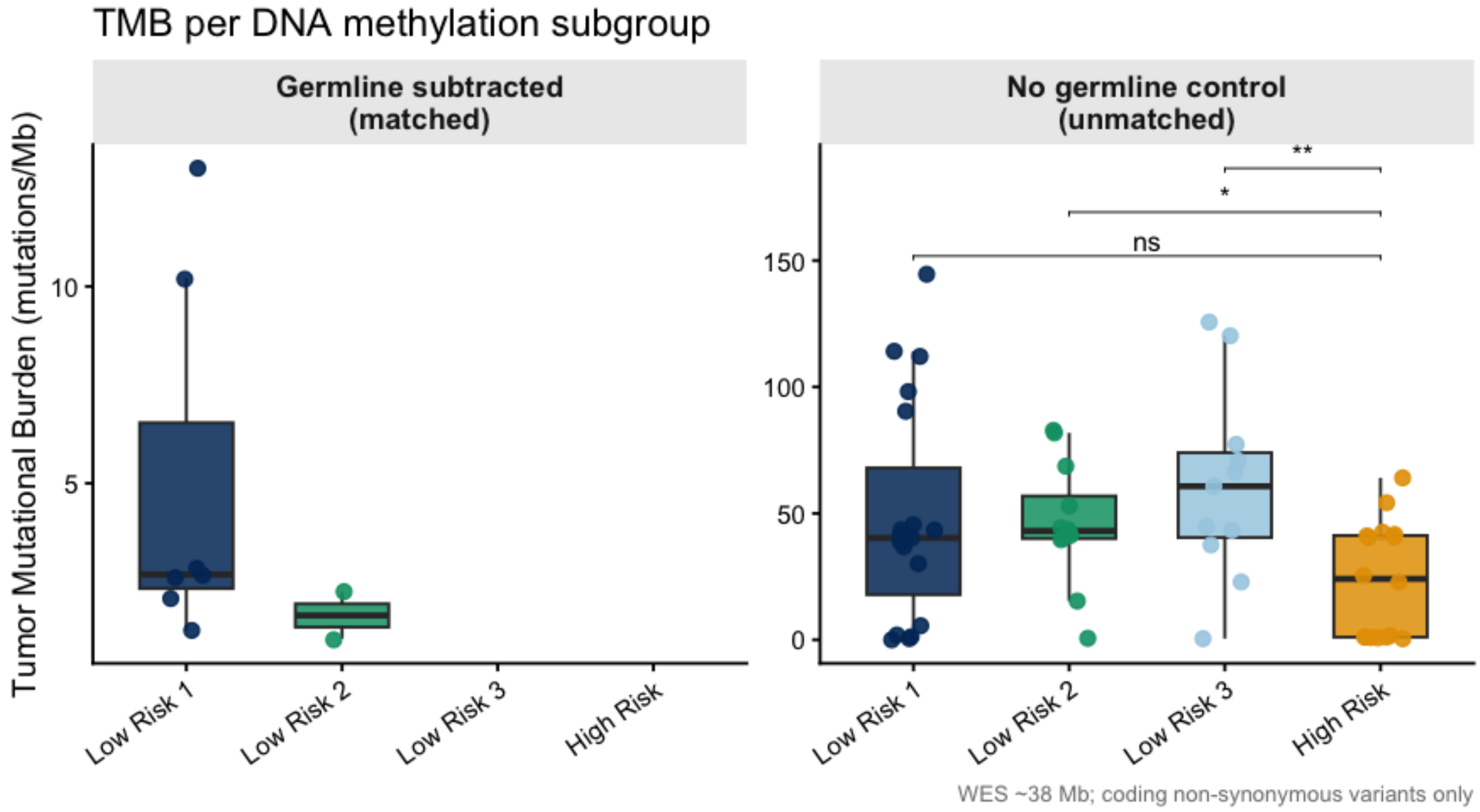

3- For the single-cell RNA-seq and spatial transcriptomics analyses, the number of ACC, UMP, and ACA tumors included should be specified to facilitate interpretation. It would also improve clarity to label tumors according to their histological type (ACC, ACA, or UMP) rather than using the generic term ACT in Figures 3-4.

Authors: We thank the reviewer for this suggestion. The number of ACC, UMP, and ACA tumors included in the analyses has been specified in the relevant figure legends of Figures 3 and 4.

The single-cell analysis provides a clear and interesting description of steroidogenic cell heterogeneity, and these findings partly overlap with those reported in adult single-cell atlases (PMID: 38759836, 39167619, 41053133). Discussing similarities and differences with adult tumors would strengthen the manuscript.
In addition, differences in the tumor microenvironment are likely, as recently reported in adult adrenocortical tumors (PMID: 41053133). A dedicated analysis of immune and stromal compartments could help characterize cell types and cellular states and evaluate how they associate with the defined risk groups.

Authors: We thank the reviewer for these important and closely related suggestions, which we have addressed together. Regarding the comparison with adult single-cell atlases, we have expanded the Discussion to address similarities and differences with the three referenced studies. The macrophage and lymphoid depletion we observe in HR pACTs is consistent with findings by Jouinot et al. and Tourigny et al. in adult ACC, and the zG-like dedifferentiated population enriched in HR tumors resonates with ZG-like clusters identified

by Altieri et al. and Tourigny et al. in adult adrenocortical tumors with active WNT signaling. We also highlight key differences, including the absence of an equivalent methylation-based risk stratification in adult ACC and the novel co-activation of AP-1 alongside WNT as a defining feature of HR pACTs, underscoring the biological distinctiveness of aggressive pediatric adrenocortical tumors.
Regarding the TME analysis, we have performed a systematic quantification of immune and stromal cell type proportions across risk groups using our snRNA-seq data. This revealed significant differences in macrophage and lymphoid cell abundance (Kruskal-Wallis, $p = 0.047$ and $p = 0.049$, respectively), with HR tumors showing marked depletion of both populations compared to LR subgroups. These findings have been incorporated into the Results (ll. 288 ff.) and Discussion sections (ll. 675 ff.).

For the spatial analyses, the dispersion of cell populations could be quantitatively assessed using spatial autocorrelation metrics, such as Moran's I, rather than being described solely by visual inspection.

> Authors: We thank the reviewer for this helpful suggestion. In response, we performed a quantitative assessment of spatial cell population dispersion using Moran's I spatial autocorrelation metrics for each annotated cell type across tumors. Consistent with the qualitative observations, most populations exhibited low spatial autocorrelation (median Moran's I ≈ 0.01, typically ranging from 0.00–0.03). However, the high-risk tumor showed increased spatial clustering in selected populations, most prominently fibroblasts (Moran's I ≈ 0.17), as well as a higher overall mean Moran's I compared to LR tumors (0.053 vs. 0.011–0.021 across LR samples). These results support the presence of localized cellular niches within the HR tumor despite globally reduced compartmentalization. The corresponding description has now been incorporated into the Results section (ll. 506 ff.).

4- The results presented in this study could potentially be leveraged to propose a clinically useful decision tree for patient stratification, for example by sequentially considering histological subtype (ACC versus UMP), age (> 5 years?), CTNNB1 immunohistochemistry status, and methylation-based risk group.

> Authors: We thank the reviewer for this valuable suggestion. We agree that translating our findings into a clinically actionable framework represents an important contribution of this study. We have therefore developed a proposed decision tree for risk stratification of pACT patients at diagnosis, which is now included as a new Supplementary Figure 22. In this decision tree, histological

subtype serves as the first decision node, with ACA classified as benign requiring no further molecular workup. For ACC and UMP, DNA methylation-based classification using the EPIC array is proposed as the primary stratification tool, directly assigning patients to LR1–3 or HR subgroups with associated treatment implications. In settings where methylation profiling is not immediately available, interim risk assessment using CTNNB1 IHC and mutation status in combination with age serves as a surrogate, with methylation profiling recommended as soon as feasible to confirm the classification. We deliberately placed methylation profiling as the primary decision node rather than following the sequential order suggested by the reviewer, as our data demonstrate it is the most definitive and comprehensive classifier. CTNNB1 and age are retained as a practical fallback for settings with limited infrastructure. A brief note explicitly stating that this framework requires prospective validation in independent cohorts before clinical implementation has been included in the manuscript.

Minor points / Typos

- Lines 132–135: reporting survival values consistently as percentages rather than proportions would improve clarity, as “HR: 0.25” may be misinterpreted as a hazard ratio rather than a 5-year survival rate.
- Line 191: “Fig. S5” should be cited instead of “Fig. 5”.
- Lines 258–259: it would be helpful to specify whether more ZR-specific steroidogenic genes, such as CYB5A, CYB5B, or SULT2A1, were examined.
- Line 331: “Figure 4E” should be cited instead of “Figure 5E”.
- Figure 4: risk groups should be indicated as clearly as possible in all panels.
- Supplementary Figure S11C: labels are misaligned and overlap the panel.

This is now figure S12C, the overlapping has been corrected.

- Supplementary Figure S14: histological characterization is missing (Supplementary Figure S16 appears to be provided twice).
- Supplementary Figure S15: the meaning of the red vertical dashed line should be clarified.
- Supplementary Table 1: the title of column D is missing, and the title of column W is unclear.

Authors: We thank the reviewer for this suggestion, all of the aforementioned points have been corrected

Reviewer #1 (Remarks to the Author)

Overall, the authors responded well to reviewer comments. Still there are a few remaining issues:

1. Supplementary Figure S15 shows no major differences in AP-1 binding motifs in HR compared to LR1. This should be explicitly mentioned in the text even though I understand that authors emphasize that HR has both WNT and AP-1 activity compared to LR1 which has only AP-1 activity.

Authors: We thank the reviewer for this observation. We have revised the text to explicitly acknowledge that no major difference in AP-1 binding motif accessibility is observed between HR and LR1, and to clarify that the key distinction lies in the co-activation of WNT signaling alongside AP-1 programs in HR tumors, while LR1 activity appears restricted to AP-1 alone (ll. 433ff.).

2. It's good that the authors added Supplementary Figure S17 but it's uninterpretable to the reader. There needs to be some summary statistics of each spatial panel.

Authors: We thank the reviewer for this feedback. We have revised Supplementary Figure S17 to include summary statistics for each spatial panel, adding both the number of positive cells per region and a boxplot comparing HR versus LR tumors. We believe the figure is now more interpretable and supports the conclusions drawn in the main text.

3. The AP-1 qRT-PCR data is a good addition but the legend descriptions for Figure 6 are completely off and do not match the data. Authors need to fix this.

Authors: The figure legends for Figure 6 have been corrected and now accurately describe the AP-1 qRT-PCR data presented.

4. Access to genomic datasets: the GHCA link does not work, this needs to be corrected

Authors: We thank the reviewer for flagging this. The GHCA link has been corrected in the revised manuscript.

5. DNA methylation and single cell RNA-seq data from H295R and CU-ACC1 cells with and without drug treatments needs to be uploaded to GEO

Authors: DNA methylation and single-cell RNA-seq data from H295R and CU-ACC1 cells with and without drug treatment have been uploaded to GEO (accession number GSE336546 for scRNAseq & GSE336709 for methylation).
