# Supplementary material for: CpG hypermethylation and WNT/AP-1 cooperativity define the epigenetic landscape and a clinical subgroup of high-risk pediatric adrenocortical carcinoma
Source: Nat Commun. 2026 Sep 14;17:9786. doi: 10.1038/s41467-026-77225-5 (PMC13575229; doi:10.1038/s41467-026-77225-5)
Supplement: Supplementary file 3 [file 41467_2026_77225_MOESM1_ESM.pdf]

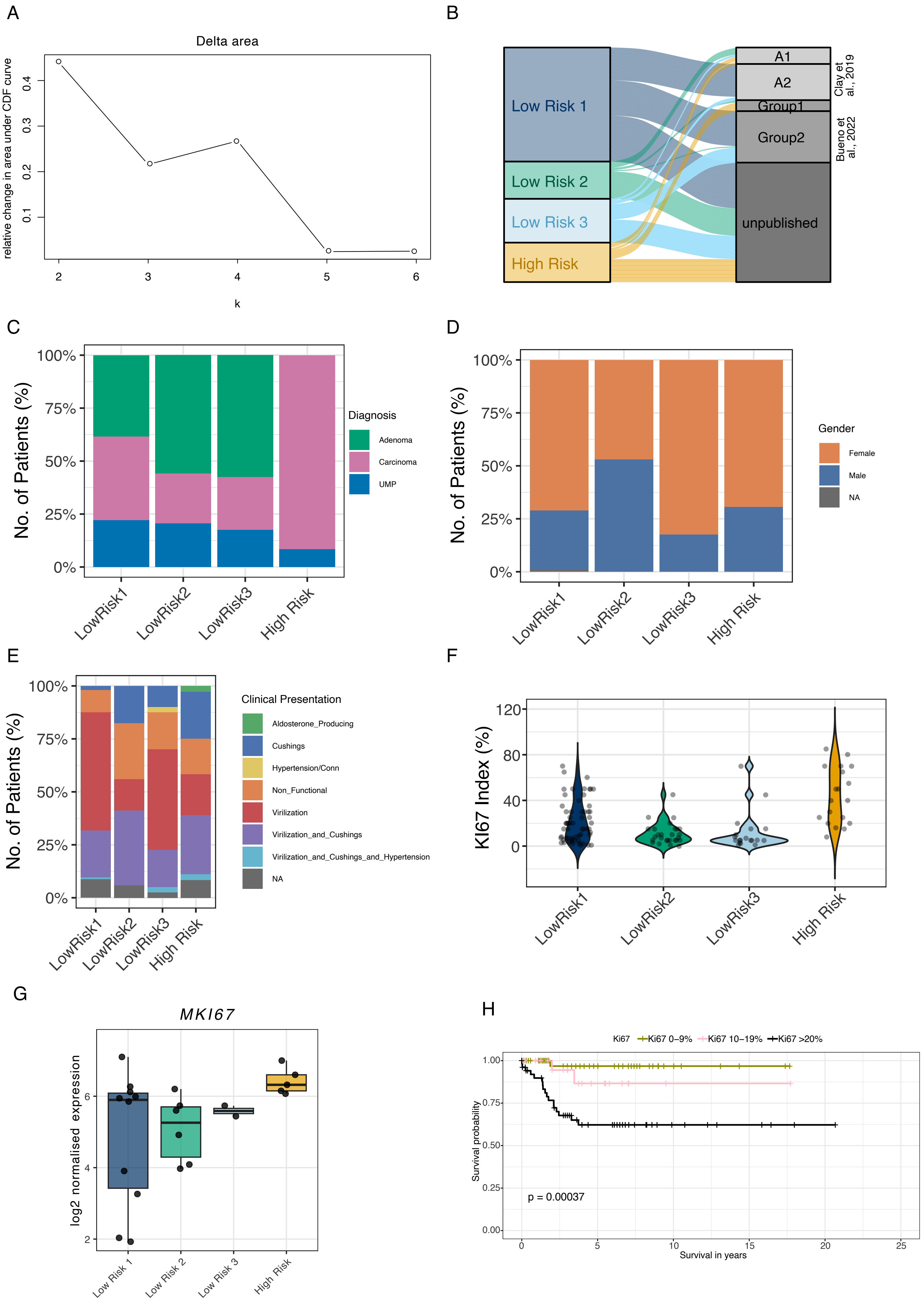
A
Delta area
relative change in area under CDF curve
k
B
Low Risk 1
Low Risk 2
Low Risk 3
High Risk
A1
A2
Group1
Group2
unpublished
Clay et al., 2019
Bueno et al., 2022
C
No. of Patients (%)
Diagnosis
Adenoma
Carcinoma
UMP
LowRisk1
LowRisk2
LowRisk3
High Risk
D
No. of Patients (%)
Gender
Female
Male
NA
E
No. of Patients (%)
Clinical Presentation
Aldosterone_Producing
Cushings
Hypertension/Conn
Non_Functional
Virilization
Virilization_and_Cushings
Virilization_and_Cushings_and_Hypertension
NA
F
KI67 Index (%)
G
MKI67
log2 normalised expression
Low Risk 1
Low Risk 2
Low Risk 3
High Risk
H
Ki67
Ki67 0–9%
Ki67 10–19%
Ki67 >20%
Survival probability
p = 0.00037
Survival in years

Supplementary Figure S1. Clinical and Histological Features Across DNA Methylation–Based pACT Subgroups.

(A) Relative change in the area under the cumulative distribution function (CDF) curve used to determine the optimal number of clusters during consensus clustering of DNA methylation profiles.
(B) Comparison of previously reported subgroup classifications (Clay et al., 2019; Bueno et al., 2022) with newly defined methylation-based subgroups, showing partial overlap in subgroup assignments (total n=214; HR, n = 36; LR1, n = 104; LR2, n = 34; LR3, n = 40).
(C) Histopathological diagnosis across subgroups (Adenoma n=81;Carcinoma n=93; UMP n=35).
(D) Gender distribution across subgroups (Female n=14 ; Male n=66).
(E) Clinical presentation across risk groups, including virilization, Cushing's syndrome, hypertension, and non-functional tumors (Aldosterone poduction n=1; Cushings n=20; Hypertension=1; Non functional n=34; Virilization n=94; Virilization and Cushings n=55).
(F) Ki-67 index percentages stratified by subgroup, with no significant enrichment of high Ki-67 in the High Risk group (n=125).
(G) MKI67 expression across the molecular subgroups (n=23). Box plots show the median and interquartile range, whiskers extend to 1.5 × IQR, with individual samples overlaid. Kruskal–Wallis omnibus test; pairwise comparisons by two-sided Wilcoxon rank-sum test with Benjamini–Hochberg correction across the six subgroup pairs (* P~adj~ < 0.05, **P~adj~ < 0.01, *** P~adj~ < 0.001, **** P~adj~ < 0.0001). Exact values are provided in Source Data.
(H) Overall survival of patients stratified by Ki-67 proliferation index (0–9%, n=49; 10–19%, n= 23; >20%, n=53), showing that Ki-67 >20% is associated with poorer prognosis.Groups were compared by two-sided log-rank test. Crosses indicate censored observations. Patients with a Ki-67 index >20% showed shorter overall survival. Source data are provided with this paper.

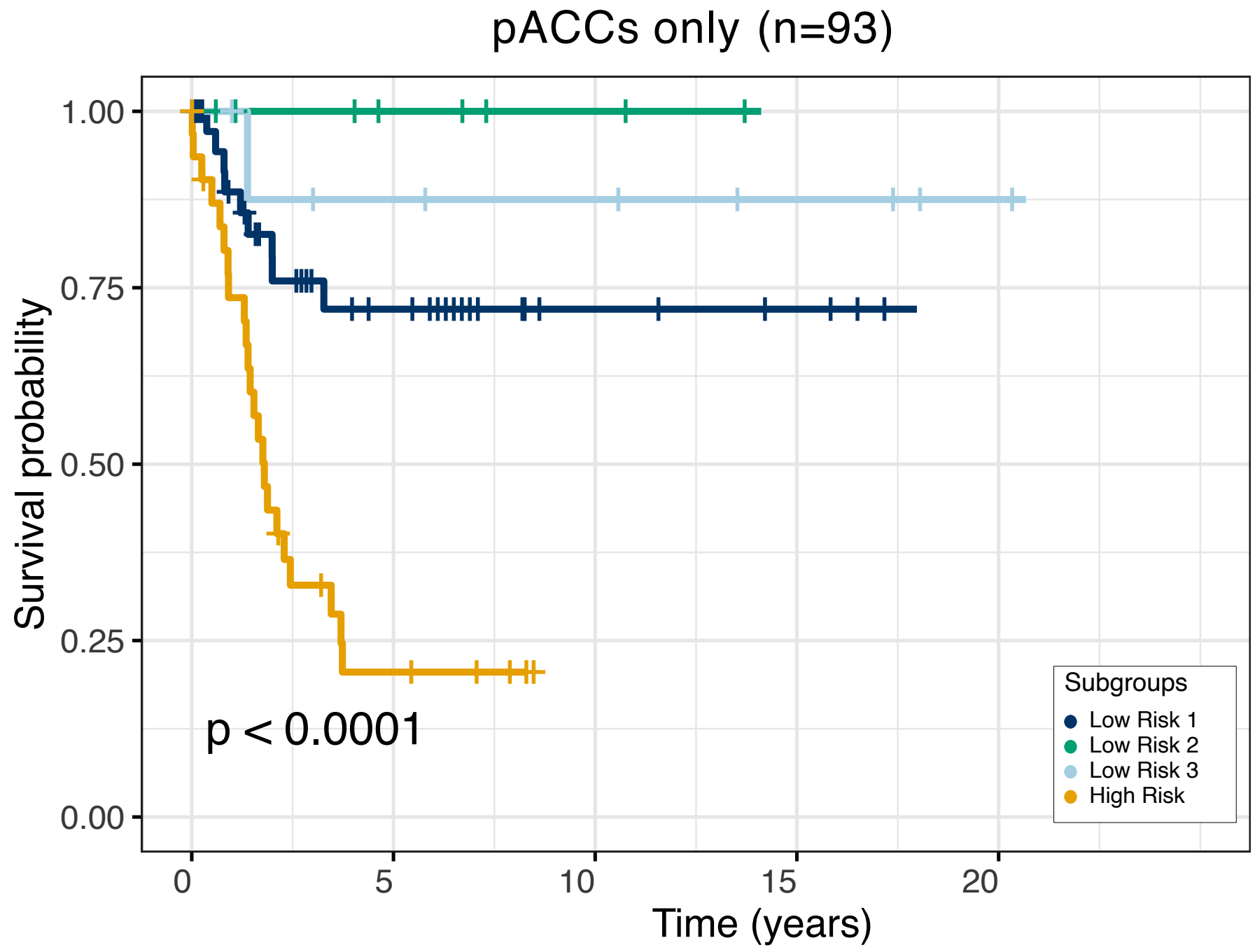
pACCs only (n=93)
Survival probability
1.00
0.75
0.50
0.25
0.00
p < 0.0001
0
5
10
15
20
Time (years)
Subgroups
Low Risk 1
Low Risk 2
Low Risk 3
High Risk

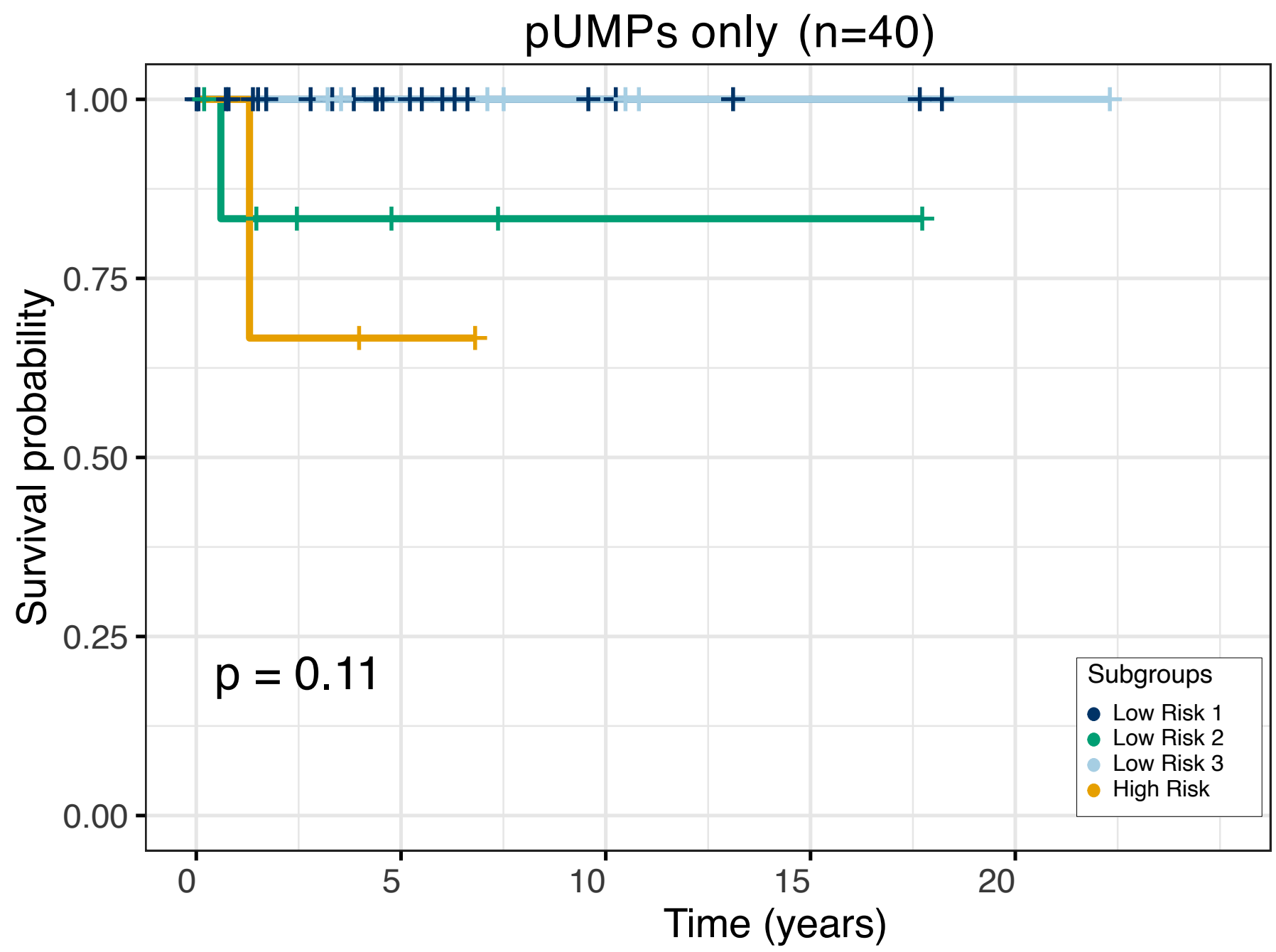
pUMPs only (n=40)
Survival probability
1.00
0.75
0.50
0.25
0.00
p = 0.11
0
5
10
15
20
Time (years)
Subgroups
Low Risk 1
Low Risk 2
Low Risk 3
High Risk

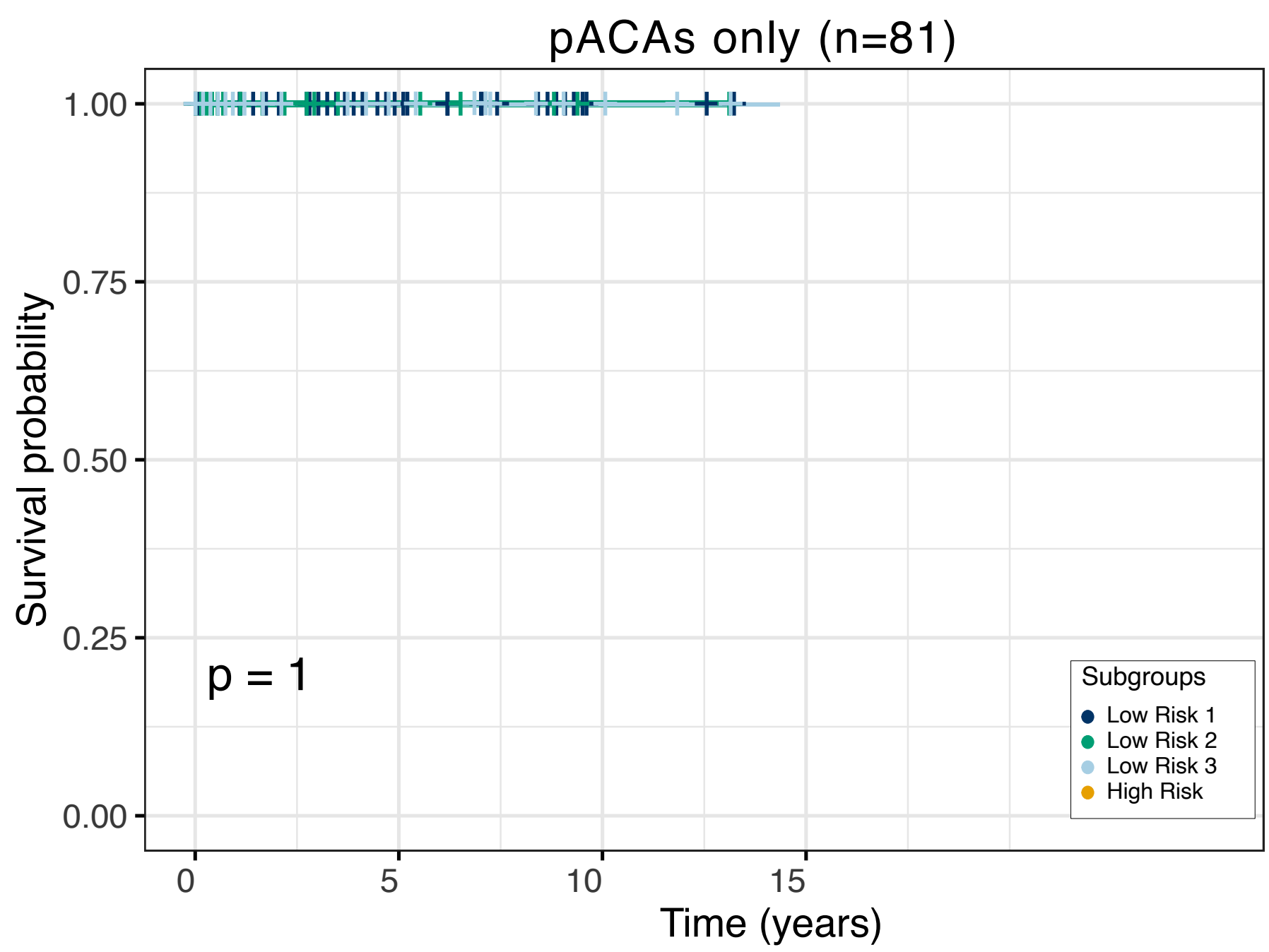
pACAs only (n=81)
Survival probability
1.00
0.75
0.50
0.25
0.00
p = 1
0
5
10
15
Time (years)
Subgroups
Low Risk 1
Low Risk 2
Low Risk 3
High Risk

Supplementary Figure S2. Prognostic Relevance of DNA Methylation-Based Subgroups Across Histopathological Classifications.

Kaplan–Meier survival analyses stratified by DNA methylation-based subgroups within histologically defined pediatric adrenocortical tumor (pACT) categories: (Top) pediatric adrenocortical carcinomas (pACCs, n = 93); (Middle) tumors of uncertain malignant potential (pUMPs, n = 40); and (Bottom) adrenocortical adenomas (pACAs, n = 81). Survival probabilities are plotted over time in years, with log-rank p-values indicating statistical significance. The High Risk subgroup (orange) shows significantly reduced survival in pACCs ($p < 0.0001$), but no survival difference was observed within pUMPs ($p = 0.11$) or pACAs ($p = 1$), underscoring the specific prognostic relevance of methylation-defined subgroups in malignant cases.

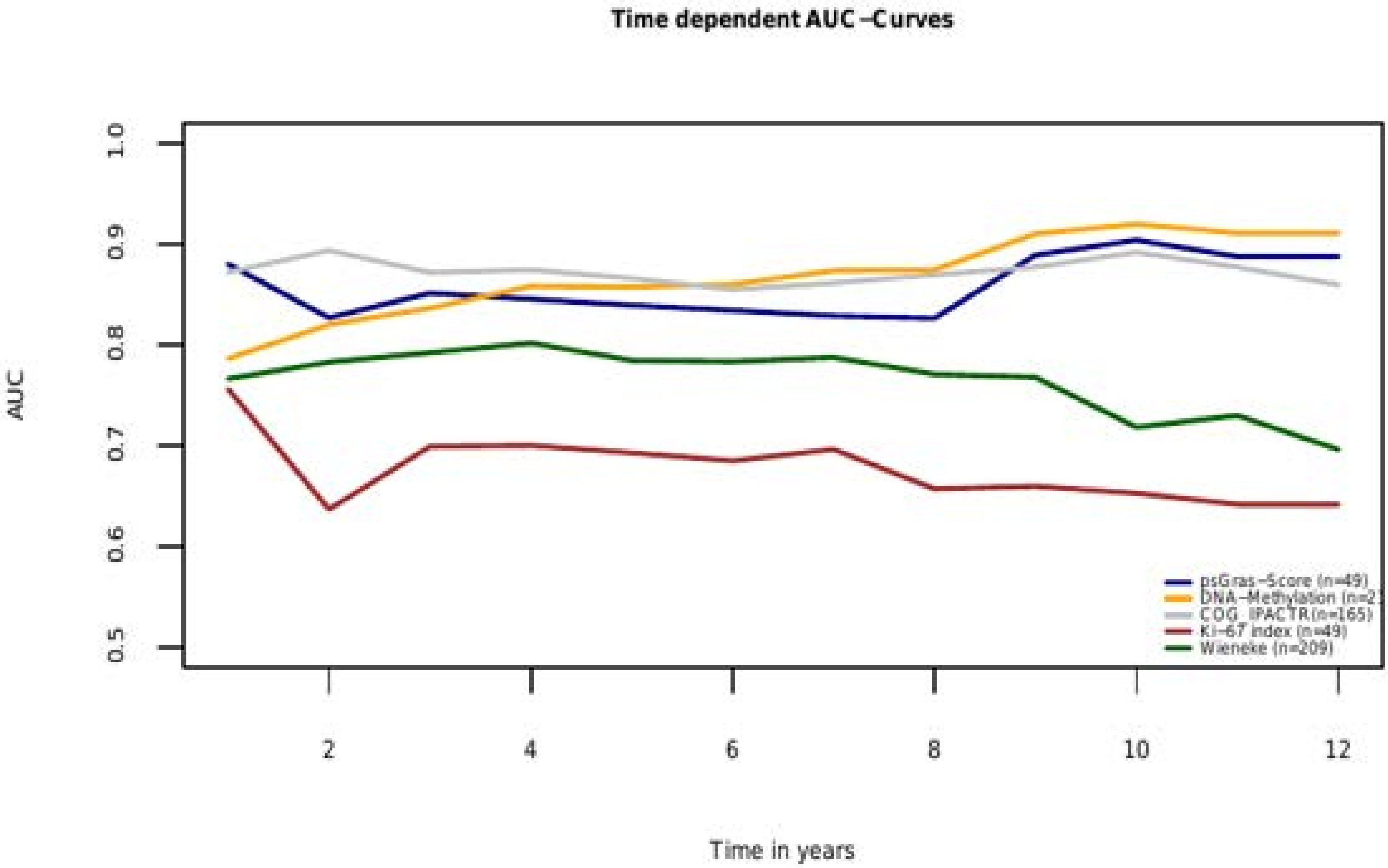
Time dependent AUC –Curves
AUC
1.0
0.9
0.8
0.7
0.6
0.5
2
4
6
8
10
12
Time in years
psGras–Score (n=49)
DNA–Methylation (n=2
COG_IPACTR(n=165)
Ki–67 index (n=49)
Wieneke (n=209)

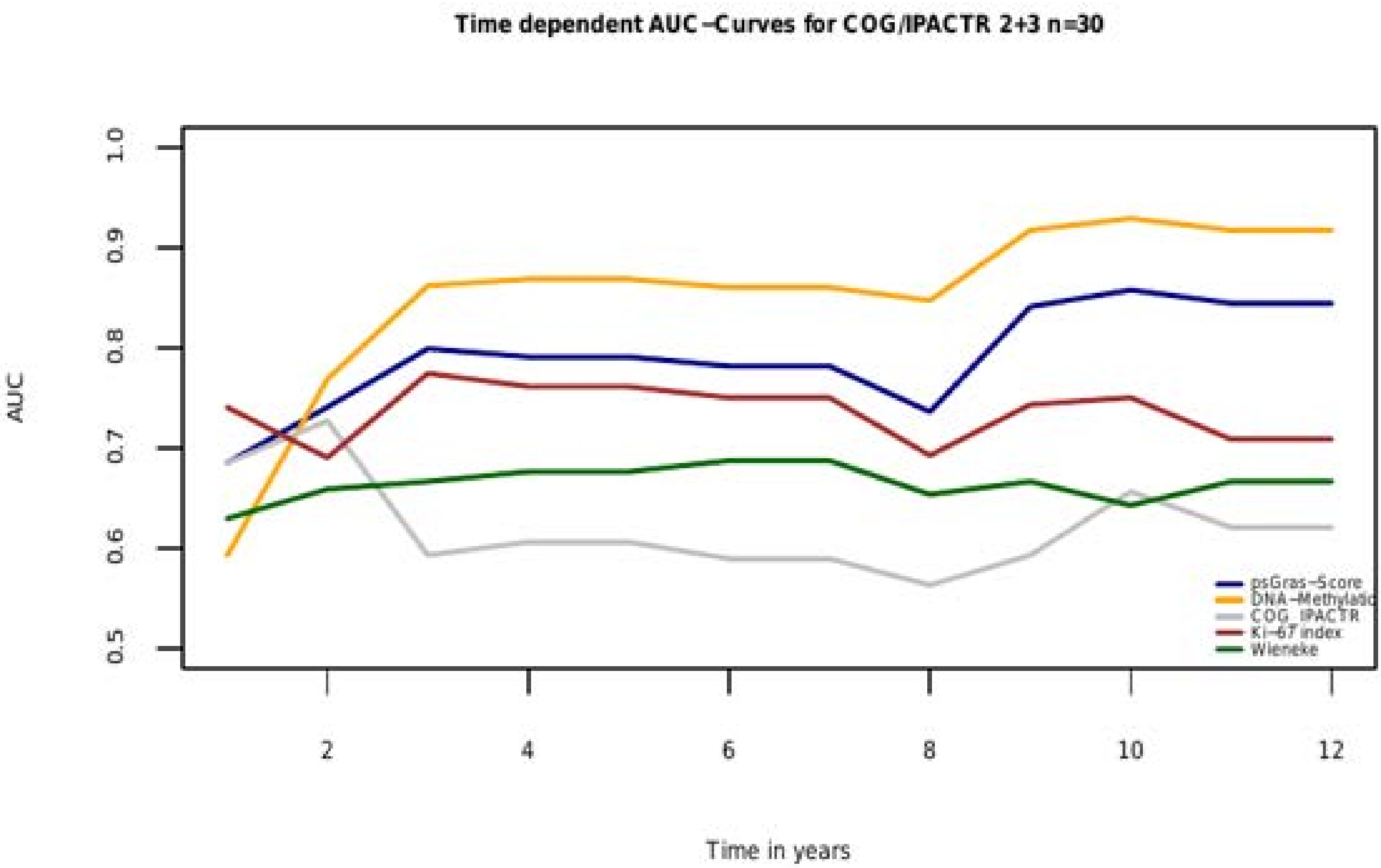
Time dependent AUC –Curves for COG/IPACTR 2+3 n=30
AUC
1.0
0.9
0.8
0.7
0.6
0.5
2
4
6
8
10
12
Time in years
psGras–Score
DNA–Methylatio
COG_IPACTR
Ki–67 index
Wieneke

Supplementary Figure S3. Prognostic performance of DNA methylation-based risk groups compared to clinical classifiers in pediatric ACT. Area under the curve (AUC) is plotted for the whole cohort (top) (psGras-Score n=49; DNA-Methylation n=214; COG-IPAC7R n=165; Ki-67 n=49; Wieneke n=209) and for patients with COG or IPACTR 2 or 3 (n=30) only (bottom).

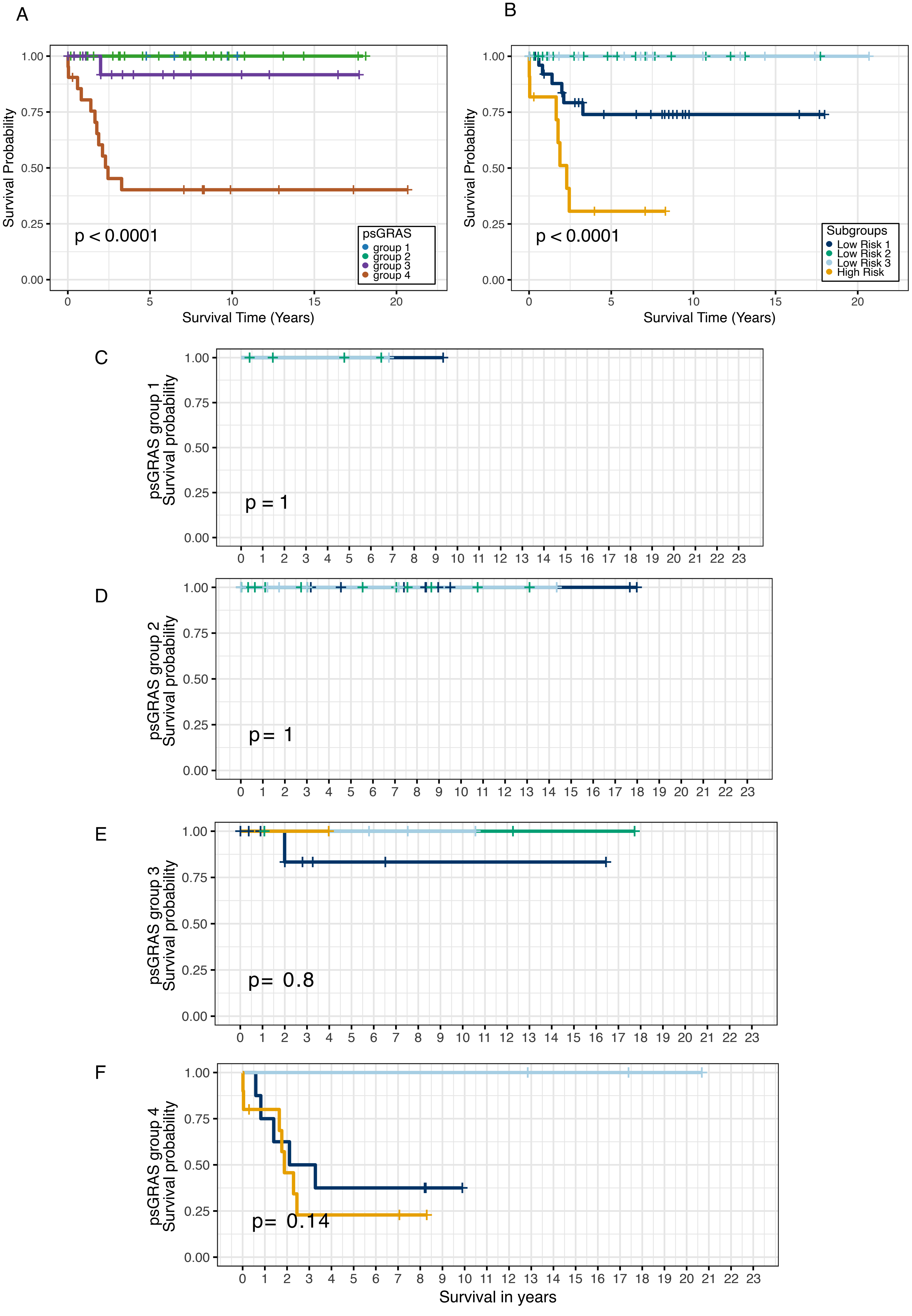
A
Survival Probability
1.00
0.75
0.50
0.25
0.00
p < 0.0001
psGRAS
group 1
group 2
group 3
group 4
0
5
10
15
20
Survival Time (Years)
B
Survival Probability
p < 0.0001
Subgroups
Low Risk 1
Low Risk 2
Low Risk 3
High Risk
Survival Time (Years)
C
psGRAS group 1
Survival probability
p = 1
D
psGRAS group 2
Survival probability
p= 1
E
psGRAS group 3
Survival probability
p= 0.8
F
psGRAS group 4
Survival probability
p= 0.14
0 1 2 3 4 5 6 7 8 9 10 11 12 13 14 15 16 17 18 19 20 21 22 23
Survival in years

Supplementary Figure S4. Comparative Prognostic Value of psGRAS Versus DNA Methylation-Based Subgroups in Pediatric Adrenocortical Tumors.

(A) Kaplan–Meier survival analysis stratified by psGRAS risk groups 1–4 across the full cohort (n = 49), demonstrating significant differences in survival (log-rank $p < 0.0001$). (B) Kaplan–Meier curves for the same cohort stratified by DNA methylation-based subgroups, also showing significant prognostic discrimination (log-rank $p < 0.0001$). (C–F) Survival analyses within individual psGRAS groups further stratified by DNA methylation-based subgroups: group 1 (n=6) (C), group 2 (n=28) (D), group 3 (n=17) (E), and group 4 (n=21) (F). No significant survival differences were observed within individual psGRAS strata based on methylation subgroup classification (p-values indicated), suggesting that methylation-based subgroups offer complementary and potentially superior prognostic resolution.

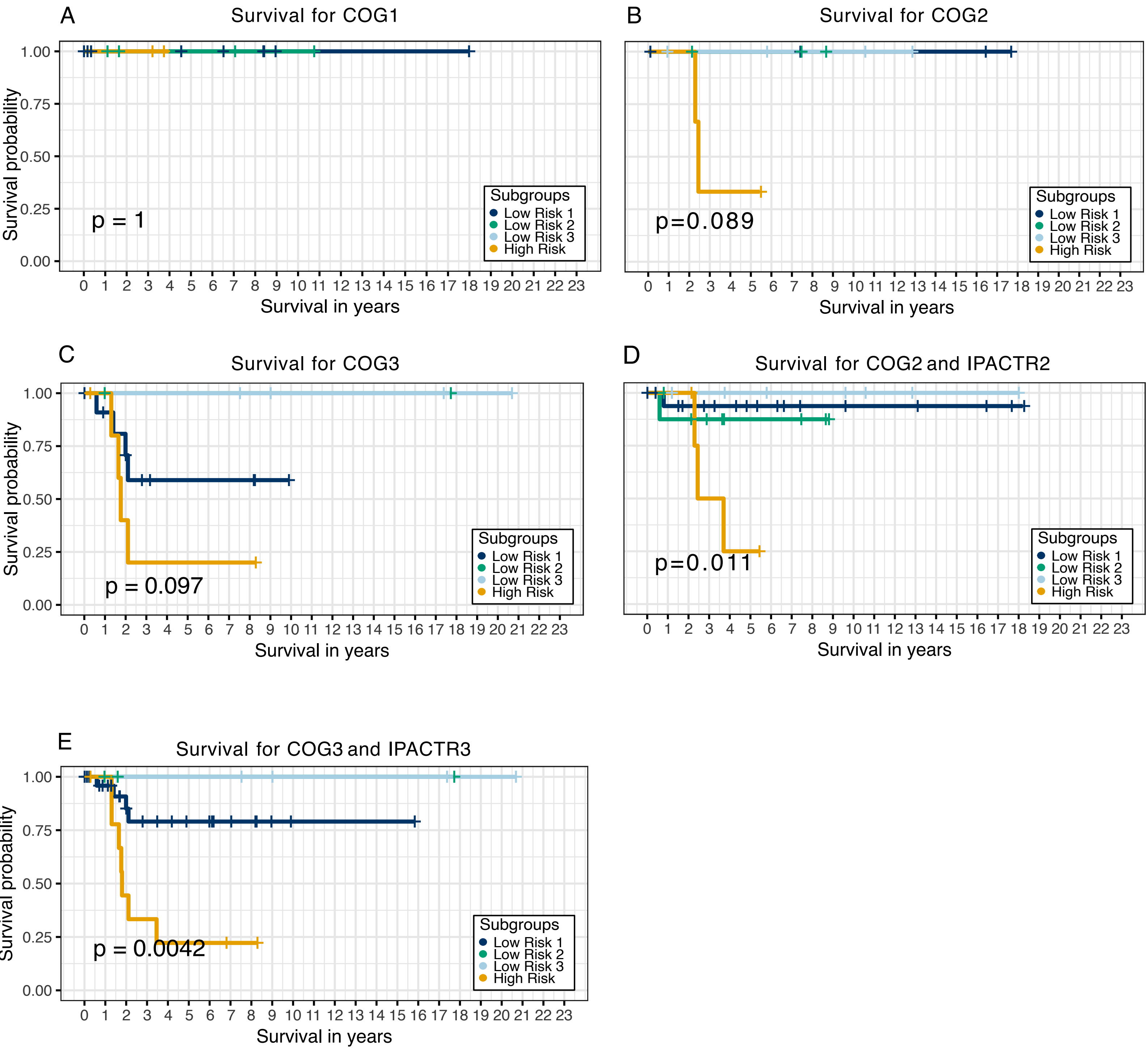
A
Survival for COG1
Survival probability
1.00
0.75
0.50
0.25
0.00
p = 1
Subgroups
Low Risk 1
Low Risk 2
Low Risk 3
High Risk
0 1 2 3 4 5 6 7 8 9 10 11 12 13 14 15 16 17 18 19 20 21 22 23
Survival in years
B
Survival for COG2
p=0.089
Subgroups
Low Risk 1
Low Risk 2
Low Risk 3
High Risk
0 1 2 3 4 5 6 7 8 9 10 11 12 13 14 15 16 17 18 19 20 21 22 23
Survival in years
C
Survival for COG3
Survival probability
1.00
0.75
0.50
0.25
0.00
p = 0.097
Subgroups
Low Risk 1
Low Risk 2
Low Risk 3
High Risk
0 1 2 3 4 5 6 7 8 9 10 11 12 13 14 15 16 17 18 19 20 21 22 23
Survival in years
D
Survival for COG2 and IPACTR2
p=0.011
Subgroups
Low Risk 1
Low Risk 2
Low Risk 3
High Risk
0 1 2 3 4 5 6 7 8 9 10 11 12 13 14 15 16 17 18 19 20 21 22 23
Survival in years
E
Survival for COG3 and IPACTR3
Survival probability
1.00
0.75
0.50
0.25
0.00
p = 0.0042
Subgroups
Low Risk 1
Low Risk 2
Low Risk 3
High Risk
0 1 2 3 4 5 6 7 8 9 10 11 12 13 14 15 16 17 18 19 20 21 22 23
Survival in years

Supplementary Figure S5. Prognostic Stratification of Methylation-Based Subgroups Across COG-Staged Pediatric ACC Cohorts.

(A–C) Kaplan–Meier survival analyses of pediatric ACC patients stratified by COG stage: Stage I (COG1, n=15, A), Stage II (COG2, n=14, B), and Stage III (COG3, n=24, C). DNA methylation-based subgroups (Low Risk 1–3, High Risk) are overlaid to evaluate prognostic resolution within each clinical stage. (D–E) Pooled analysis of patients from COG2 with IPACTR2 (n=39) (D) and COG3 with IPACTR3 (n=44) (E) cohorts demonstrates that DNA methylation subgroups further stratify patient outcomes within identical clinical stages, with significantly worse prognosis observed for High Risk tumors (log-rank $p = 0.011$ and $p = 0.0042$, respectively).

A

	auc	misclassification	brier score	logloss	accuracy
raw scores	0,981613169	0,088495575	0,292092473	0,6013197	0,911504425
calibrated scores	0,989312704	0,066371681	0,127069059	0,263374088	0,933628319

B

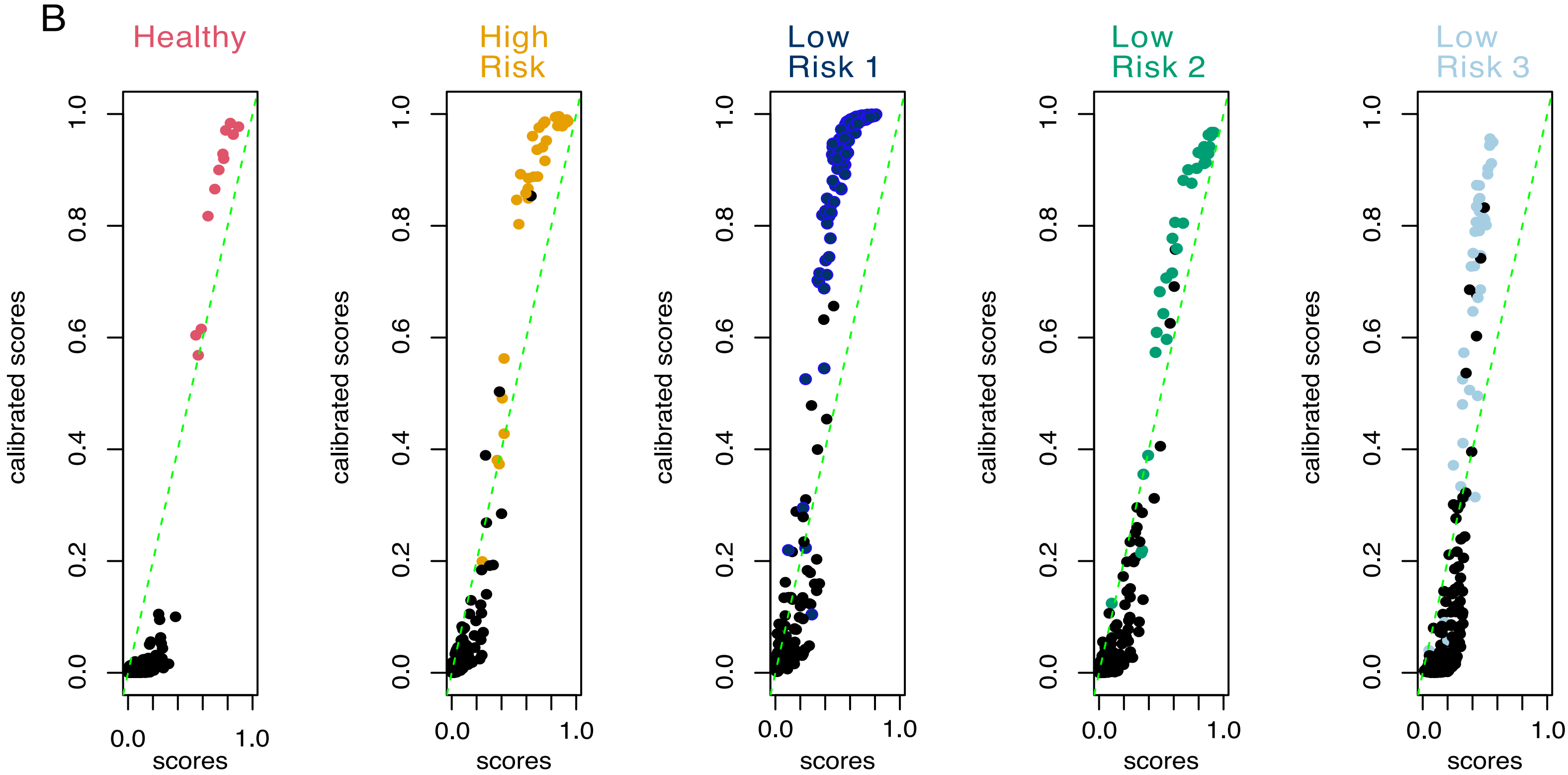

C

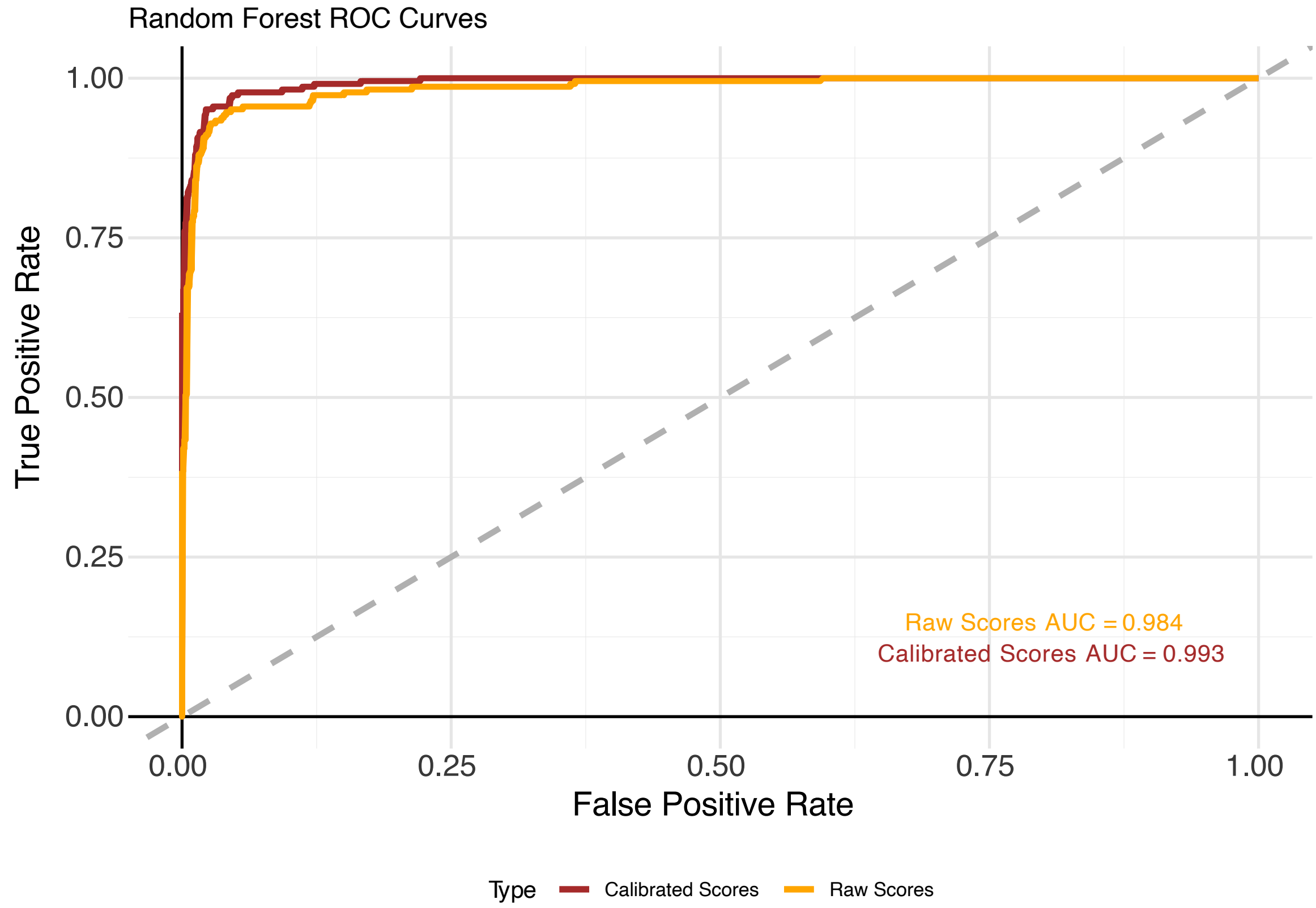

Supplementary Figure S6. Random Forest–Based Classifier Accurately Predicts Methylation-Based pACT Subgroups.

(A) Performance metrics of the random forest classifier trained on DNA methylation profiles from the full cohort of pACT and healthy adrenal reference samples. Calibrated scores significantly improved classification accuracy (93.4%) and reduced log-loss and Brier score relative to raw outputs.
(B) Calibration plots for each subgroup (total n=226; Healthy n=12; HR, n = 36; LR1, n = 104; LR2, n = 34; LR3, n = 40) show probability estimates from the classifier (x-axis) against calibrated scores (y-axis), indicating robust class separation and well-calibrated predictions. Colored dots indicate true positives.
(C) Receiver operating characteristic (ROC) curves for raw and calibrated classifier scores show excellent performance across subgroups (AUC = 0.984 for raw scores, AUC = 0.993 for calibrated scores), demonstrating strong discriminatory power of the model.
This supervised classifier enables objective assignment of subgroup membership and offers a scalable tool for clinical risk stratification in pediatric adrenocortical tumors.

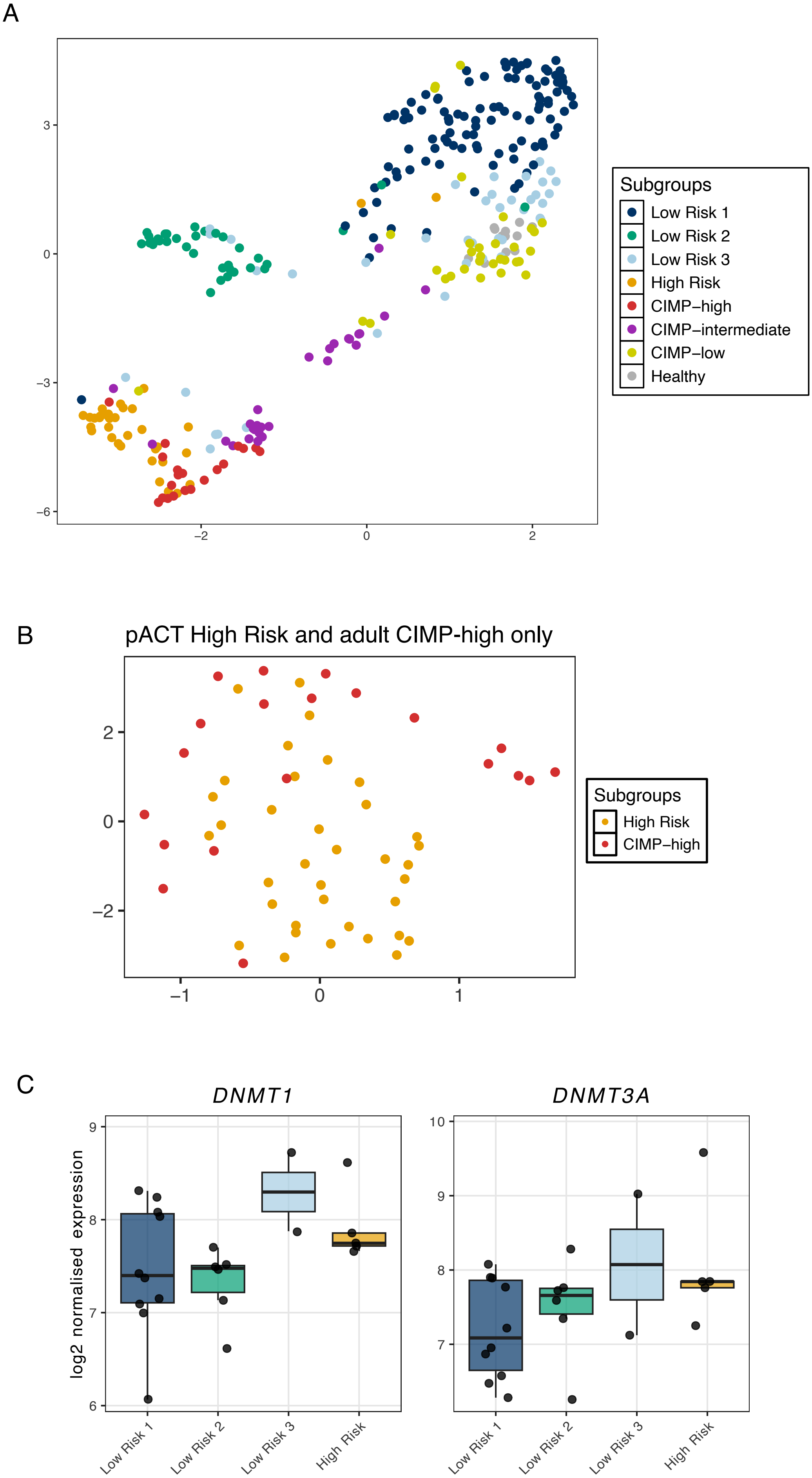

A
Subgroups
Low Risk 1
Low Risk 2
Low Risk 3
High Risk
CIMP–high
CIMP–intermediate
CIMP–low
Healthy
3
0
−3
−6
−2
0
2
B
pACT High Risk and adult CIMP-high only
Subgroups
High Risk
CIMP–high
2
0
−2
−1
0
1
C
DNMT1
DNMT3A
log2 normalised expression
9
8
7
6
10
9
8
7
Low Risk 1
Low Risk 2
Low Risk 3
High Risk

Supplementary Figure S7. Transcriptional and Epigenetic Parallels Between Pediatric High Risk pACTs and Adult CIMP-High ACCs.

(A) UMAP embedding of DNA methylation profiles from pediatric ACTs, adult ACCs and healthy adrenal cortex, colored by DNA methylation-based risk group and CIMP status. Samples cluster according to CIMP intensity and clinical classification, with High Risk pACTs aligning closely with adult CIMP-high ACCs (total n=305, Healthy n=12; HR, n = 36; LR1, n = 104; LR2, n = 34; LR3, n = 40;CIMP-high, n = 20; CIMP-intermediate, n = 27; CIMP-low, n = 32).
(B) UMAP highlighting only pediatric High Risk (n=36) and adult CIMP-high (n=20) ACC samples, showing DNA methylation pattern proximity across these entities.
(C) DNMT1 and DNMT3A expression in the molecular subgroups (n=23). Box plots show the median and interquartile range, whiskers extend to 1.5 × IQR, with individual samples overlaid. Only significant comparisons are annotated (* p.adj < 0.05, ** p.adj < 0.01, *** p.adj < 0.001, ****p.adj < 0.0001)(two-sided Wilcoxon rank-sum tests, Benjamini–Hochberg correction applied independently per gene across six pairwise comparisons). Source data are provided as a Source Data file.

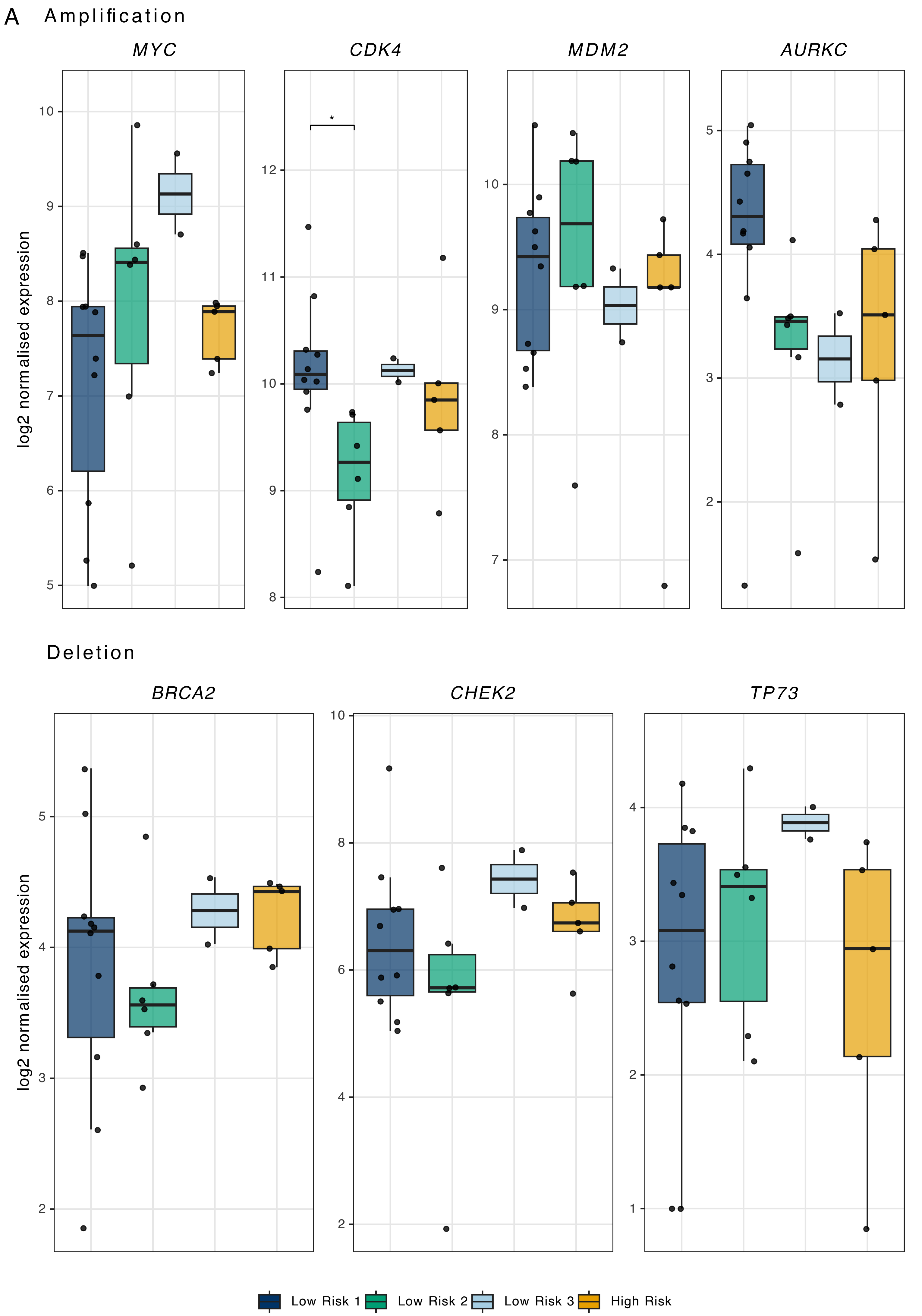
A
Amplification
MYC
CDK4
MDM2
AURKC
log2 normalised expression
*
Deletion
BRCA2
CHEK2
TP73
log2 normalised expression
Low Risk 1
Low Risk 2
Low Risk 3
High Risk

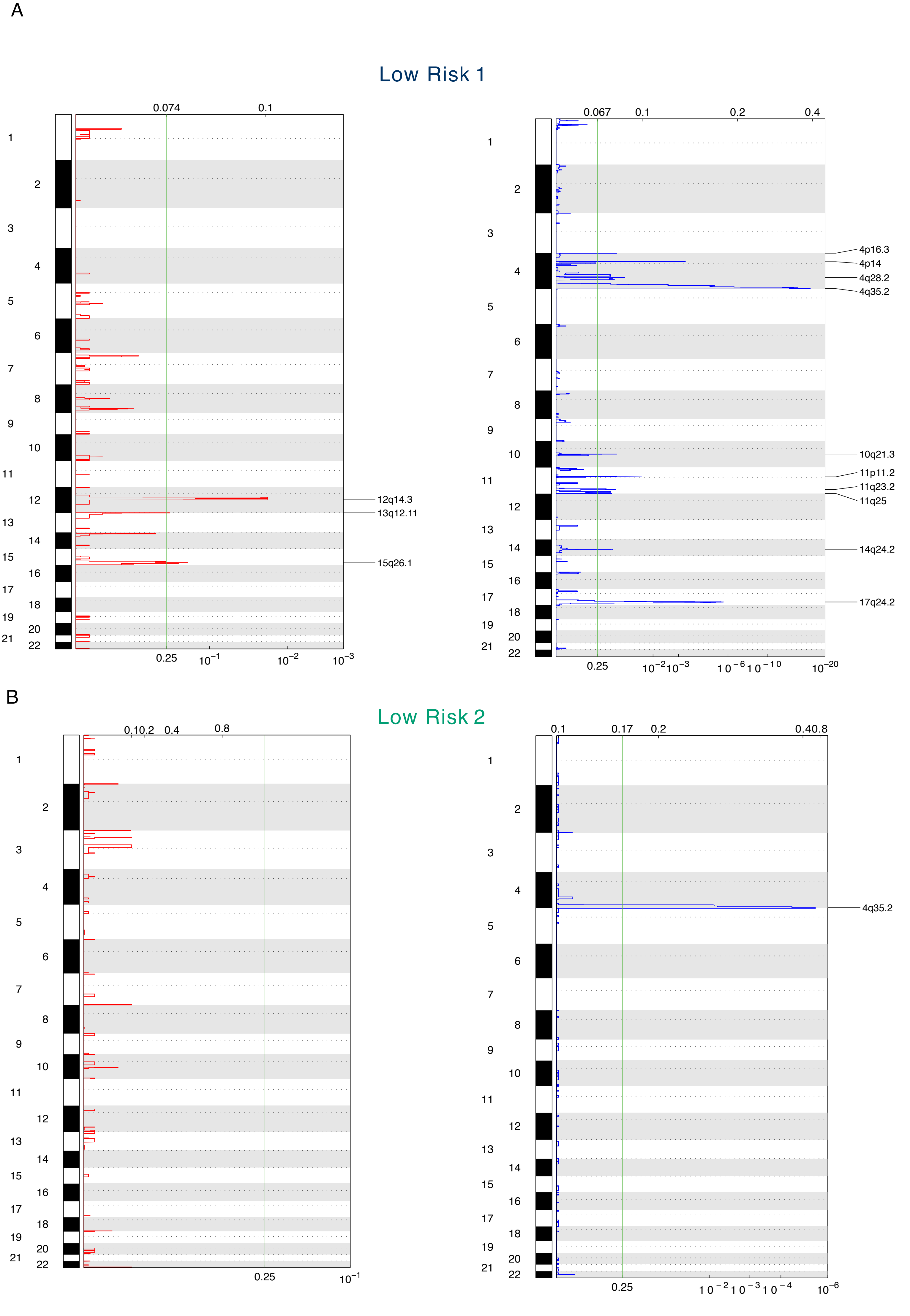
A
Low Risk 1
0.074
0.1
0.25
10^−1
10^−2
10^−3
12q14.3
13q12.11
15q26.1
0.067
0.1
0.2
0.4
10^−2 10^−3
10^−6 10^−10
10^−20
4p16.3
4p14
4q28.2
4q35.2
10q21.3
11p11.2
11q23.2
11q25
14q24.2
17q24.2
B
Low Risk 2
0.1 0.2
0.4
0.8
0.25
10^−1
0.1
0.17
0.2
0.4 0.8
10^−2 10^−3 10^−4
10^−6
4q35.2

C

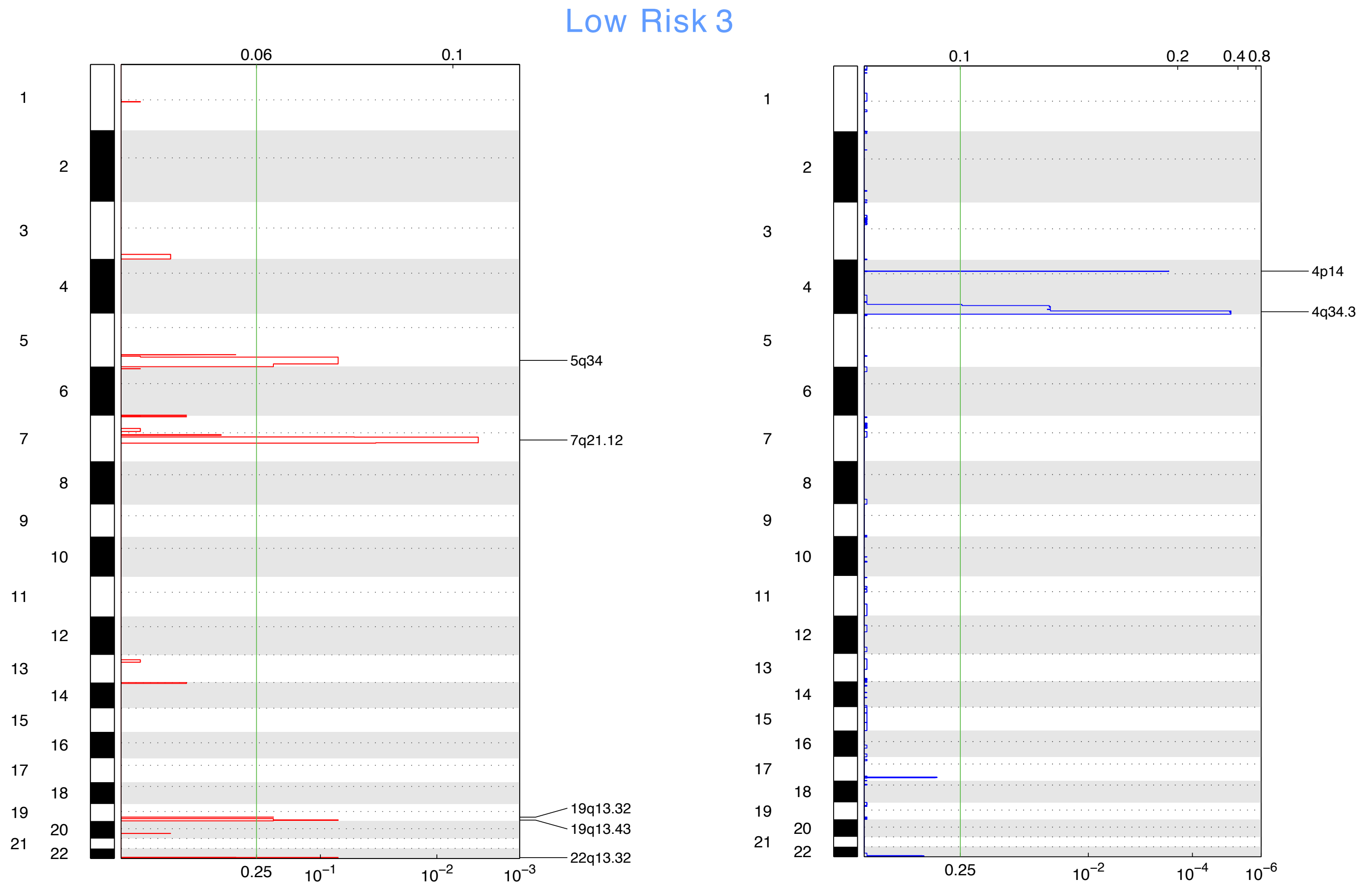

Low Risk 3
0.06
0.1
5q34
7q21.12
19q13.32
19q13.43
22q13.32
0.25
10^−1
10^−2
10^−3
0.1
0.2
0.4
0.8
4p14
4q34.3
0.25
10^−2
10^−4
10^−6

D

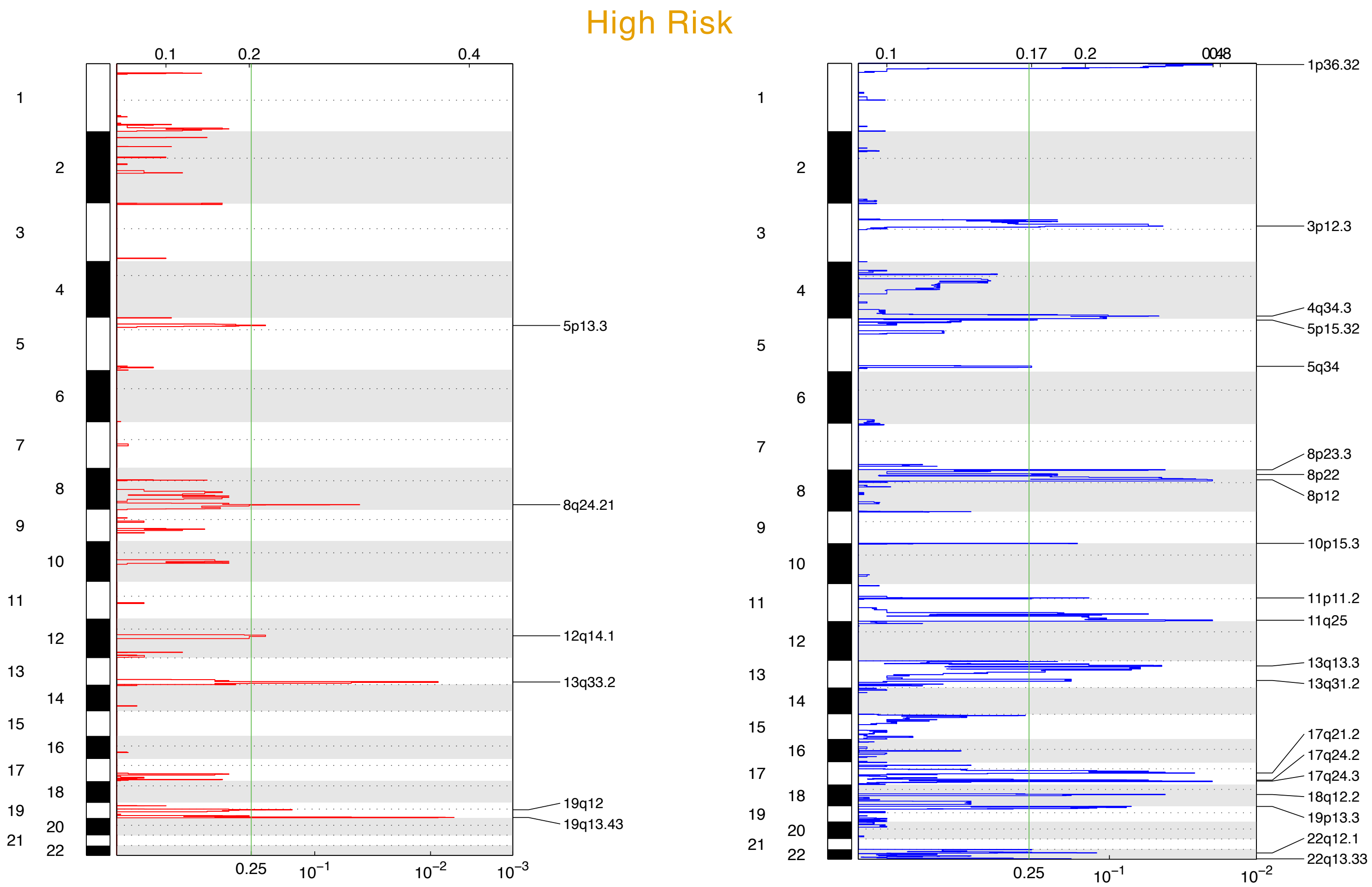

High Risk
0.1
0.2
0.4
5p13.3
8q24.21
12q14.1
13q33.2
19q12
19q13.43
0.25
10^−1
10^−2
10^−3
0.1
0.17
0.2
0.48
1p36.32
3p12.3
4q34.3
5p15.32
5q34
8p23.3
8p22
8p12
10p15.3
11p11.2
11q25
13q13.3
13q31.2
17q21.2
17q24.2
17q24.3
18q12.2
19p13.3
22q12.1
22q13.33
0.25
10^−1
10^−2

Supplementary Figure S8. DNA Methylation–Derived Copy Number Alterations Reveal Subgroup-Specific Genomic Instability.

(A) Expression of candidate genes located in recurrently amplified (*MYC, CDK4, MDM2, AURKC*) and recurrently deleted (*BRCA2, CHEK2, TP73*) regions across the four molecular subgroups (n=23). Box plots show the median and interquartile range, whiskers extend to 1.5 × IQR, and individual samples are overlaid as jittered points. Groups were compared by two-sided Wilcoxon rank-sum tests across all six pairwise subgroup combinations, with Benjamini–Hochberg correction applied independently within each gene. Only significant comparisons are annotated (* p.adj < 0.05, ** p.adj < 0.01, *** p.adj < 0.001, **** p.adj < 0.0001). Source data are provided as a Source Data file.

(B–E)Frequency plots of inferred copy number alterations (CNAs) across the genome, derived from DNA methylation data, for each pACT subgroup: Low Risk 1 (B), Low Risk 2 (C), Low Risk 3 (D), and High Risk (E) (total n=214; HR, n = 36; LR1, n = 104; LR2, n = 34; LR3, n = 40). Genomic coordinates are plotted on the x-axis (chromosomes 1–22), with gains (red) and losses (blue) shown on the y-axis as a percentage of samples affected.

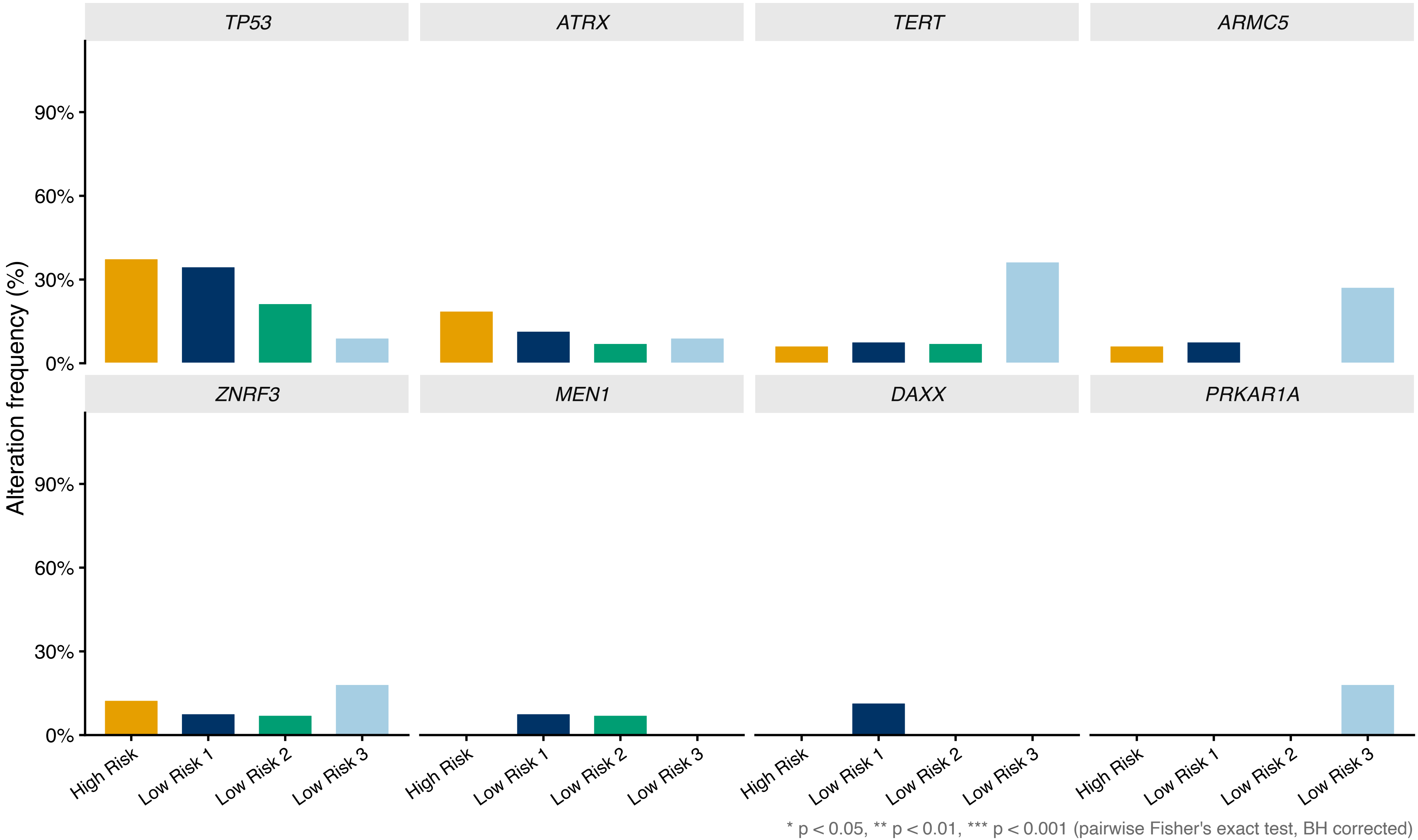
TP53
ATRX
TERT
ARMC5
ZNRF3
MEN1
DAXX
PRKAR1A
Alteration frequency (%)
0%
30%
60%
90%
High Risk
Low Risk 1
Low Risk 2
Low Risk 3
* p < 0.05, ** p < 0.01, *** p < 0.001 (pairwise Fisher's exact test, BH corrected)

Supplementary Figure S9. Alteration frequency of selected driver genes across DNA methylation-defined risk subgroups. Bar plots showing the percentage of patients harboring somatic alterations in eight candidate driver genes - *TP53, ATRX, TERT, ARMC5, ZNRF3, MEN1, DAXX*, and *PRKAR1A* - within each methylation-defined subgroup (HR, n = 16; LR1, n = 26; LR2, n = 14; LR3, n = 11). Only samples with available whole-exome sequencing data are included (n = 67 total). Variants were restricted to likely pathogenic classes (missense, nonsense, frameshift, splice-site, and in-frame indels). Pairwise Fisher's exact tests with Benjamini-Hochberg correction were performed between all subgroup pairs; no statistically significant differences in alteration frequency were observed for any gene across subgroups, likely reflecting the limited sample size available for sequencing.

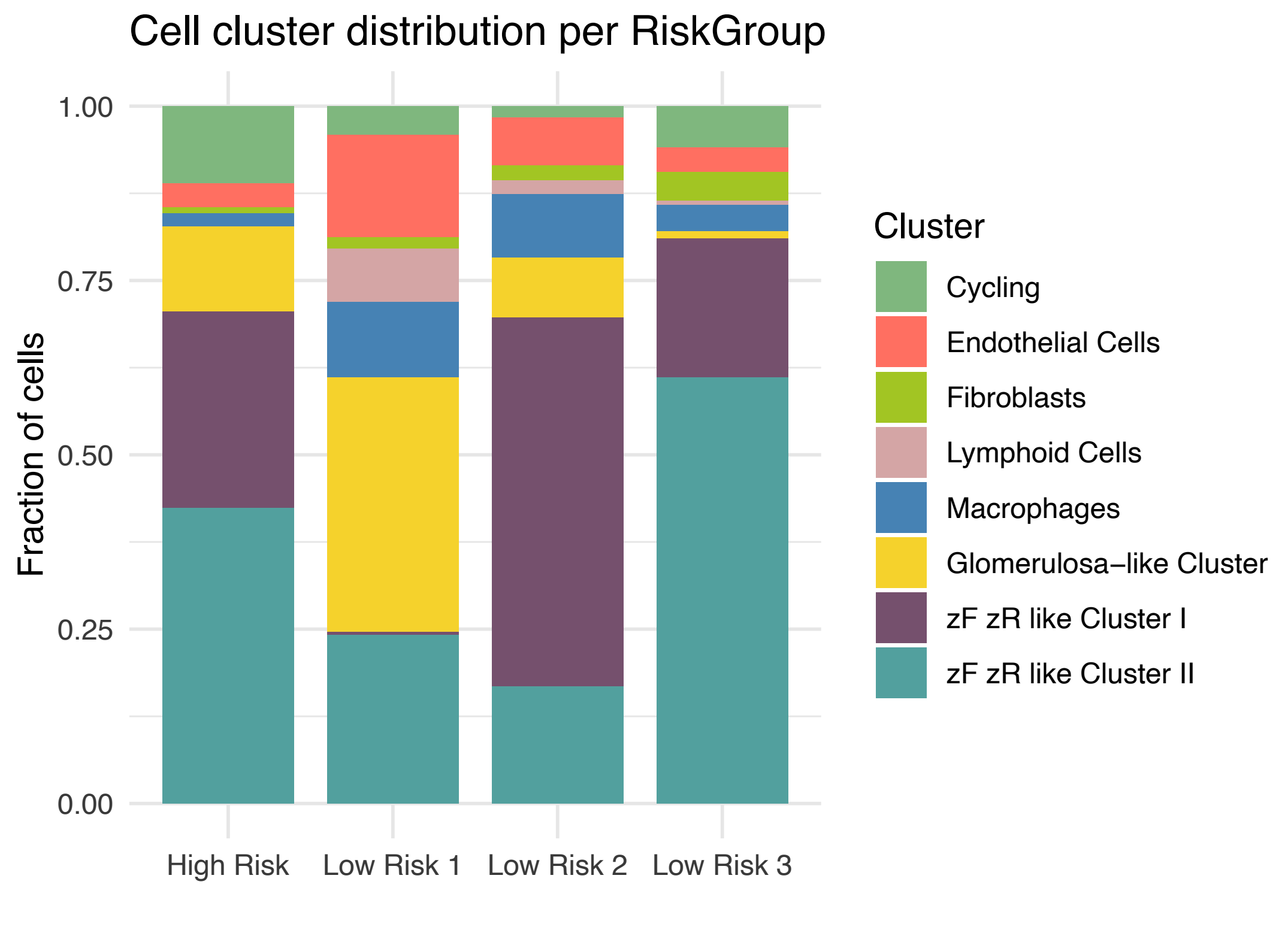
Cell cluster distribution per RiskGroup
Fraction of cells
1.00
0.75
0.50
0.25
0.00
High Risk
Low Risk 1
Low Risk 2
Low Risk 3
Cluster
Cycling
Endothelial Cells
Fibroblasts
Lymphoid Cells
Macrophages
Glomerulosa–like Cluster
zF zR like Cluster I
zF zR like Cluster II

Supplementary Figure S10. Boxplots displaying the proportions of cell type per sample per risk group for tumor and microenvironment cells (High Risk n=13,216 cells, Low Risk 1 n=41,393 cells, Low Risk 2 n=13,001 cells, Low Risk 3 n=7,905 cells) .

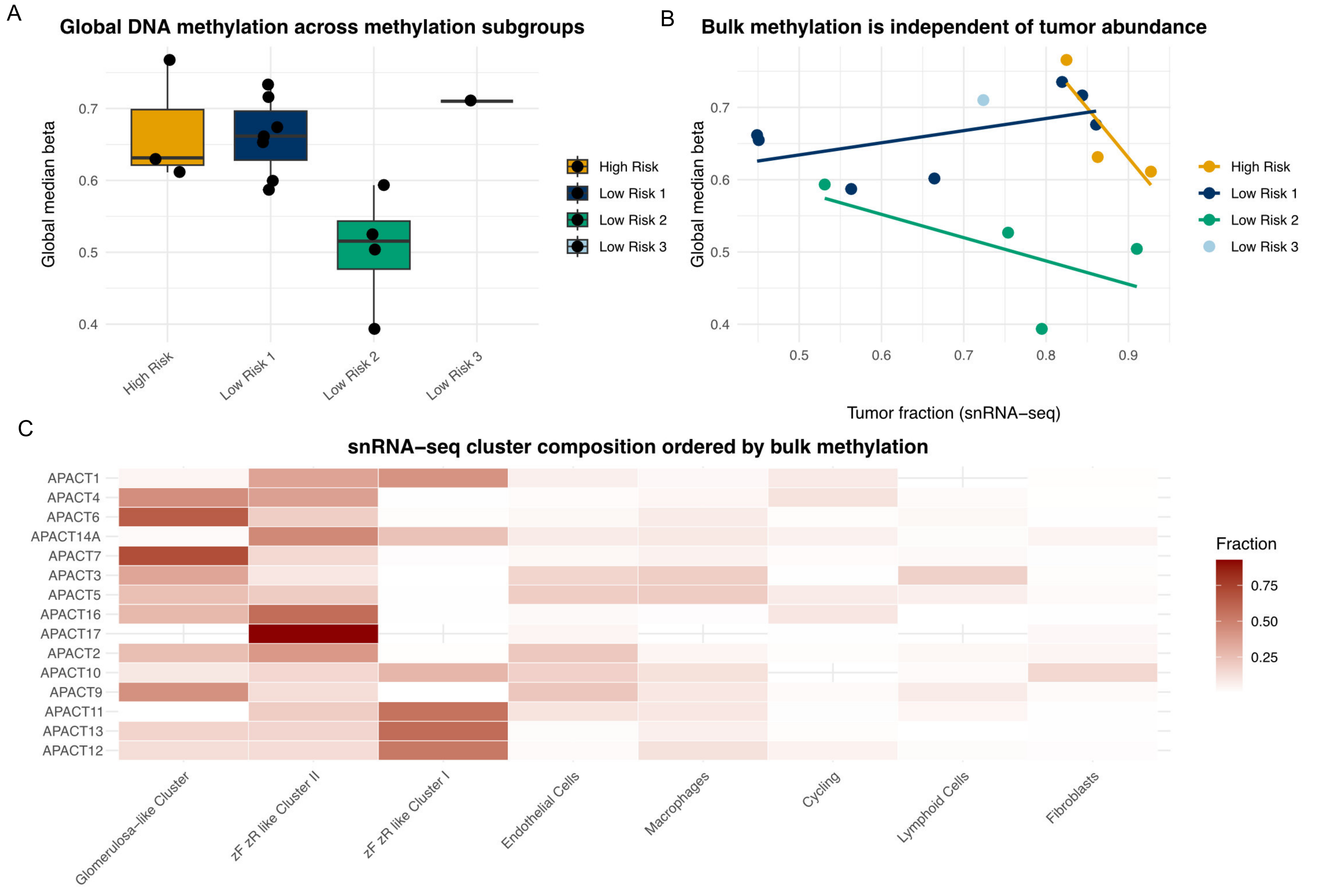
A
Global DNA methylation across methylation subgroups
Global median beta
0.4
0.5
0.6
0.7
High Risk
Low Risk 1
Low Risk 2
Low Risk 3
B
Bulk methylation is independent of tumor abundance
Global median beta
Tumor fraction (snRNA−seq)
0.5
0.6
0.7
0.8
0.9
C
snRNA−seq cluster composition ordered by bulk methylation
APACT1
APACT4
APACT6
APACT14A
APACT7
APACT3
APACT5
APACT16
APACT17
APACT2
APACT10
APACT9
APACT11
APACT13
APACT12
Glomerulosa−like Cluster
zF zR like Cluster II
zF zR like Cluster I
Endothelial Cells
Macrophages
Cycling
Lymphoid Cells
Fibroblasts
Fraction
0.25
0.50
0.75

Supplementary Figure S11: Bulk DNA methylation is independent of tumor cellular composition. (A) Boxplot showing global DNA methylation levels (median beta value across all CpGs) across the four methylation-defined subgroups, demonstrating that HR (n=3) and LR1 (n=7) tumors exhibit higher global methylation compared to LR2 (n=4) and LR3 (n=1). Box plots indicate the median (centre line), the 25th and 75th percentiles (box bounds) and whiskers extending to the most extreme value within 1.5 × the interquartile range of the box, which define the plotted minima and maxima. Individual data points are not shown.(B) Scatter plot showing the relationship between global median beta value and tumor cell fraction estimated from matched snRNA-seq data, stratified by subgroup (n = 15 samples with matched bulk DNA methylation and snRNA-seq data; HR, n = 3; LR1, n = 7; LR2, n = 4; LR3, n = 1). Regression lines are shown per subgroup.

No significant correlation between global methylation and tumor cell abundance was observed across the cohort (Spearman $\rho = 0.06$, $p = 0.82$), indicating that bulk methylation levels are not driven by differences in tumor purity. (C) Heatmap displaying the proportional abundance of individual snRNA-seq clusters - including tumor cell states (Glomerulosa-like, zF/zR-like Cluster I and II, Cycling) and microenvironmental populations (Endothelial Cells, Macrophages, Lymphoid Cells, Fibroblasts) - for each sample ordered by increasing global bulk methylation level (n = 15 samples, same cohort as panel B). No consistent association between methylation level and the composition of any individual cluster was observed, supporting the conclusion that subgroup-specific methylation differences reflect tumor-intrinsic epigenetic programs rather than compositional variation.

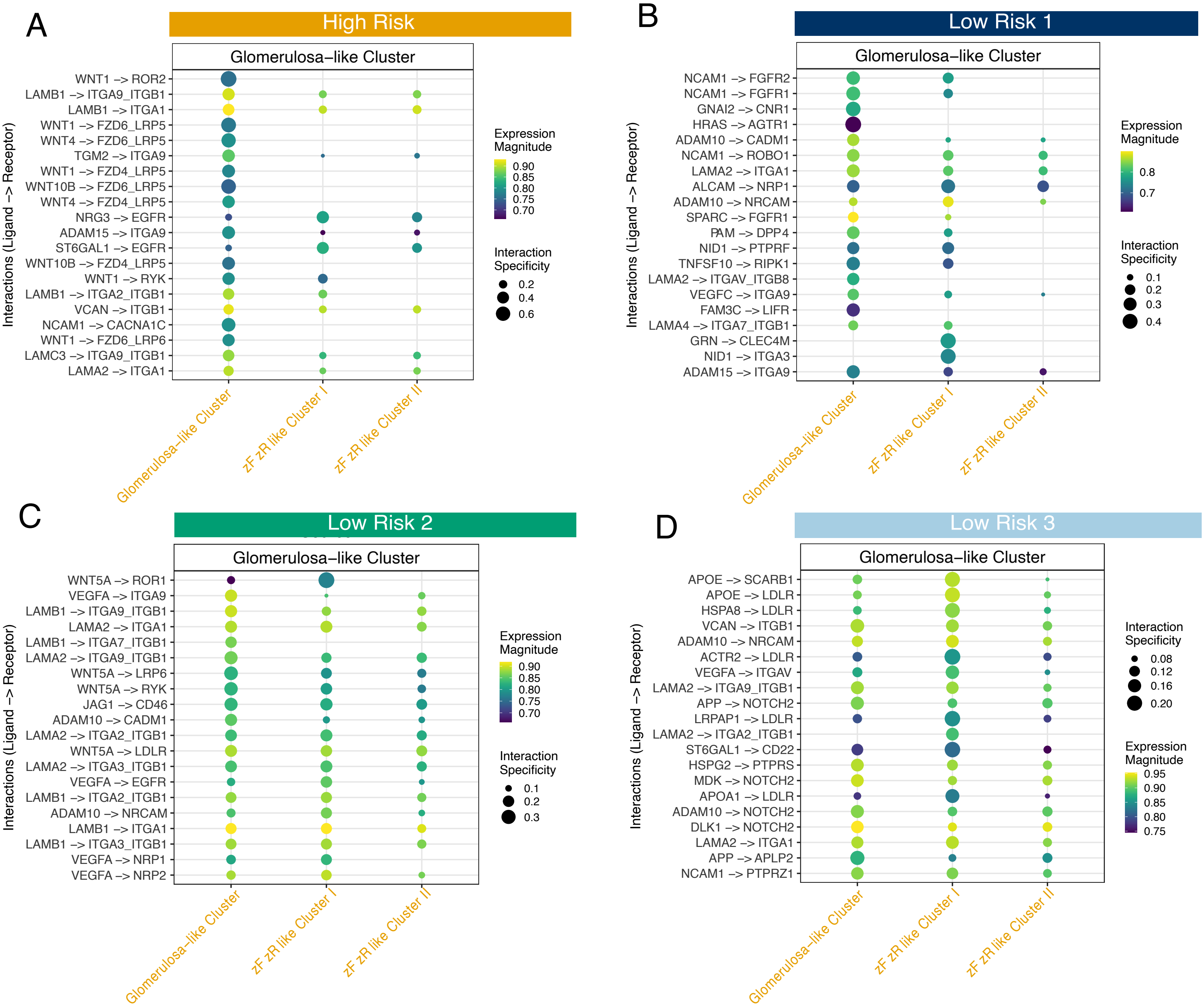

A
High Risk
Glomerulosa–like Cluster
WNT1 –> ROR2
LAMB1 –> ITGA9_ITGB1
LAMB1 –> ITGA1
WNT1 –> FZD6_LRP5
WNT4 –> FZD6_LRP5
TGM2 –> ITGA9
WNT1 –> FZD4_LRP5
WNT10B –> FZD6_LRP5
WNT4 –> FZD4_LRP5
NRG3 –> EGFR
ADAM15 –> ITGA9
ST6GAL1 –> EGFR
WNT10B –> FZD4_LRP5
WNT1 –> RYK
LAMB1 –> ITGA2_ITGB1
VCAN –> ITGB1
NCAM1 –> CACNA1C
WNT1 –> FZD6_LRP6
LAMC3 –> ITGA9_ITGB1
LAMA2 –> ITGA1
Interactions (Ligand –> Receptor)
Glomerulosa–like Cluster
zF zR like Cluster I
zF zR like Cluster II
Expression Magnitude
0.90
0.85
0.80
0.75
0.70
Interaction Specificity
0.2
0.4
0.6
B
Low Risk 1
Glomerulosa–like Cluster
NCAM1 –> FGFR2
NCAM1 –> FGFR1
GNAI2 –> CNR1
HRAS –> AGTR1
ADAM10 –> CADM1
NCAM1 –> ROBO1
LAMA2 –> ITGA1
ALCAM –> NRP1
ADAM10 –> NRCAM
SPARC –> FGFR1
PAM –> DPP4
NID1 –> PTPRF
TNFSF10 –> RIPK1
LAMA2 –> ITGAV_ITGB8
VEGFC –> ITGA9
FAM3C –> LIFR
LAMA4 –> ITGA7_ITGB1
GRN –> CLEC4M
NID1 –> ITGA3
ADAM15 –> ITGA9
Interactions (Ligand –> Receptor)
Glomerulosa–like Cluster
zF zR like Cluster I
zF zR like Cluster II
Expression Magnitude
0.8
0.7
Interaction Specificity
0.1
0.2
0.3
0.4
C
Low Risk 2
Glomerulosa–like Cluster
WNT5A –> ROR1
VEGFA –> ITGA9
LAMB1 –> ITGA9_ITGB1
LAMA2 –> ITGA1
LAMB1 –> ITGA7_ITGB1
LAMA2 –> ITGA9_ITGB1
WNT5A –> LRP6
WNT5A –> RYK
JAG1 –> CD46
ADAM10 –> CADM1
LAMA2 –> ITGA2_ITGB1
WNT5A –> LDLR
LAMA2 –> ITGA3_ITGB1
VEGFA –> EGFR
LAMB1 –> ITGA2_ITGB1
ADAM10 –> NRCAM
LAMB1 –> ITGA1
LAMB1 –> ITGA3_ITGB1
VEGFA –> NRP1
VEGFA –> NRP2
Interactions (Ligand –> Receptor)
Glomerulosa–like Cluster
zF zR like Cluster I
zF zR like Cluster II
Expression Magnitude
0.90
0.85
0.80
0.75
0.70
Interaction Specificity
0.1
0.2
0.3
D
Low Risk 3
Glomerulosa–like Cluster
APOE –> SCARB1
APOE –> LDLR
HSPA8 –> LDLR
VCAN –> ITGB1
ADAM10 –> NRCAM
ACTR2 –> LDLR
VEGFA –> ITGAV
LAMA2 –> ITGA9_ITGB1
APP –> NOTCH2
LRPAP1 –> LDLR
LAMA2 –> ITGA2_ITGB1
ST6GAL1 –> CD22
HSPG2 –> PTPRS
MDK –> NOTCH2
APOA1 –> LDLR
ADAM10 –> NOTCH2
DLK1 –> NOTCH2
LAMA2 –> ITGA1
APP –> APLP2
NCAM1 –> PTPRZ1
Interactions (Ligand –> Receptor)
Glomerulosa–like Cluster
zF zR like Cluster I
zF zR like Cluster II
Interaction Specificity
0.08
0.12
0.16
0.20
Expression Magnitude
0.95
0.90
0.85
0.80
0.75

Supplementary Figure S12. Ligand–Receptor Interactions in Glomerulosa-like Tumor Cells Reveal Subgroup-Specific Signaling Patterns.

(A–D) LIANA-inferred ligand–receptor interaction networks originating from glomerulosa-like tumor cells, stratified by DNA methylation-based subgroups: High Risk (A, n = 1,623 glomerulosa-like cells), Low Risk 1 (B, n = 15,142 glomerulosa-like cells), Low Risk 2 (C, n = 1,120 glomerulosa-like cells), and Low Risk 3 (D, n = 85 glomerulosa-like cells). The small glomerulosa-like population in LR3 (n = 85) limits the robustness of inferred interactions in this subgroup

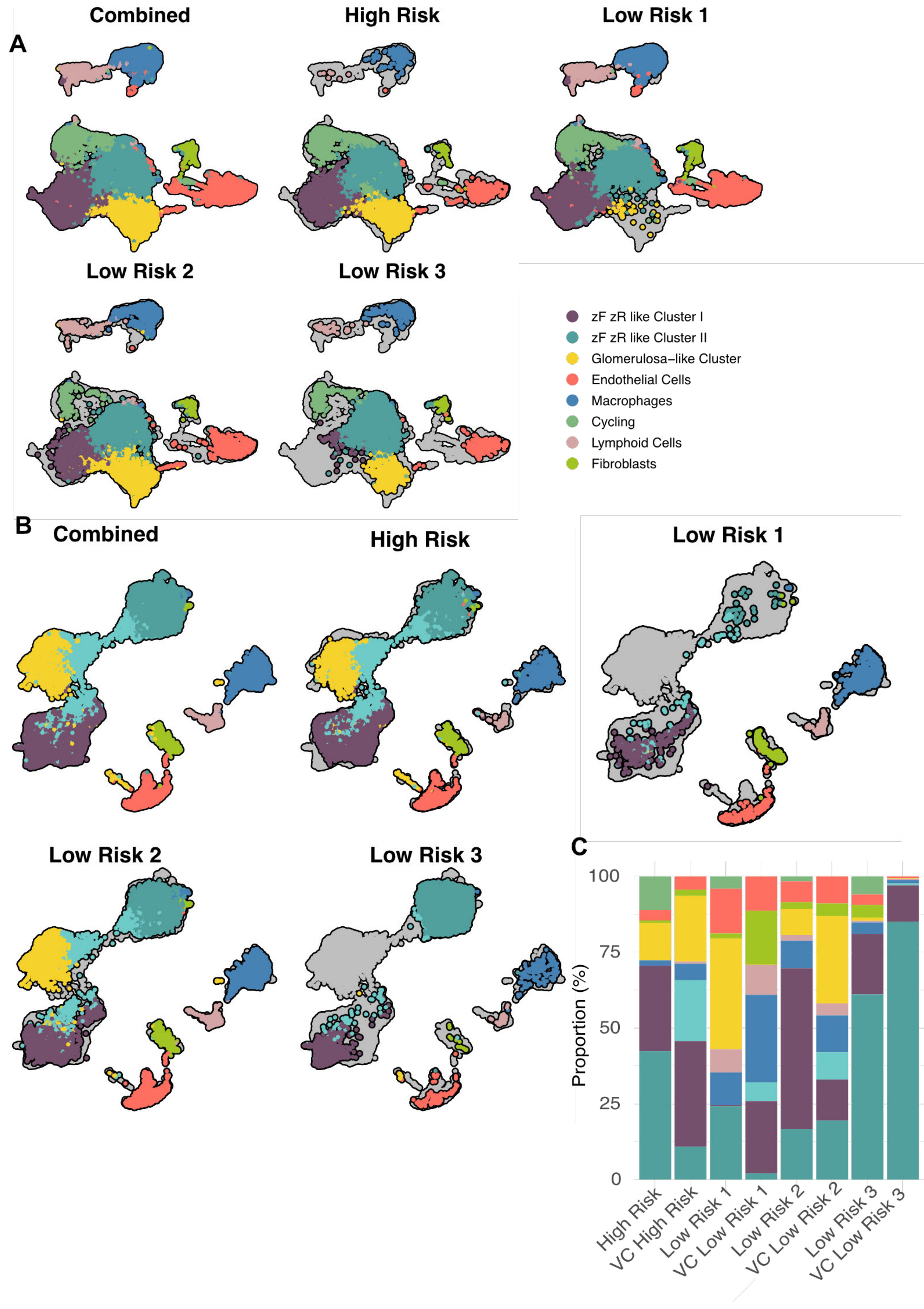
A
Combined
High Risk
Low Risk 1
Low Risk 2
Low Risk 3
zF zR like Cluster I
zF zR like Cluster II
Glomerulosa–like Cluster
Endothelial Cells
Macrophages
Cycling
Lymphoid Cells
Fibroblasts
B
Combined
High Risk
Low Risk 1
Low Risk 2
Low Risk 3
C
Proportion (%)
100
75
50
25
0
High Risk
VC High Risk
Low Risk 1
VC Low Risk 1
Low Risk 2
VC Low Risk 2
Low Risk 3
VC Low Risk 3

Supplementary Figure S13. Validation of Tumor and Microenvironment Cell Type Composition Across Cohorts.

(A and B) UMAP plots showing annotated cell types derived from single-nucleus RNA sequencing (snRNA-seq) data across the primary cohort (A, n=18 samples, n=75,515 nuclei) and the validation cohort (B, n=10 samples, n = 50,988 nuclei),

stratified by DNA methylation-based risk group ((High Risk, n = 29,460; Low Risk 1, n = 45,521; Low Risk 2, n = 27,480; Low Risk 3, n = 24,042 nuclei, combined across both cohorts)). Shared cell types were annotated using previously established markers and include zF/zR-like tumor cells (Clusters I and II), glomerulosa-like tumor cells, cycling tumor cells, and cells of the tumor microenvironment (macrophages, lymphoid cells, fibroblasts, and endothelial cells).

(C) Stacked bar plots summarizing the proportional distribution of cell types per tumor, grouped by risk category and cohort (VC: validation cohort). Primary cohort: High Risk, n = 4 samples; Low Risk 1, n = 8 samples; Low Risk 2, n = 4 samples; Low Risk 3, n = 2 samples (75,336 nuclei total — High Risk, n = 13,037; Low Risk 1, n = 41,393; Low Risk 2, n = 13,001; Low Risk 3, n = 7,905 nuclei). Validation cohort: High Risk, n = 4 samples; Low Risk 1, n = 3 samples; Low Risk 2, n = 3 samples; Low Risk 3, n = 1 sample (50,799 nuclei total — High Risk, n = 16,184; Low Risk 1, n = 4,021; Low Risk 2, n = 14,458; Low Risk 3, n = 16,136 nuclei). High Risk tumors showed a heterogeneous cellular composition enriched for glomerulosa-like, cycling, and immune/stromal cell types, while Low Risk 3 tumors were dominated by a single tumor population. The validation cohort Low Risk 3 group is represented by a single sample (n = 1), and its cell type distribution should therefore be interpreted as a single-tumor observation rather than a representative group average. The overall distribution patterns were consistent across primary and validation cohorts, supporting reproducibility of cell type annotations and tumor architecture.

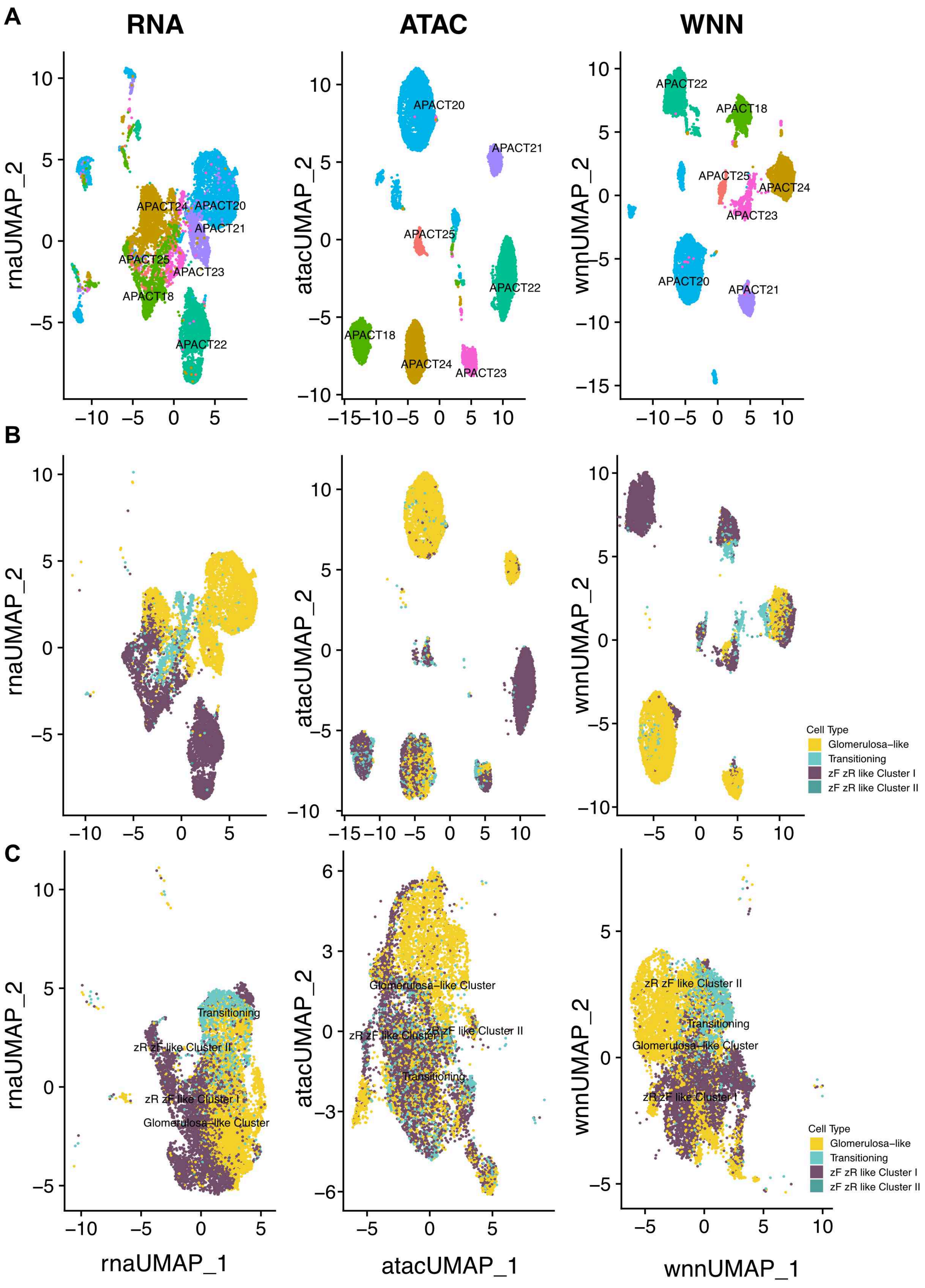

A
B
C
RNA
ATAC
WNN
rnaUMAP_2
atacUMAP_2
wnnUMAP_2
rnaUMAP_1
atacUMAP_1
wnnUMAP_1
APACT18
APACT20
APACT21
APACT22
APACT23
APACT24
APACT25
Cell Type
Glomerulosa–like
Transitioning
zF zR like Cluster I
zF zR like Cluster II
Glomerulosa–like Cluster
zR zF like Cluster I
zR zF like Cluster II
Transitioning

Supplementary Figure S14. Integrated Multimodal Single-Cell Analysis Confirms Robust Cell Type Annotation and Cross-Modality Concordance.

(A) UMAP plots of RNA (left), ATAC (middle), and weighted nearest neighbor (WNN; right) embeddings of matched single-nucleus RNA-seq (snRNA-seq) and single-nucleus ATAC-seq (snATAC-seq) data from 7 pediatric adrenocortical tumors (pACTs). Cells are color-coded by patient ID (APACT25 cells n=359; APACT24 cells n=1909; APACT18 cells n=1217; APACT22 cells n=2311; APACT20 cells n=3682; APACT21 cells n=1030; APACT23 cells n=754) demonstrating clustering consistency across modalities.

(B) UMAP plots of the same datasets (n=7), color-coded by cell type annotation derived from snRNA-seq and projected across modalities without TME. The major annotated tumor populations—glomerulosa-like (cells, n=4683), zF/zR-like Clusters I (cells, n=4112) and II (cells, n=27), and a transitional state (cells, n=925).

(C) Joint UMAP plots (n=7) after Harmony highlighting consistent clustering of core tumor cell types

(glomerulosa-like (cells, n=4683), zF/zR-like Clusters I (cells, n=4112) and II (cells, n=27), and transitioning cells (cells, n=925)) across RNA, ATAC, and WNN modalities, supporting accurate cross-platform cell identity assignment.

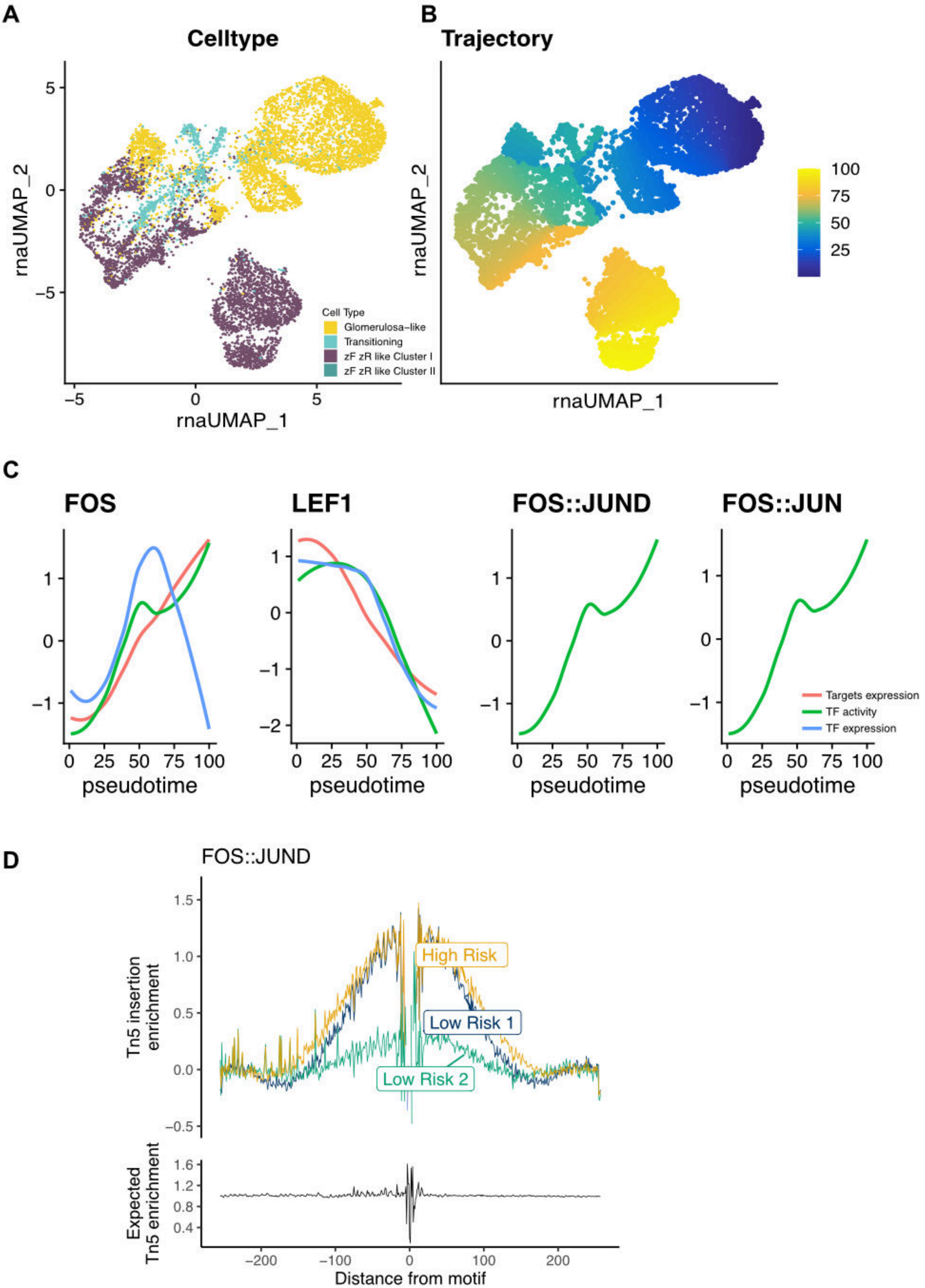

A
Celltype
rnaUMAP_2
rnaUMAP_1
Cell Type
Glomerulosa−like
Transitioning
zF zR like Cluster I
zF zR like Cluster II
B
Trajectory
rnaUMAP_2
rnaUMAP_1
100
75
50
25
C
FOS
LEF1
FOS::JUND
FOS::JUN
pseudotime
Targets expression
TF activity
TF expression
D
FOS::JUND
Tn5 insertion enrichment
High Risk
Low Risk 1
Low Risk 2
Expected Tn5 enrichment
Distance from motif

Supplementary Figure S15. Pseudotemporal Dynamics and Chromatin Accessibility Highlight Transcription Factor Regulation Across Differentiation States in pACT.

(A) UMAP embedding of single-nucleus Multiome data (n=7) from the validation cohort, colored by tumor cell subtype. Tumor cells are grouped into glomerulosa-like (cells, n=4683), zF/zR-like Clusters I (cells, n=4112) and II (cells, n=27), and identified transitioning population (cells, n=925) exhibiting intermediate features.

(B) RNA velocity and pseudotime trajectory analysis inferred a differentiation continuum from glomerulosa-like to zF/zR-like tumor populations. Cells are colored by pseudotime values from early (yellow) to late (blue) states (total cells n=9747).

(C) Pseudotime-resolved transcription factor (TF) activity and expression profiles for WNT-related (LEF1) and AP-1 complex (FOS, FOS::JUND, FOS::JUN) regulators. LEF1 and FOS showed opposing expression dynamics, with LEF1 peaking earlier and FOS later in pseudotime (n=7).

(D) Chromatin accessibility profiling from single-nucleus ATAC-seq (n=7) revealed differential enrichment of Tn5 insertions at FOS::JUND binding motifs across risk groups. High Risk tumors displayed the strongest motif enrichment, followed by Low Risk 1. Low Risk 2 tumors showed minimal accessibility at these regulatory sites. Together, these findings support a model in which WNT and AP-1 transcription factors act in a temporally coordinated manner to shape tumor cell identity and progression in high-risk pACT.

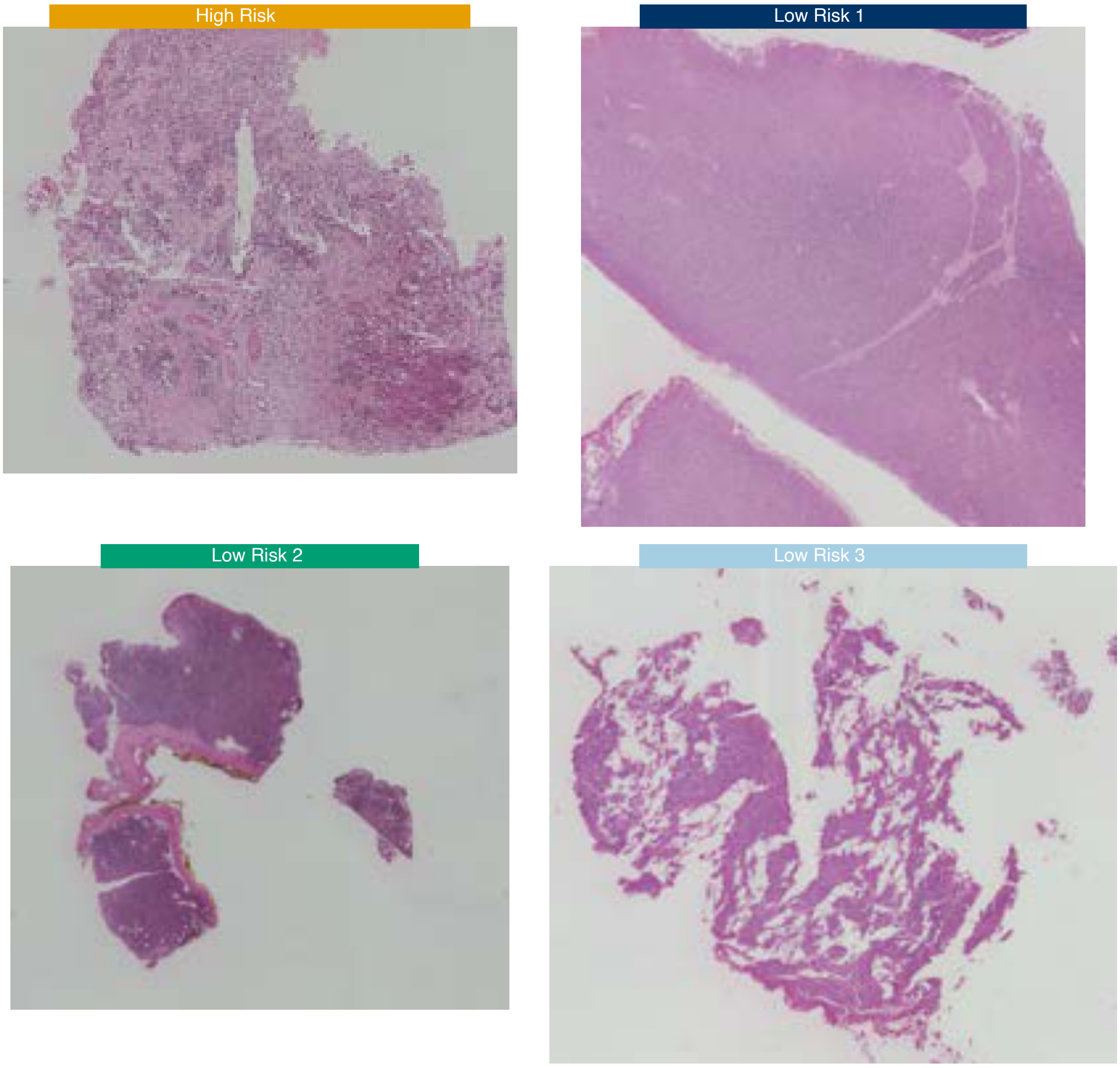
High Risk
Low Risk 1
Low Risk 2
Low Risk 3

Supplementary Figure S16. Histological characterization of tumor samples used for spatial transcriptomics.

Hematoxylin and eosin (H&E) staining of representative sections from four pediatric ACT tumors processed with Visium HD spatial transcriptomics. Tumors span all four molecular subgroups defined by DNA methylation profiling: HR (APACT19), LR1 (APACT4), LR2 (APACT12), and LR3 (APACT27).

Supplementary Figure S17. Spatially resolved expression of *DNMT3A, FOS, JUN* and *JUND*.

Log-normalised expression of each gene in one representative Visium HD per DNA methylation subgroup (one sample per subgroup; Low Risk 1, n = 289,651; Low Risk 2, n = 140,105; Low Risk 3, n = 99,273; High Risk, n = 146,167 bin2cell-reconstructed cells). Colour scale is identical across all panels (0–2). A cell was scored as positive if its log-normalised expression exceeded zero; the number and percentage of positive cells are annotated on each panel. Right-hand column: fraction of positive cells per gene, summarised as a box plot of the three low risk sections (LR, n = 3 sections) with the high risk section overlaid as an individual reference point (HR, n = 1 section). Box plots indicate the median (centre line), the 25th and 75th percentiles (box bounds) and whiskers extending to the most extreme value within 1.5 × the interquartile range of the box, which define the plotted minima and maxima. Because each group comprises one section per subgroup, no statistical testing was performed and the comparison is descriptive.

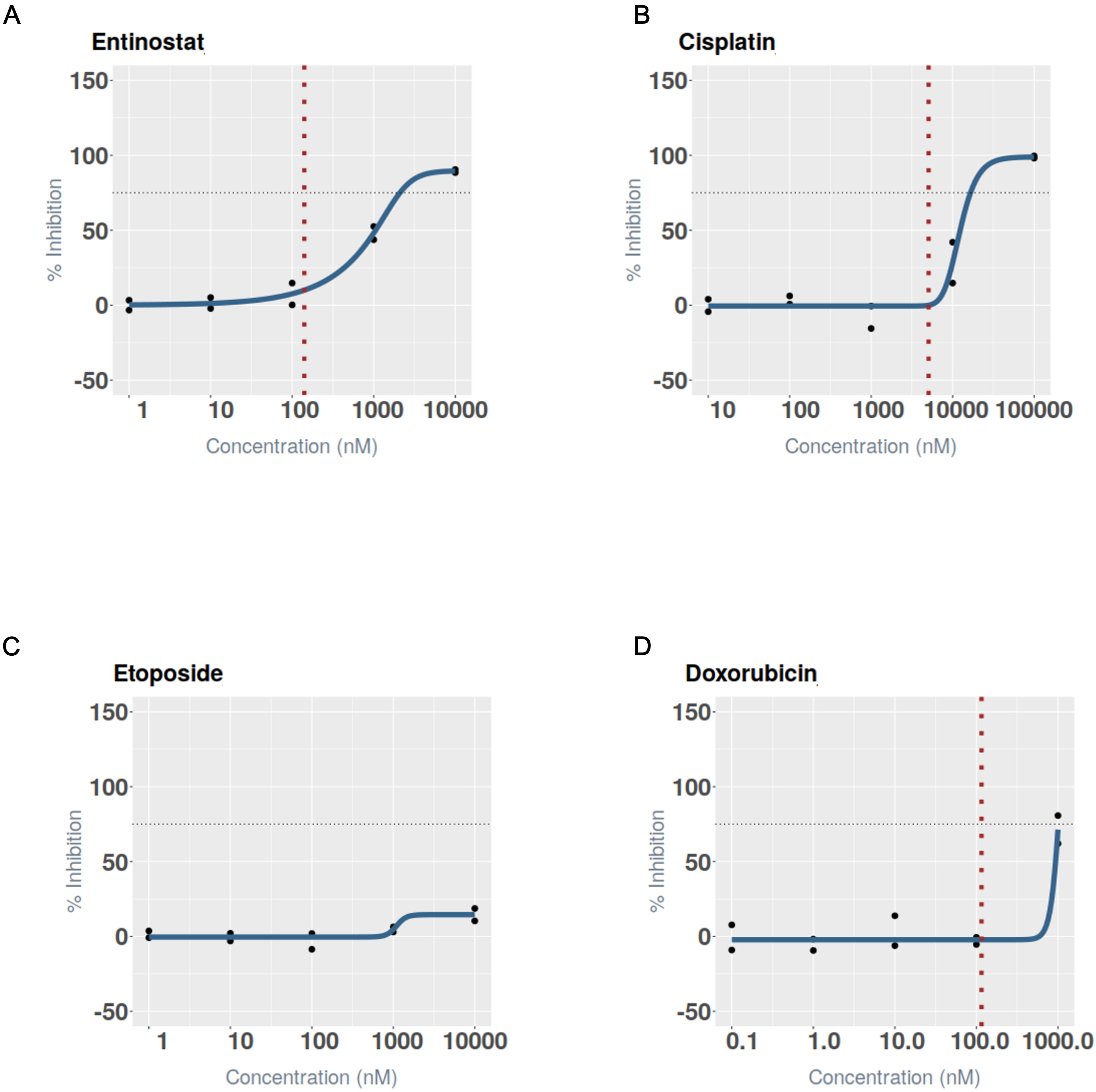

A
Entinostat
150
100
50
0
-50
% Inhibition
1
10
100
1000
10000
Concentration (nM)
B
Cisplatin
150
100
50
0
-50
% Inhibition
10
100
1000
10000
100000
Concentration (nM)
C
Etoposide
150
100
50
0
-50
% Inhibition
1
10
100
1000
10000
Concentration (nM)
D
Doxorubicin
150
100
50
0
-50
% Inhibition
0.1
1.0
10.0
100.0
1000.0
Concentration (nM)

Supplementary Figure S18. Drug response profiling of freshly resected HR pACT in short-term culture.

Viability screening of a short-term culture derived from a freshly resected lung metastasis of a pACC patient (n = 1 patient-derived culture, each concentration tested in technical duplicate).

The sample, classified as HR by DNA methylation profiling, was exposed to a library of clinically relevant anti-cancer compounds across five concentrations. Shown are normalized inhibition values (% inhibition) representing metabolic activity reduction after 72 hours of treatment in 3D spheroid culture format. The red dashed line indicates the maximum serum concentration for the respective drug. No statistical testing was performed, as this experiment was intended to illustrate the feasibility of the approach.

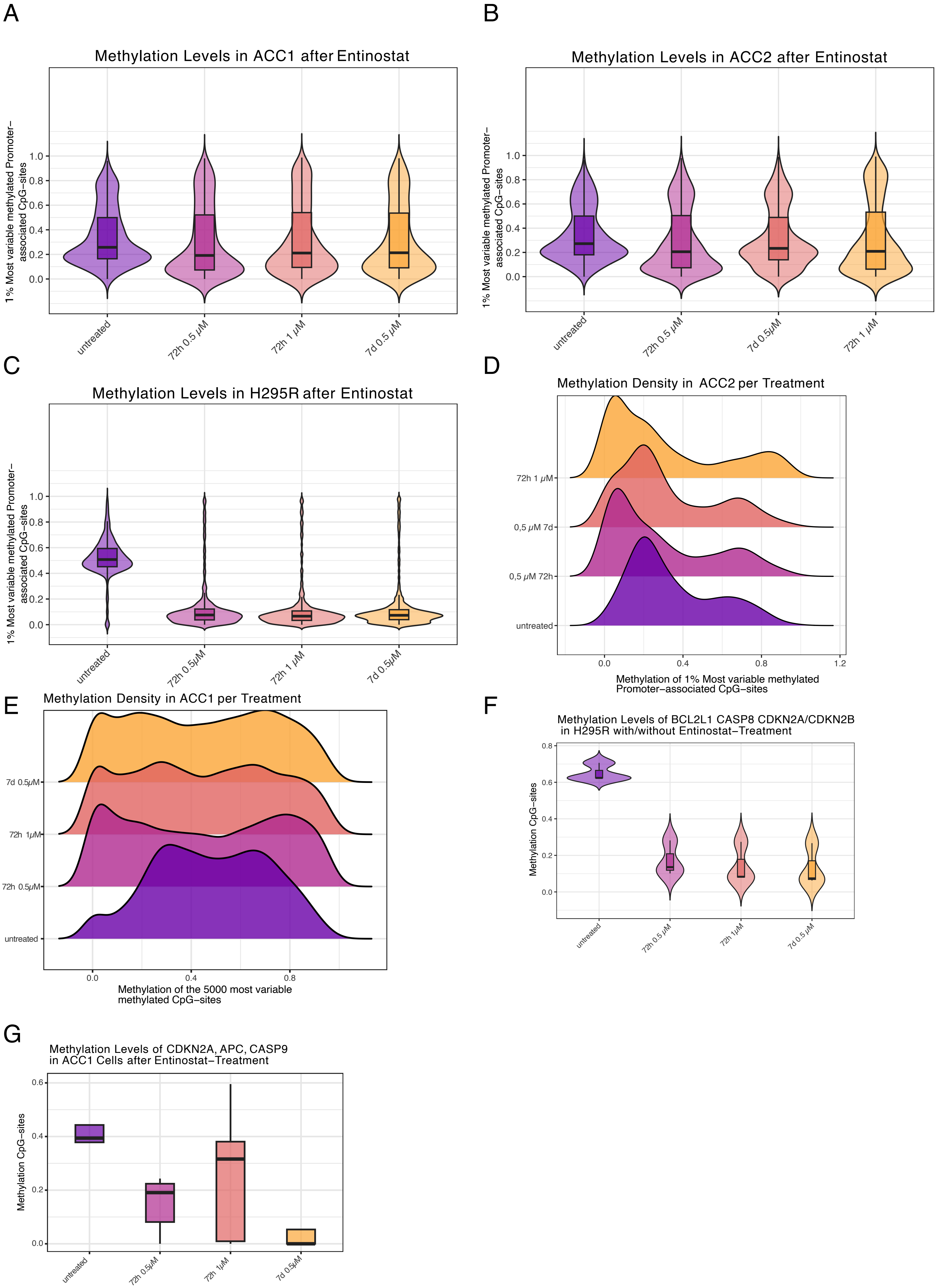
A
Methylation Levels in ACC1 after Entinostat
1% Most variable methylated Promoter–associated CpG–sites
0.0
0.2
0.4
0.6
0.8
1.0
untreated
72h 0.5 µM
72h 1 µM
7d 0.5 µM
B
Methylation Levels in ACC2 after Entinostat
1% Most variable methylated Promoter–associated CpG–sites
0.0
0.2
0.4
0.6
0.8
1.0
untreated
72h 0.5 µM
7d 0.5µM
72h 1 µM
C
Methylation Levels in H295R after Entinostat
1% Most variable methylated Promoter–associated CpG–sites
0.0
0.2
0.4
0.6
0.8
1.0
untreated
72h 0.5µM
72h 1 µM
7d 0.5µM
D
Methylation Density in ACC2 per Treatment
72h 1 µM
0,5 µM 7d
0,5 µM 72h
untreated
0.0
0.4
0.8
1.2
Methylation of 1% Most variable methylated Promoter–associated CpG–sites
E
Methylation Density in ACC1 per Treatment
7d 0.5µM
72h 1µM
72h 0.5µM
untreated
0.0
0.4
0.8
Methylation of the 5000 most variable methylated CpG–sites
F
Methylation Levels of BCL2L1 CASP8 CDKN2A/CDKN2B in H295R with/without Entinostat–Treatment
Methylation CpG–sites
0.0
0.2
0.4
0.6
0.8
untreated
72h 0.5 µM
72h 1µM
7d 0.5 µM
G
Methylation Levels of CDKN2A, APC, CASP9 in ACC1 Cells after Entinostat–Treatment
Methylation CpG–sites
0.0
0.2
0.4
0.6
untreated
72h 0.5µM
72h 1µM
7d 0.5µM

Supplementary Figure S19. Entinostat inducesglobal andlocus-specific DNA demethylation in ACC cell lines.

(A–C) Violin plots showing DNA methylation levels at the top 1% most variably methylated promoter-associated CpG sites (n = 1,000 CpG sites per condition) in ACC1 (A), H295R (B) and ACC2 (C) cells after entinostat treatment (0.5 μM and 1 μM for 72 h; 0.5 μM for 7 days) compared with untreated controls. Each condition represents a single experiment (n = 1); as no biological replicates were performed, no statistical testing was conducted and the comparison is descriptive.

Box plots indicate the median (centre line), the 25th and 75th percentiles (box bounds) and whiskers extending to the most extreme value within 1.5 × the interquartile range of the box, which define the plotted minima and maxima. Individual data points are not shown. Source data are provided as a Source Data file.

(D–E) Ridge plots showing methylation density across the 5,000 most variably methylated CpG sites under each treatment condition in ACC2 (D) and ACC1 (E). Each condition represents a single experiment (n = 1); as no biological replicates were performed, no statistical testing was conducted and the comparison is descriptive. Box plots indicate the median (centre line), the

25th and 75th percentiles (box bounds) and whiskers extending to the most extreme value within 1.5 × the interquartile range of the box, which define the plotted minima and maxima. Individual data points are not shown.

(F–G) Violin plots showing methylation levels at pro-apoptotic and tumour suppressor gene loci: BCL2L1 (n = 47 CpG sites), CASP8 (n = 44 CpG sites), and CDKN2A/CDKN2B (n = 38 and n = 12 CpG sites, respectively) in H295R (F), and CDKN2A (n = 38 CpG sites), APC (n = 55 CpG sites), and CASP9 (n = 34 CpG sites) in ACC1 (G). Each condition represents a single experiment (n = 1); as no biological replicates were performed, no statistical testing was conducted and the comparison is descriptive. Box plots indicate the median (centre line), the 25th and 75th percentiles (box bounds) and whiskers extending to the most extreme value within 1.5 × the interquartile range of the box, which define the plotted minima and maxima.

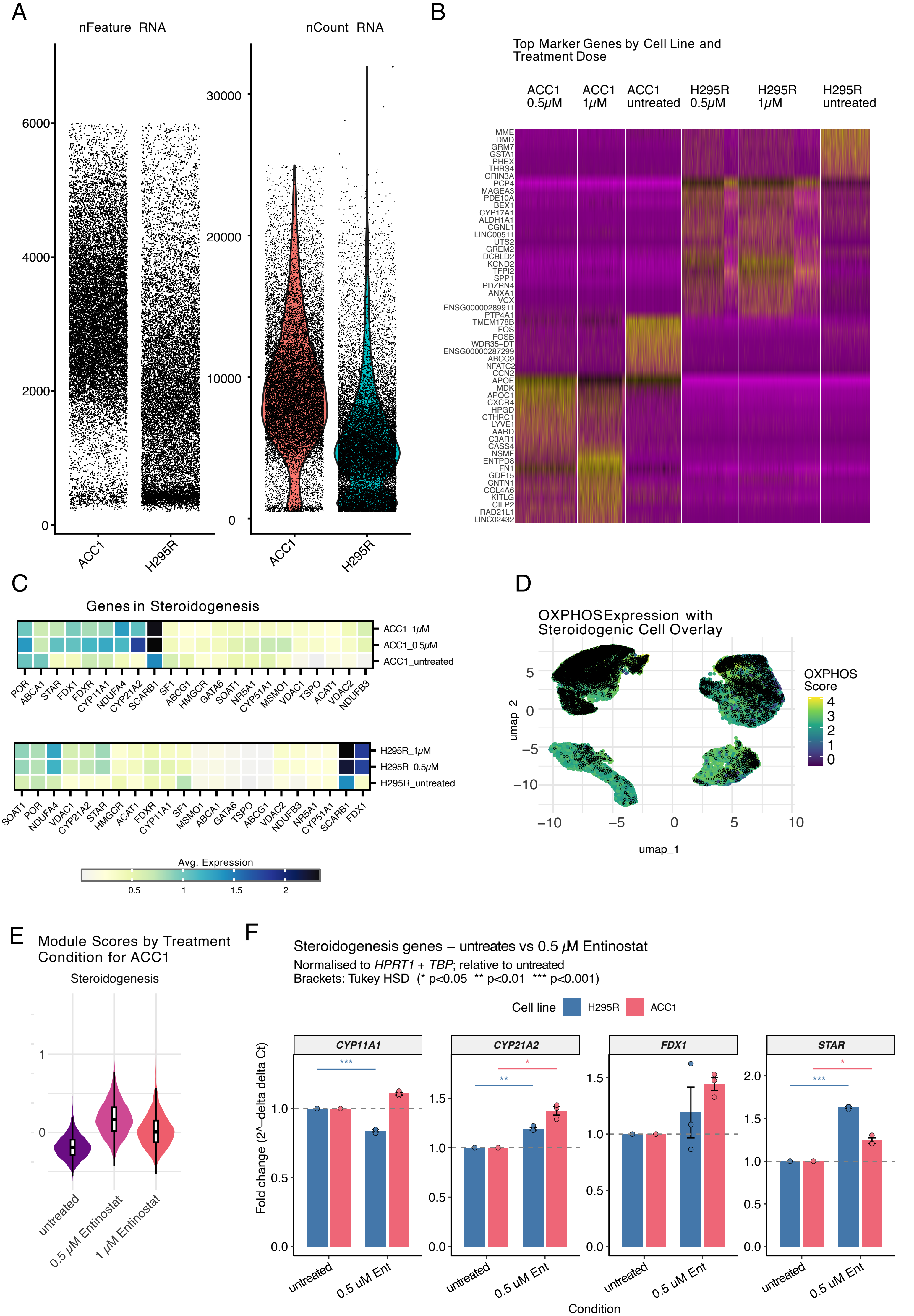

A
nFeature_RNA
nCount_RNA
ACC1
H295R
B
Top Marker Genes by Cell Line and Treatment Dose
ACC1 0.5μM
ACC1 1μM
ACC1 untreated
H295R 0.5μM
H295R 1μM
H295R untreated
MME DMD GRM7 GSTA1 PHEX THBS4 GRIN3A PCP4 MAGEA3 PDE10A BEX1 CYP17A1 ALDH1A1 CGNL1 LINC00511 UTS2 GREM2 DCBLD2 KCND2 TFPI2 SPP1 PDZRN4 ANXA1 VCX ENSG00000289911 PTP4A1 TMEM178B FOS FOSB WDR35−DT ENSG00000287299 ABCC9 NFATC2 CCN2 APOE MDK APOC1 CXCR4 HPGD CTHRC1 LYVE1 AARD C3AR1 CASS4 NSMF ENTPD8 FN1 GDF15 CNTN1 COL4A6 KITLG CILP2 RAD21L1 LINC02432
C
Genes in Steroidogenesis
ACC1_1μM
ACC1_0.5μM
ACC1_untreated
POR ABCA1 STAR FDX1 FDXR CYP11A1 NDUFA4 CYP21A2 SCARB1 SF1 ABCG1 HMGCR GATA6 SOAT1 NR5A1 CYP51A1 MSMO1 VDAC1 TSPO ACAT1 VDAC2 NDUFB3
H295R_1μM
H295R_0.5μM
H295R_untreated
SOAT1 POR NDUFA4 VDAC1 CYP21A2 STAR HMGCR ACAT1 FDXR CYP11A1 SF1 MSMO1 ABCA1 GATA6 TSPO ABCG1 VDAC2 NDUFB3 NR5A1 CYP51A1 SCARB1 FDX1
Avg. Expression
D
OXPHOS Expression with Steroidogenic Cell Overlay
OXPHOS Score
umap_1
umap_2
E
Module Scores by Treatment Condition for ACC1
Steroidogenesis
untreated
0.5 μM Entinostat
1 μM Entinostat
F
Steroidogenesis genes – untreates vs 0.5 μM Entinostat
Normalised to HPRT1 + TBP; relative to untreated
Brackets: Tukey HSD (* p<0.05 ** p<0.01 *** p<0.001)
Cell line H295R ACC1
CYP11A1
CYP21A2
FDX1
STAR
Fold change (2^−delta delta Ct)
untreated
0.5 uM Ent
Condition

Supplementary Figure S20. Single-cell transcriptomic profiling reveals dose-dependent transcriptional reprogramming in ACC cell lines following HDAC inhibition.

(A) Violin plots showing distributions of RNA features across untreated and entinostat-treated conditions (0.5 µM and 1 µM) for ACC1 and H295R cells, confirming data quality across all groups. Data derive from a single scRNA-seq experiment per condition (n = 1); n indicates the number of cells/nuclei analyzed per condition, not independent experimental replicates: untreated (ACC1, n = 5,138 cells; H295R, n = 4,588 nuclei), 0.5 µM entinostat (ACC1, n = 5,851; H295R, n = 5,251), 1 µM entinostat (ACC1, n = 4,480; H295R, n = 7,693)

(B) Heatmap displaying the top marker genes across each cell line and treatment condition, highlighting distinct transcriptional states associated with HDAC inhibition derived from a single scRNA-seq experiment per condition (n = 1; same dataset as panel A).

(C–D) Plots showing average expression of key steroidogenesis-related genes (e.g., *STAR, CYP11A1, SF1*) across all treatment conditions in ACC1 and H295R. A marked upregulation of steroidogenic programs and an increase of expression of pro-apoptotic genes is observed upon entinostat exposure derived from a single scRNA-seq experiment per condition (n = 1; same dataset as panel A).

(E) Module scores by treatment condition. Violin plots show the kernel density distribution; embedded box plots indicate the median (centre line), 25th and 75th percentiles (box bounds), and whiskers extending to 1.5 × IQR. Data derive from a single experiment per condition (n = 1).

(F) Bar plots show mean fold change (FC = $2^{-\Delta\Delta Ct}$)in mRNA expression of *CYP11A1, CYP21A2, FDX1* and *STAR* after treatment with 0.5µM entinostat for 3 days relative to untreated cells, in H295R (blue) and ACC1 (red) cells. Data represent three independent biological experiments, each performed with technical triplicates; fold-change values shown are averaged across technical replicates within each biological experiment. Error bars denote ± standard error of the mean (SEM) across the three independent experiments (n = 3). The dashed horizontal line indicates FC = 1 (no change). Statistical comparisons were performed using one-way ANOVA followed by Tukey's honestly significant difference (HSD) post-hoc test; this test is two-sided and inherently adjusts for multiple comparisons. Asterisks denote Tukey-adjusted p-values (*p < 0.05, **p < 0.01, ***p < 0.001); bars not marked with an asterisk were not statistically significant.

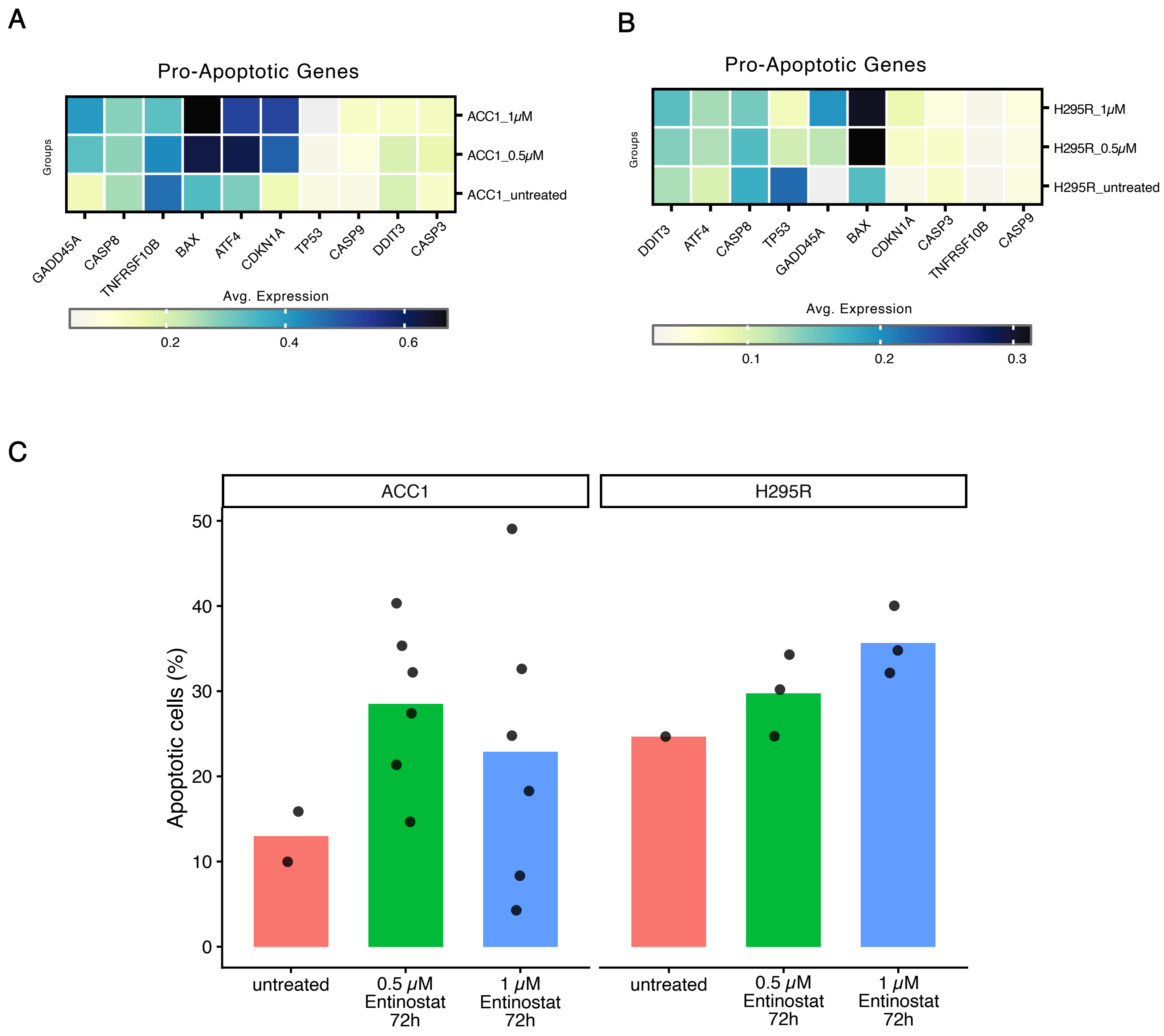

A
Pro-Apoptotic Genes
Groups
ACC1_1μM
ACC1_0.5μM
ACC1_untreated
GADD45A
CASP8
TNFRSF10B
BAX
ATF4
CDKN1A
TP53
CASP9
DDIT3
CASP3
Avg. Expression
0.2
0.4
0.6
B
Pro-Apoptotic Genes
Groups
H295R_1μM
H295R_0.5μM
H295R_untreated
DDIT3
ATF4
CASP8
TP53
GADD45A
BAX
CDKN1A
CASP3
TNFRSF10B
CASP9
Avg. Expression
0.1
0.2
0.3
C
ACC1
H295R
Apoptotic cells (%)
0
10
20
30
40
50
untreated
0.5 μM Entinostat 72h
1 μM Entinostat 72h
untreated
0.5 μM Entinostat 72h
1 μM Entinostat 72h

Supplementary Figure S21. Entinostat induces pro-apoptotic gene expression and apoptosis in ACC cell lines. (A-B) Plots showing average expression of key pro-apoptotic genes (e.g., BAX, TP53) across all treatment conditions in ACC1 and H295R, from a single scRNA-seq experiment performed once per condition. n indicates the number of cells/nuclei analyzed per condition (not independent experimental replicates): untreated (ACC1 n=5,138, H295R n=4,588), 0.5 μM entinostat (ACC1 n=5,851, H295R n=5,251), and 1 μM entinostat (ACC1 n=4,480, H295R n=7,693). An increase in expression of pro-apoptotic genes is observed upon entinostat exposure. As these data derive from a single experiment per condition, no statistical test was performed and no error bars are shown.

(C) Bar plots displaying the fraction of apoptotic cells per cell line and treatment condition, measured by FACS with Annexin V / 7-AAD staining. n indicates the number of independent biological experiments performed per condition: ACC1, untreated n = 2, 0.5 μM n = 6, 1 μM n = 6; H295R, untreated n = 1, 0.5 μM n = 3, 1 μM n = 3.

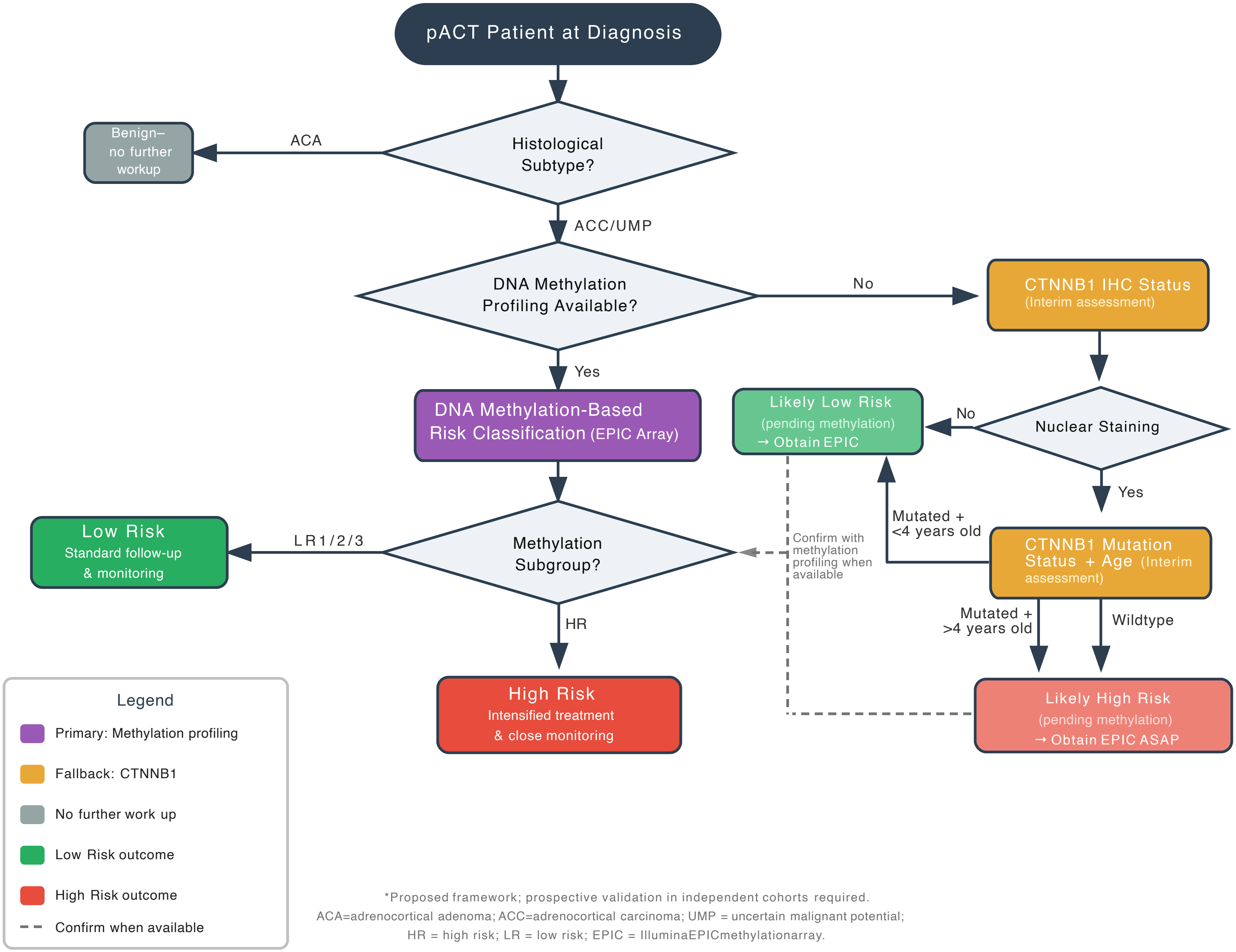
pACT Patient at Diagnosis
Histological Subtype?
ACA
Benign– no further workup
ACC/UMP
DNA Methylation Profiling Available?
No
CTNNB1 IHC Status (Interim assessment)
Yes
DNA Methylation-Based Risk Classification (EPIC Array)
Nuclear Staining
No
Likely Low Risk (pending methylation) → Obtain EPIC
Yes
Mutated + <4 years old
CTNNB1 Mutation Status + Age (Interim assessment)
Methylation Subgroup?
LR1/2/3
Low Risk Standard follow-up & monitoring
Confirm with methylation profiling when available
HR
High Risk Intensified treatment & close monitoring
Mutated + >4 years old
Wildtype
Likely High Risk (pending methylation) → Obtain EPIC ASAP
Legend
Primary: Methylation profiling
Fallback: CTNNB1
No further work up
Low Risk outcome
High Risk outcome
Confirm when available
*Proposed framework; prospective validation in independent cohorts required.
ACA=adrenocortical adenoma; ACC=adrenocortical carcinoma; UMP = uncertain malignant potential;
HR = high risk; LR = low risk; EPIC = IlluminaEPICmethylationarray.

Supplementary Figure S22. Proposed clinical decision tree for risk stratification of pediatric adrenocortical tumor patients at diagnosis.
