# Supplementary material for: CpG hypermethylation and WNT/AP-1 cooperativity define the epigenetic landscape and a clinical subgroup of high-risk pediatric adrenocortical carcinoma
Source: Nat Commun. 2026 Sep 14;17:9786. doi: 10.1038/s41467-026-77225-5 (PMC13575229; doi:10.1038/s41467-026-77225-5)
Supplement: Supplementary file 4 [file 41467_2026_77225_MOESM2_ESM.pdf]

Description of Additional Supplementary Information

Title: Supplementary Data S1.
Description: Clinical, molecular, and demographic characteristics of all pACT patients included in this study, including DNA methylation-based subgroup assignment, histological diagnosis, clinical presentation, survival data and availability of multi-omic data.

Title: Supplementary Data S2.
Description: Annotated somatic variant calls from whole-exome sequencing across pACT samples, including variant classification, functional consequence, population allele frequencies and sequencing metrics.

Title: Supplementary Data S3.
Description: Differentially expressed marker genes for each snRNA-seq cluster identified in the pACT single-nucleus transcriptome analysis, including average log2 fold change, percentage of expressing cells, and adjusted p-values.

Title: Supplementary Data S4.
Description: Cell cycle state distribution across snRNA-seq clusters, including mean S and G2M scores and proportions of cells in G1, S, and G2M phases.

Title: Supplementary Data S5.
Description: Contributing genes for each NMF-derived transcriptional metasignature identified across pACT tumor cell compartments.

Title: Supplementary Data S6.
Description: Supplementary Data S6. Ranked ligand-receptor interactions between cell type pairs in HR pACT tumors as identified by multi-tool cell-cell communication analysis, including aggregate rank scores across NATMI, Connectome, logFC, SCA, and CellPhoneDB methods.

Title: Supplementary Data S7.
Description: Differentially expressed marker genes for each snATAC-seq cluster, including average log2 fold change, percentage of expressing cells, and adjusted p-values.

Title: Supplementary Data S8.
Description: Drug sensitivity profiling results from high-throughput pharmacological screening, including absolute and relative IC50, IC25, and IC75 values across tested compounds. Cmax_KiTZ indicating maximum serum concentration

Title: Supplementary Data S9.
Description: Primer sequences used for quantitative PCR validation of target genes, with primer coordinates, melting temperatures, GC content, and amplicon lengths.
